# Divergent trends in post-landfall decay of strong and weak tropical cyclones

**DOI:** 10.1038/s41467-026-77958-3

**Published:** 2026-09-24

**Authors:** Chenxi Hu, Xinxin Sui, Dev Niyogi, Clint N. Dawson, Zong-Liang Yang

**Affiliations:** 1 Department of Earth and Planetary Sciences, Jackson School of Geosciences, The University of Texas at Austin, Austin, TX USA; 2 National Science Foundation, National Center for Atmospheric Research, Boulder, CO USA; 3 Civil and Environmental Engineering, Colorado School of Mines, Golden, CO USA; 4 Department of Civil, Architectural, and Environmental Engineering, The University of Texas at Austin, Austin, TX USA; 5 Oden Institute for Computational Engineering and Sciences, The University of Texas at Austin, Austin, TX USA; 6 Department of Aerospace Engineering and Engineering Mechanics, The University of Texas at Austin, Austin, TX USA

**Keywords:** Climate sciences, Natural hazards, Climate change

## Abstract

Tropical cyclone post-landfall decay strongly influences inland hazard exposure, yet most studies assume that environmental change affects storms of different intensities uniformly. Here we identify an intensity-dependent divergence in global trends of tropical cyclone post-landfall decay. Analyzing 885 observed events (1979–2024), we show that strong storms (≥29 m/s) now weaken 18.6% faster after landfall, whereas weak storms persist 14.5% longer inland. These contrasting trends arise from different environmental sensitivities. Weak storms respond primarily to thermodynamic factors, including sea-surface warming, enhanced atmospheric moisture, and increased oceanic influence along storm tracks, all of which favor prolonged inland persistence. In contrast, strong storms are more sensitive to dynamic and land-surface conditions, including faster translation speeds, increasing atmospheric stability, and drier soil, which collectively promote faster decay. Numerical experiments further demonstrate that weaker storms respond more strongly to thermodynamic enhancement, whereas stronger storms are more vulnerable to dynamic disruption. These results challenge uniform-response assumptions in tropical cyclone risk assessment, forging a dual-threat landscape: intensifying coastal impacts from faster-decaying strong storms alongside expanding inland risks from increasingly persistent weak systems. Our findings reveal pronounced intensity-dependent heterogeneity in tropical cyclones, urging refined hazard assessment and adaptation for vulnerable and unprepared regions worldwide.

## Abstract (web summary)

After landfall, strong tropical cyclones have been weakening faster in recent decades, while weak storms persist longer on land. This is associated with their different sensitivity to environmental factors under a changing climate.

## Introduction

Tropical cyclones rank among Earth’s most destructive natural hazards^1,2^, inflicting severe damage through extreme winds, storm surges, and torrential rainfall, particularly during and after landfall. As coastal populations expand and climate extremes intensify, understanding changes in tropical cyclone landfall-related hazards has become increasingly urgent^3–8^.

Prevailing scientific consensus posits that thermodynamic enhancement related to warming sea surface temperature (SST) allows tropical cyclones to retain more moisture, leading to slower post-landfall decay and broader inland rainfall over the US and China^9,10^. Recent studies reinforce this in specific contexts, such as increased residence time due to slower translation speed^11–13^ and growing inland destructiveness in East Asia and the US^14–16^.

However, emerging evidence reveals complexities: regional and temporal heterogeneity in post-landfall decay trends suggests that the thermodynamic framework alone cannot fully explain observed changes^17^, while other studies report mixed intensity changes pre-landfall^18,19^. Beyond thermodynamic forcing, tropical cyclone intensity is highly sensitive to dynamic environmental conditions. Vertical wind shear (VWS) is widely recognized as an important dynamic inhibitor of tropical cyclone intensification, acting by tilting the vortex and ventilating the warm core^20–22^. Low-level vorticity and atmospheric stability further modulate convective organization and intensity evolution of tropical cyclones^23,24^.

Tropical cyclone post-landfall decay is also highly associated with a suite of dynamic and surface factors including translation speed, soil moisture, atmospheric vorticity, and atmospheric stability^25–29^. Critically, many of these factors have undergone changes in recent decades: soil moisture has declined rapidly after 2000 driven by warming-enhanced evaporative demand^30,31^; observations and models reveal an intensified VWS at lower latitudes linked to tropical expansion and Walker circulation weakening^32,33^; VWS is also projected to increase in the future^34^. These findings suggest that simple thermodynamic explanations overlook physical interactions with key environmental factors.

Moreover, while climate change has profoundly altered tropical cyclone behavior in recent decades, including poleward migration of peak intensity^33,35^, reduced translation speeds^11,12,36^, and more frequent extremes^3,37,38^, most studies assume uniform responses across all storm types, potentially missing fundamentally distinct sensitivities that arise from structural differences across intensity categories.

Strong tropical cyclones feature deep, organized vortices with high inertial stability^39–41^, but collapse rapidly over land due to friction and energy cutoff^19,42–44^. Weak tropical cyclones, with shallower circulations and less organized convective structure, have lower energy demands and greater reliance on environmental atmospheric moisture after landfall^17,45–47^. Treating tropical cyclones homogeneously may distort post-landfall risk profiles, becuase these structural differences can govern distinct sensitivities to environmental factors.

We hypothesize that the structural differences result in divergent post-landfall decay responses to environmental factors. To test this hypothesis, we analyze 885 global landfalling tropical cyclones (1979–2024) using observations from multiple operational meteorological agencies archived in the International Best Track Archive for Climate Stewardship (IBTrACS) V4r01 dataset^48,49^. Observations reveal two interconnected changes: strong tropical cyclones now decay faster, while weak ones persist longer after landfall; concentrating coastal damage from strong storms while extending more serious inland threats from deeper-penetrating weak systems. The intensity-dependent divergent trends challenge uniform response assumptions and reshape our understanding of tropical cyclone hazard geography, necessitating differentiated adaptation strategies rather than homogeneous risk frameworks.

## Results

### Divergent post-landfall decay related to tropical cyclone intensity

**Fig. 1:**
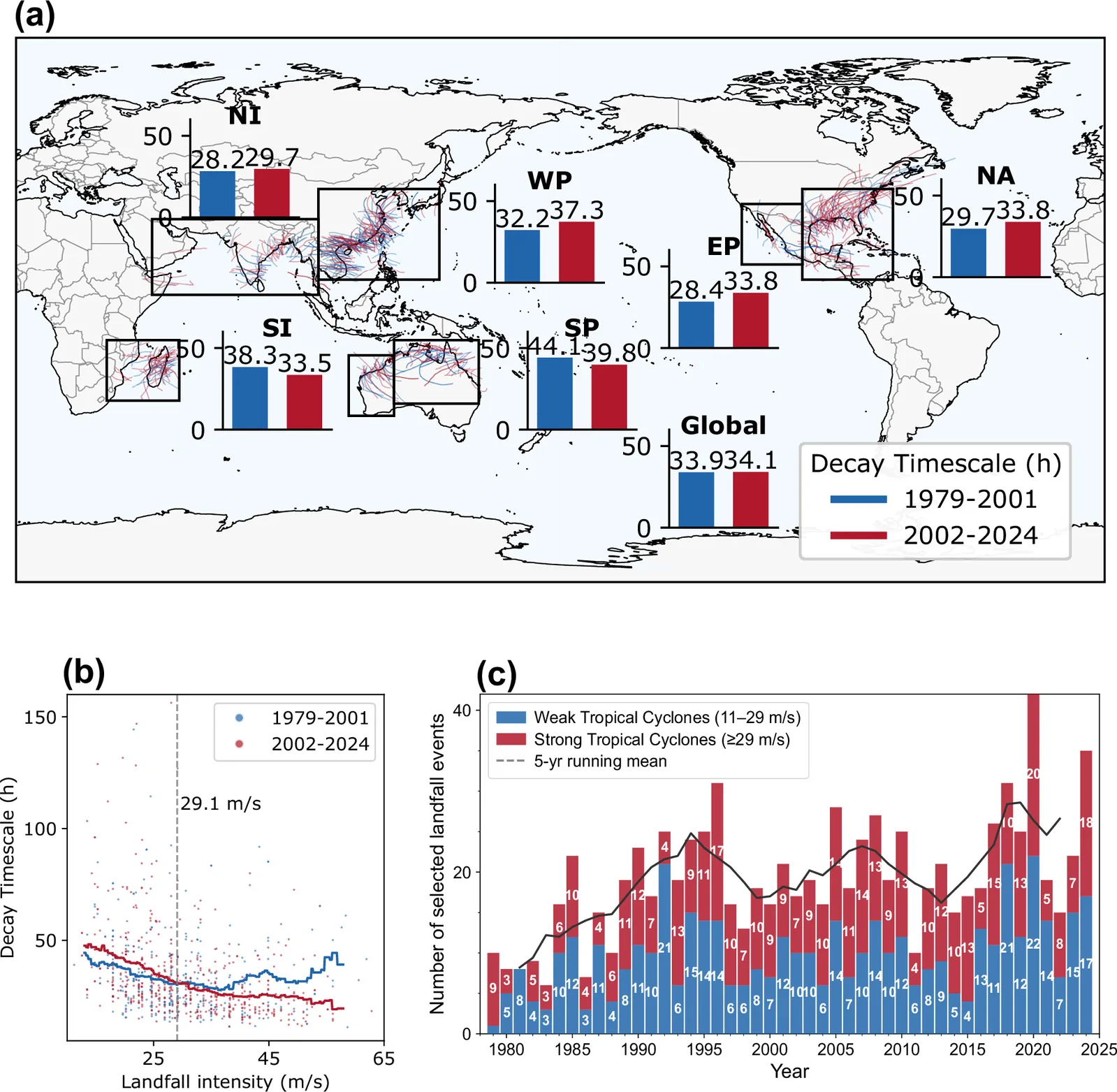
Global landfall tropical cyclone patterns and changes in decay timescale. **a** Landfall tropical cyclone tracks (1979–2024) across six major basins: Eastern Pacific (EP), North Atlantic (NA), North Indian Ocean (NI), South Indian Ocean (SI), South Pacific (SP), and Western Pacific (WP), colored by early (blue, 1979–2001) and late (red, 2002–2024) periods. Bar charts show global and basin-specific mean decay timescale (h). **b** Relationship between landfall intensity and decay timescale for global landfall tropical cyclones; each dot represents an individual landfall event. Blue and red lines show the running mean of decay timescale across landfall intensity bins during the early and late periods; dashed vertical line marks the intensity threshold (29.1 m/s) at which the difference in decay timescale between the two periods reverses sign. **c** Annual time series of number of selected landfall events. Red and blue bars indicate annual number of strong (≥ 29 m/s) and weak tropical cyclones, with event counts shown as white numbers. Black curve shows the 5-year running mean of total events.

We selected 885 global landfalling tropical cyclones across six basins for the period 1979–2024 (see “Methods”). Figure 1a shows the tracks of all selected events. For each event, we quantified the post-landfall decay rate using an exponential model fitted to the maximum sustained wind time series, extracting the decay timescale (τ)^9,10,50,51^, which represents the time for storm intensity to decrease to 1/*e* of its landfall intensity; larger τ indicates slower decay. During recent decades, global mean decay timescale shows a modest increase between the early (1979–2001; 33.9 h, 95% confidence interval [CI]: 31.5–36.2 h) and late (2002–2024; 34.1 h, 95% CI: 31.8–36.7 h) periods, especially in Western Pacific (WP, 32.2 versus 37.3 h, 95% CI: 29.2–35.8 versus 32.4–44.0) and North Atlantic (NA, 29.7 versus 33.8 h, 95% CI: 24.50–35.55 versus 28.32–40.06), consistent with prevailing findings that warming prolongs tropical cyclone inland persistence^9,10^. However, South Indian Ocean (SI) and South Pacific (SP) show declines, suggesting regional heterogeneity beneath the global signal.

This aggregate view conceals an intensity-dependent bifurcation. Decay timescale decreases exponentially with landfall intensity (Supplementary Fig. 1a), reflecting the physical principle that intense storms decay faster than weak ones^50,52^. Yet this relationship has undergone a substantial shift in recent decades (Fig. 1b): compared to the early period (1979–2001), global weak tropical cyclones in the late period (2002–2024) exhibit larger decay timescales, indicating slower inland decay, whereas strong tropical cyclones demonstrate smaller decay timescales, resulting in opposing changes that rotate the intensity–decay relationship clockwise. The transition occurs at a landfall intensity of 29.1 m/s (10-min maximum wind speed; 95% CI: 27.6–30.1 m/s). This bifurcation is consistent across most basins (Supplementary Fig. 1b), particularly in WP and NA, with basin-level thresholds ranging from 25 to 34 m/s. We therefore adopt 29 m/s as the global classification threshold, yielding 429 strong and 456 weak tropical cyclones.

The annual counts of selected tropical cyclones show an increasing trend during 1979–2024 (Fig. 1c). However, this should not be interpreted as evidence of an increase in global landfalling frequency, as the trend likely reflects improvements in observational coverage and data quality over time. Notably, storm counts during the early satellite era (1979–1984) are substantially lower than in subsequent years.

**Fig. 2:**
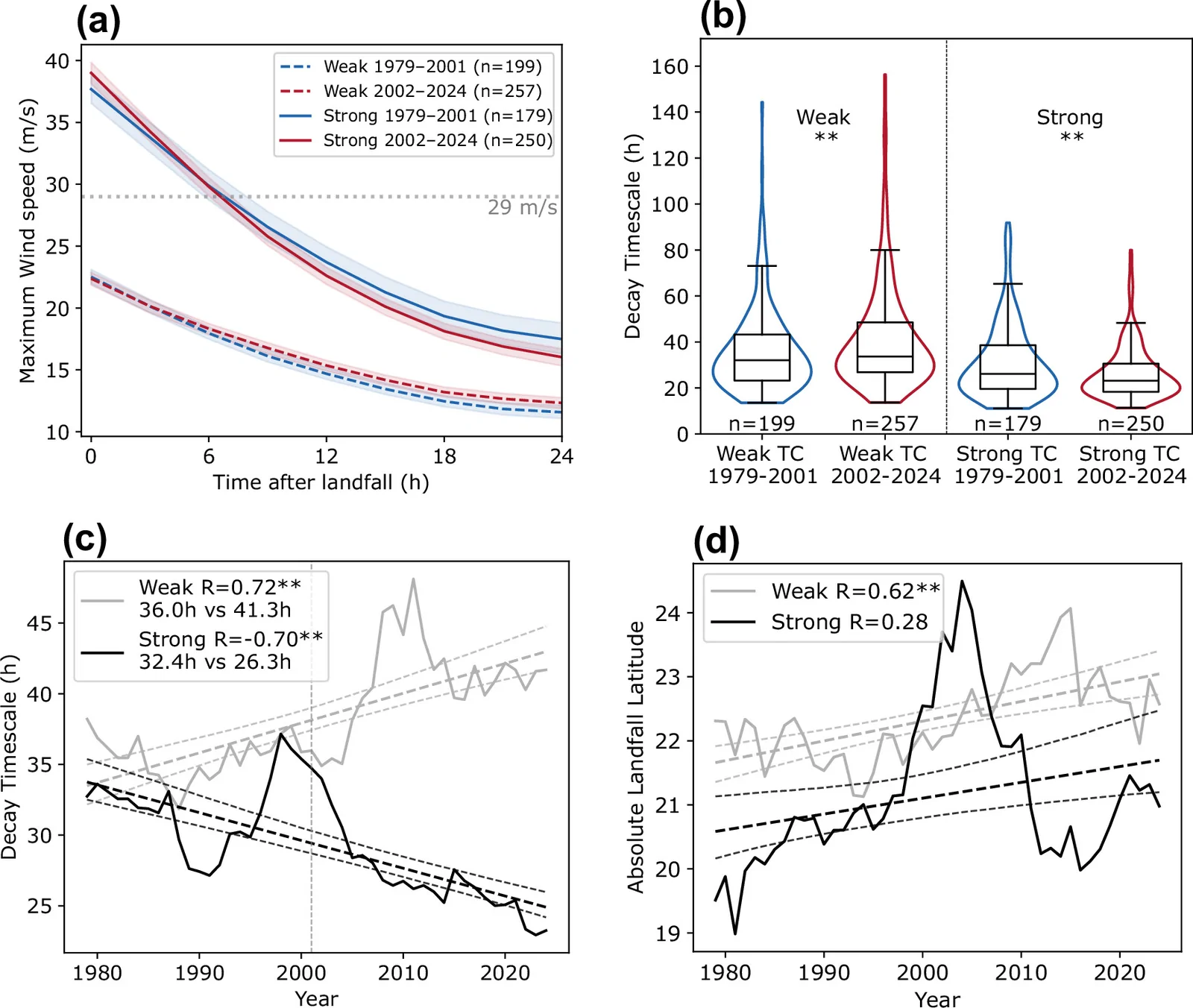
Intensity-dependent changes in tropical cyclone post-landfall decay from 1979–2024. **a** Time series of post-landfall wind speed for strong (solid line) and weak (dashed line) tropical cyclones, showing early (blue, 1979–2001) versus late (red, 2002–2024) period means; shading in corresponding color denotes the 95% bootstrap confidence interval (CI; *n* = 10,000 resamples). Gray dashed line indicates the 29 m/s threshold between strong and weak storms. **b** Distribution of decay timescales across intensity categories and periods. Violin plots show kernel density estimates of the full distribution; overlaid box plots indicate the median (center line), interquartile range (box limits), and 1.5× interquartile range (whiskers); outliers are not shown. Sample sizes are indicated as “*n*”. Asterisks denote significant differences between periods (** *P* < 0.01). Temporal trends in **c** decay timescale and **d** absolute landfall latitude indicating poleward migration patterns, for strong and weak tropical cyclones using 7-year running means. Dashed lines show linear regression fits; outer dashed lines indicate the 95% bootstrap CI. Correlation coefficients (*R*) and significance levels are annotated; vertical dashed line marks the period boundary (2001).

Post-landfall wind speed time series reveal the physical basis for intensity-dependent decay changes (Fig. 2a). Maximum sustained wind speeds follow exponential decay after landfall, with broadly similar mean patterns between periods. However, a divergence emerges: despite higher landfall intensities in the late period (2002–2024), strong tropical cyclones decay more rapidly, falling below early-period (1979–2001) wind speeds within approximately 6 h after landfall. In contrast, weak tropical cyclones maintain higher wind speeds for longer durations in the late period.

Distribution analysis confirms these divergent changes (Fig. 2b). Weak tropical cyclones increasingly produce long-lasting events, with mean decay timescale rising 14.5% between the early (36.0 h, 95% CI: 33.4–38.7 h) and late (41.3 h, 95% CI: 38.4–44.1 h) periods (*P* < 0.01). Strong cyclones show compressed distributions, with mean decay timescale declining 18.6% from 32.4 h (95% CI: 29.2–35.6 h) to 26.3 h (95% CI: 24.9–27.9 h) (*P* < 0.01). This bifurcation is consistent across most basins (Supplementary Fig. 2), except for SI and SP, where both strong and weak storms exhibit decreased decay timescales.

Temporal trend analysis reveals opposing long-term changes in strong and weak tropical cyclone decay (Fig. 2c). Weak tropical cyclones show an overall increasing trend in decay timescale (0.21 h/yr, 95% CI: 0.15–0.27, *R* = 0.72, *P* < 0.01), while strong tropical cyclones exhibit a decreasing trend (−0.20 h/yr, 95% CI: −0.26 to −0.14, *R* = −0.70, *P* < 0.01). However, these trends are accompanied by substantial interannual to interdecadal variability: weak tropical cyclone decay timescale remained relatively stable from 1979 to approximately 2000 before rising sharply, then declined gradually after 2012; strong tropical cyclone decay timescale fluctuated considerably through the mid-1990s before entering a pronounced and sustained decline. Such variability is expected given the limited annual sample sizes and the complex interaction between environmental factors and post-landfall decay, consistent with previous studies^9,10^. Natural variability contributes substantially to these fluctuations; many peak values coincide with La Niña years (Supplementary Fig. 3a), and weak tropical cyclone decay timescales are significantly larger during La Niña years compared to El Niño and neutral years (Supplementary Fig. 3b, c), with a similar but weaker pattern for strong tropical cyclones. Nevertheless, the intensity-dependent bifurcation is robust, especially in the post-2000 period.

The robustness is further supported by multiple lines of evidence. Analysis of the direct decay rate (wind speed loss within 24 h of landfall) yields consistent results (Supplementary Fig. 4). Weak tropical cyclones show a decreasing decay rate trend (−0.03 m/s/yr, 95% CI: −0.04 to −0.02, *R* = −0.57, *P* < 0.01), while strong tropical cyclones show an increasing trend (0.09 m/s/yr, 95% CI: 0.06–0.12, *R* = 0.68, *P* < 0.01), with corresponding period differences (weak: 10.7 vs. 10.0 m/s; strong: 20.9 vs. 23.4 m/s; threshold = 28.6 m/s) corroborating the decay timescale results. Moreover, sensitivity tests using alternative intensity thresholds and temporal splits reproduce the intensity-dependent patterns (Supplementary Fig. 5).

In addition, analysis of 1,014 synthetic North Atlantic tropical cyclones from the Risk Analysis Framework for Tropical Cyclones (RAFT) framework^53^ also reveals a significant increase in weak tropical cyclone decay timescales and a significant decrease for strong tropical cyclones (threshold = 29.4 m/s), lending independent support to our observational findings (Supplementary Fig. 6).

Landfall patterns also show intensity-dependent changes. Weak tropical cyclones exhibit a significant poleward shift (0.03°/yr, 95% CI: 0.02–0.04, *R* = 0.62, *P* < 0.01), consistent with documented poleward migration of tropical cyclone activity^33,35^. Strong tropical cyclones show a more complex change: a poleward shift during 1979–2005 gave way to a pronounced equatorward retreat in recent decades, resulting in an overall positive trend (0.025°/yr, 95% CI: 0–0.05, *P* = 0.06). These contrasting latitudinal evolutions, especially after 2000, suggest potentially distinct evolution in environmental landfall regimes.

### Changing hazard geography: coastal intensification and inland penetration

**Fig. 3:**
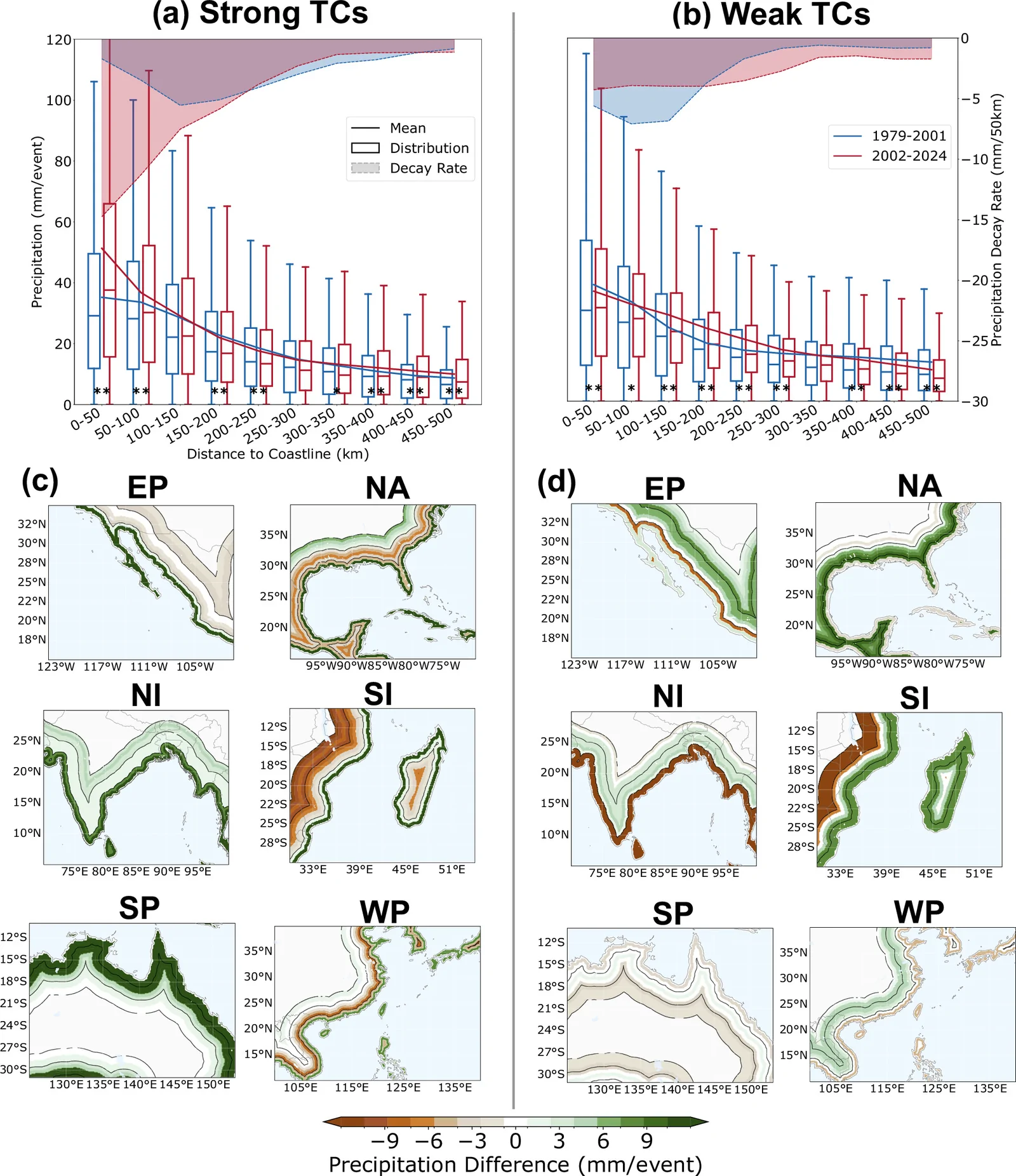
Landfall tropical cyclone (TC) precipitation patterns and coast-to-inland changes. Precipitation accumulated over the first 24 h after landfall as a function of distance to coastline for **a** strong and **b** weak tropical cyclones. Lines show mean precipitation for each 50-km distance bin; box plots indicate the median (center line), interquartile range (box limits), and 1.5× interquartile range (whiskers); outliers are not shown. Blue and red colors correspond to the early (1979–2001) and late (2002–2024) periods, respectively. Shaded areas on the secondary axis show the rate of precipitation decay with increasing distance from the coastline (mm/50 km). Asterisks denote statistically significant differences in precipitation between periods (* *P* < 0.05, ** *P* < 0.01). **c**, **d** Spatial changes in landfall-associated precipitation (2002–2024 minus 1979–2001) within a 500-km coastal band (50-km intervals) across six basins: Eastern Pacific (EP), North Atlantic (NA), North Indian Ocean (NI), South Indian Ocean (SI), South Pacific (SP), and Western Pacific (WP). Color shading represents area-averaged precipitation differences within each distance zone; green indicates increased precipitation and brown indicates decreased precipitation.

The intensity-dependent changes in post-landfall decay are reshaping global hazard geography, creating a dual-threat scenario: strong tropical cyclones increasingly concentrate damage near coastlines, while weak ones extend impacts further inland (Fig. 3).

Tropical cyclones exhibit decreasing landfall terrestrial precipitation (24 h after landfall) with increasing coastal distance. Compared to the early period (1979–2001), strong tropical cyclones now exhibit pronounced coastal concentration (Fig. 3a, c), with accumulated precipitation intensifying by 45.6% (16.1 mm/event, *P* < 0.01) within 0–50 km of coastlines and by 9.3% (3.1 mm/event, *P* < 0.01) within 50–100 km, but this enhancement diminishes rapidly beyond 150 km, where period differences become negligible or even negative. The rate of precipitation decay with inland distance steepens correspondingly in the late period, directly reflecting the accelerated post-landfall decay of strong storms. This coastal concentration pattern appears across most major basins (Supplementary Table 1), with the largest increases in NA (+51.4%) and SI (+50.4%) basins.

Weak tropical cyclones show opposite changes through enhanced inland persistence (Fig. 3b, d). Despite reduced coastal precipitation (0–50 km, −5.6%, *P* < 0.01), late-period weak tropical cyclones deliver stronger precipitation to inland areas: precipitation rises by 25.5% at 150–200 km and 21.7% at 200–250 km from the coastline (*P* < 0.01). This inland penetration effect is most pronounced in Eastern Pacific (EP, +26.6%) and WP (+11.5%) basins (Supplementary Table 1), consistent with the longer decay timescales observed for weak storms in these regions.

Energy dissipation patterns measured by the power dissipation index (PDI)^1^ corroborate these spatial redistributions (Supplementary Fig. 7). Strong cyclones show markedly accelerated energy release within 150 km from coastlines, while weak cyclones maintain lower PDI decay rates throughout inland progression, confirming the substantial differences in post-landfall hazard geography changes across intensity categories.

### Distinct environmental sensitivities and contributions to divergent decay timescale trends

To identify environmental controls underlying these contrasting decay changes, we evaluated key factors across three physically meaningful categories (Supplementary Table 2): tropical cyclone characteristics (landfall intensity, translation speed, ocean area ratio [OAR], topographic height) that govern initial energy, surface friction, and potential energy replenishment^17,26,54,55^; thermodynamic features (temperature and humidity) that supply energy for storm maintenance^9,15,29,46,47,56^; and dynamic features (atmospheric vorticity and stability) that modulate storm organization^21–25,28^.

Fundamentally different sensitivities emerge between strong and weak tropical cyclones (Table 1). For strong storms, decay timescale is significantly correlated with translation speed (event-scale *R* = −0.33, year-scale *R* = −0.75, *P* < 0.01), soil moisture anomaly (*R* = 0.19, *R* = 0.74, *P* < 0.01; stronger tropical cyclones have a lower inflow boundary layer^57^), and all dynamic factors including 850 hPa vorticity (*R* = 0.26, *R* = 0.62, *P* < 0.01), 500 hPa vertical wind speed (*R* = 0.11, *R* = 0.43, *P* < 0.05), and VWS (*R* = −0.13, *R* = −0.53, *P* < 0.01), indicating that dynamics and land-surface factors are robustly associated with strong storm decay. In contrast, weak tropical cyclone decay timescale shows significant correlations with SST anomaly (*R* = 0.15, *R* = 0.71, *P* < 0.01), OAR (*R* = 0.35, *R* = 0.66, *P* < 0.01), and 850 hPa specific humidity anomaly (*R* = 0.13, *R* = 0.45, *P* < 0.01), but no significant correlations with any of the dynamic factors.

**Table 1:** Correlation coefficients between tropical cyclone decay timescale and key environmental factors

Key factors	Strong TCs		Weak TCs	
	Event-scale correlation	Year-scale correlation	Event-scale correlation	Year-scale correlation
Landfall intensity (m/s)	0.06	−0.12	0.24^**^	−0.13
Translation speed (km/h)	−0.33^**^	−0.75^**^	−0.04	−0.30
Ocean area ratio	0.39^**^	0.29	0.35^**^	0.66^**^
Topographic height (m)	−0.13^**^	0.03	−0.15^**^	0.13
Sea surface temperature anomaly (K)	−0.13^**^	−0.61^**^	0.15^**^	0.71^**^
850 hPa specific humidity anomaly (g/kg)	0.17^**^	0.29	0.13^**^	0.45^**^
Soil moisture anomaly (m^3^/m^3^)	0.19^**^	0.74^**^	0.01	−0.44^**^
850 hPa Vorticity (1/s)	0.26^**^	0.62^**^	0.05	−0.15
500 hPa Vertical wind speed (m/s)	0.11^*^	0.43^**^	0.04	0.21
**Vertical wind shear (m/s)**	−0.13^**^	−0.53^**^	0.03	0.05

Event-scale and year-scale correlation between decay timescale for strong and weak tropical cyclones and key environmental factors. Asterisks indicate statistical significance levels (*P < 0.05; **P < 0.01).

These contrasting sensitivities are consistent with structural differences: strong cyclones remain extremely vulnerable to unfavorable dynamic disruptions (faster speed, more stable atmosphere), their high energy demands cannot be sustained by terrestrial moisture sources after landfall; weak cyclones with less organized convective structure and less energy demand have modest energy requirements and greater reliance on atmospheric moisture.

Notably, strong tropical cyclone decay timescale exhibits a significant negative correlation with SST (*R* = −0.13, *R* = −0.61, *P* < 0.01). This apparent reversal reflects a potential spurious temporal correlation^58^ driven by the concurrent significant warming trend and the decreasing trend in decay timescale of strong storms; as demonstrated by numerical model experiments shown in the next section, SST warming does in fact slow decay for strong tropical cyclones.

**Fig. 4:**
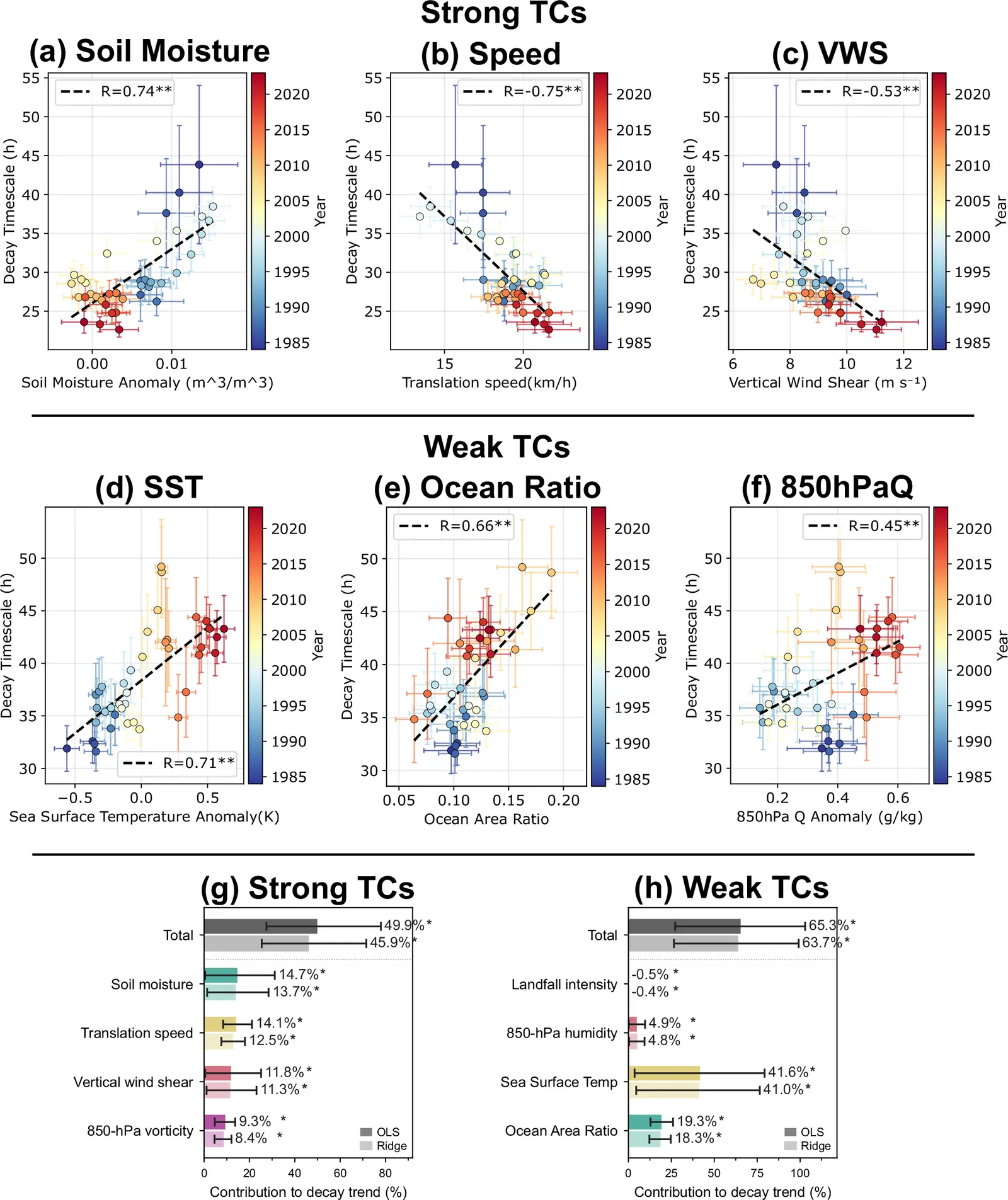
Relationships between key environmental factors and tropical cyclone (TC) decay timescale, and their contributions to the observed trends. Scatter plots between annual-mean decay timescale and **a** soil moisture anomaly, **b** translation speed, **c** vertical wind shear (VWS), **d** sea surface temperature (SST) anomaly, **e** ocean area ratio (OAR), and **f** 850-hPa specific humidity anomaly, for strong (**a**–**c**) and weak (**d**–**f**) tropical cyclones, with dot color indicating year and error bars denoting ±1 standard error of 5-year window. The dashed line shows the ordinary least squares (OLS) regression fit, with the Pearson correlation coefficient (*R*) and significance level indicated (* *P* < 0.05, ** *P* < 0.01). Estimated contributions of each environmental variable to the observed long-term trend in decay timescale for **g** strong and **h** weak tropical cyclones. Dark and light bars represent OLS and Ridge regression estimates, respectively, with error bars denoting 95% bootstrap confidence intervals (CI). Asterisks denote significant contributions (* *P* < 0.05).

These distinct sensitivities provide a physically grounded framework for interpreting the observed divergent decay trends (Fig. 4a–f). For strong tropical cyclones, three factors emerge as most closely associated with, and physically consistent with, the accelerating decay. First, soil moisture anomaly at landfall locations has declined significantly, with an especially abrupt decline since 2000, and has remained at low levels in recent decades due to warming-enhanced evaporative demand^30,31^ (−0.0003 m^3^/m^3^/yr, *R* = −0.67, *P* < 0.01; Supplementary Fig. 8a). This drying trend co-varies closely with annual decay timescale (*R* = 0.74, *P* < 0.01; Fig. 4a). Soil moisture in tropical cyclone season shows a substantial drying trend in most basins over the past 25 years (Supplementary Fig. 9a). Second, post-landfall translation speed has increased significantly though with interannual fluctuation (0.07 m/s/yr, *R* = 0.54, *P* < 0.01; Supplementary Fig. 8b), associated with the enhanced steering flow under global warming^59,60^. Annual decay timescale tracks this acceleration closely (*R* = −0.75, *P* < 0.01; Fig. 4b), with almost opposed trends. Third, annual decay timescale declines in concert with increasing VWS (*R* = −0.53, *P* < 0.01; Fig. 4c, Supplementary Fig. 8c). VWS has intensified across most basins in subtropical regions during recent decades (Supplementary Fig. 9b), especially in South China and Southern US, as global warming has been shown to preferentially increase atmospheric stability at lower latitudes linked to amplified upper-tropospheric warming and tropical expansion^32,33,38^. The declining 850 hPa vorticity and 500 hPa upward motion further confirm that the faster post-landfall decay matches well with the more stable atmosphere, though with strong interannual variability (*R* = 0.62 and *R* = 0.43, both *P* < 0.01; Supplementary Fig. 10). Notably, the co-occurrence of low translation speed, high soil moisture, weak VWS, and high vorticity and vertical motion around 1998 coincides precisely with the peak in strong storm decay timescale (Fig. 2c), consistent with an extended La Niña period that prevailed during 1996–2001.

For weak tropical cyclones, three thermodynamic factors underpin the observed increase in decay timescale. SST anomaly has warmed significantly across all basins (0.03 K/yr, *R* = 0.98, *P* < 0.01; Supplementary Fig. 8d; Supplementary Fig. 9c), and annual decay timescale co-varies significantly with this warming trend (*R* = 0.71, *P* < 0.01; Fig. 4d), consistent with SST’s role in sustaining moisture flux^9^. OAR for weak tropical cyclones has also increased over time (0.1%/yr, *R* = 0.39, *P* < 0.01; Supplementary Fig. 8e), with significant correlation with annual decay timescale (*R* = 0.66, *P* < 0.01; Fig. 4e). Notably, the trends in OAR and landfall latitude are highly consistent; a poleward shift may alter the landfall angle of storms and enhance OAR^9,17^. Additionally, 850 hPa specific humidity has increased significantly in the Northern Hemisphere basins (WP, EP, NA, NI; Supplementary Fig. 9d). Its year-scale correlation with decay timescale confirms the role of enhanced atmospheric moisture in weak storm maintenance (*R* = 0.45, *P* < 0.01; Fig. 4f, Supplementary Fig. 8f). Importantly, the Southern Hemisphere basins (SP, SI) show drying trends (Supplementary Fig. 9d), consistent with the observed reduction in precipitation and atmospheric moisture^61,62^. This regional heterogeneity is associated with faster decay of all storms in SP and SI (Supplementary Fig. 2).

Multivariate trend decomposition quantifies the fractional contribution of key factors to the observed long-term decay trends (Fig. 4g, h; see “Methods”). For strong tropical cyclones, soil drying, accelerated translation speed, VWS enhancement, and vorticity reduction together contribute to 45.9%–49.9% of the observed decreasing trend in decay timescale. Rrobustness is confirmed by broadly consistent estimates from ordinary least squares (OLS) and Ridge regression. For weak tropical cyclones, SST warming emerges as the dominant contributor, with OAR increase and humidity enhancement providing secondary contributions, collectively contributing 63.7%–65.3% of the trend. Together, these results converge on a coherent picture: the divergent changes in strong and weak tropical cyclone decay are related to their distinct environmental sensitivities.

Moreover, the observed divergent decay trends might reflect changes in storm intensity itself. However, the year-scale correlation between landfall intensity and decay timescale is insignificant for both strong (*R* = −0.12) and weak storms (*R* = −0.13; Supplementary Fig. 11a, b), and the temporal evolution of landfall intensity is inconsistent with that of decay timescale for both intensity categories (Supplementary Fig. 11c). These mismatches indicate that changes in landfall intensity alone cannot explain the observed divergent decay trends.

### Numerical model evidence for intensity-dependent environmental sensitivities

To provide process-level evidence for the intensity-dependent environmental sensitivities, we conducted idealized simulations using the Cloud Model 1 (CM1)^63–65^, in which tropical cyclones of prescribed landfall intensities make landfall with the enthalpy exchange coefficient set to zero^9,43,44,52^ (see “Methods”). The sensitivity of post-landfall decay to VWS and SST was examined across four landfall intensity groups.

**Fig. 5:**
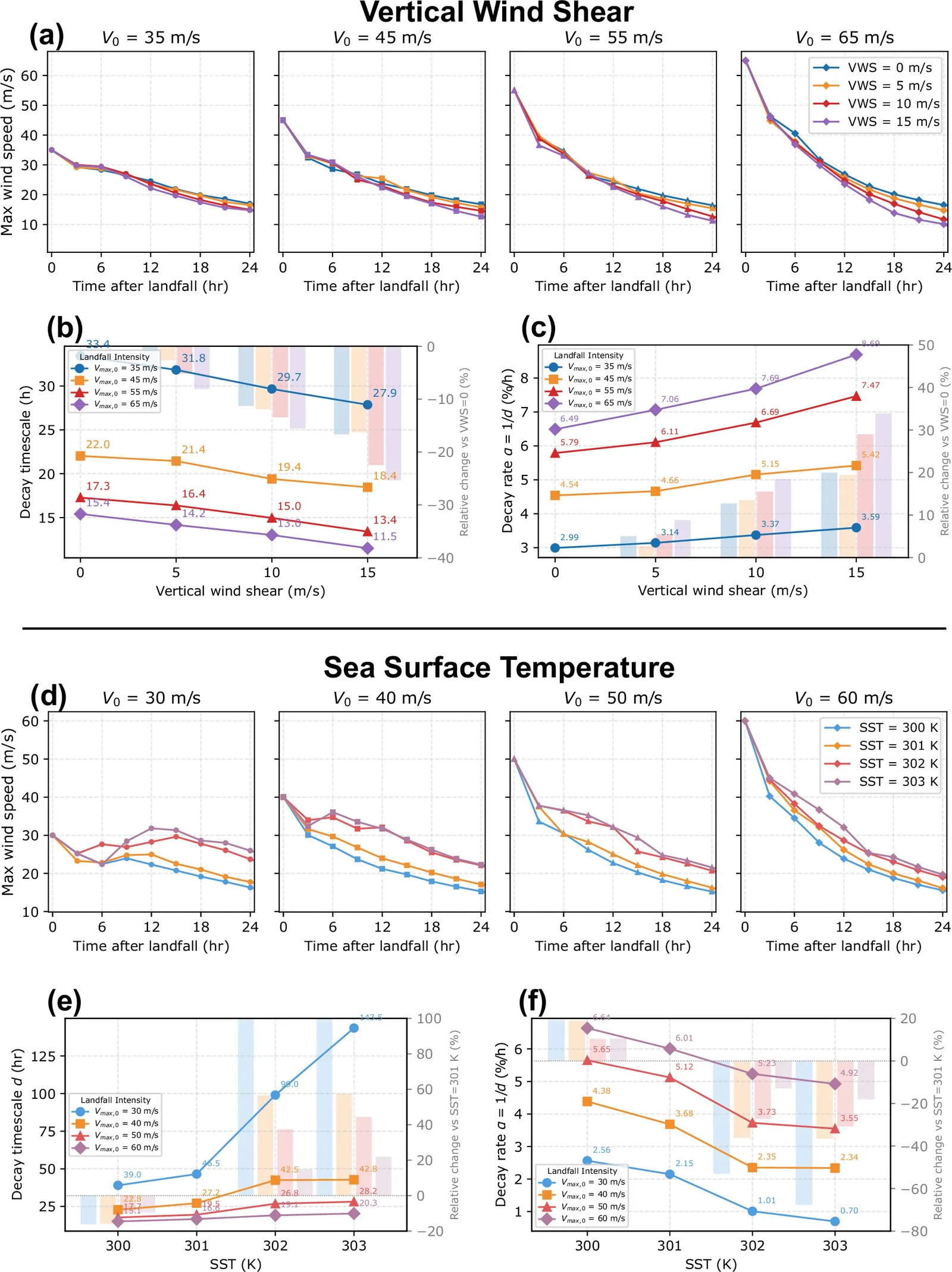
Post-landfall decay sensitivity to vertical wind shear and sea surface temperature in idealized Cloud Model 1 (CM1) simulations. **a** Time series of maximum 10-m wind speed for tropical cyclones with landfall intensities of 35, 45, 55, and 65 m/s under four vertical wind shear (VWS) environments (VWS = 0, 5, 10, and 15 m/s), with sea surface temperature (SST) fixed at 301 K. **b** Decay timescale *d* (left axis) and its relative change with respect to the VWS = 0 experiment (right axis, bars) as a function of VWS, for each landfall intensity group. **c** As in (**b**), but for the decay rate *α* = *1/d*. **d** As in (**a**), but for tropical cyclones with landfall intensities of 30, 40, 50, and 60 m/s under four SST conditions (SST = 300, 301, 302, and 303 K), with VWS set to zero. **e** As in (**b**) but as a function of SST, with relative changes computed with respect to the SST = 301 K experiment. **f** As in (**e**), but for the decay rate.

Post-landfall maximum sustained wind speed time series confirm that stronger tropical cyclones decay faster and respond more strongly to VWS (Fig. 5a). Without VWS, decay timescale ranges from 33.4 h at landfall intensity *V*_0_ = 35 m/s to 15.4 h at *V*_0_ = 65 m/s (Fig. 5b), consistent in magnitude with observed values (Fig. 1b). As environmental VWS increases, decay timescale decreases across all intensity groups, but the magnitude of the response scales strongly with landfall intensity: at VWS = 15 m/s, the decay timescale of the storm with *V*_0_ = 65 m/s decreases by more than 25% relative to the no-shear control (from 15.4 to 11.5 h), whereas the storm with *V*_0_ = 35 m/s decreases by 16% (from 33.4 to 27.9 h). This intensity-dependent VWS sensitivity is further confirmed by the decay rate metric (35% faster decay for *V*_0_ = 65 m/s compared to 20% for *V*_0_ = 35 m/s; Fig. 5c).

The physical basis for this differential VWS sensitivity is related to structural differences. At landfall, stronger tropical cyclones exhibit higher wind speed and smaller radii of maximum wind (RMW; 18 km for *V*_0_ = 65 m/s versus 30 km for *V*_0_ = 35 m/s; Supplementary Fig. 12a), reflecting more compact and energetically intense vortex structures with much greater frictional dissipation upon landfall. Vertical profiles of potential temperature anomaly (core minus environment) reveal that weaker storms possess shallower and weaker warm-core structures, whereas stronger storms develop deeper and more pronounced warm cores with the maximum ascending to approximately 14 km (Supplementary Fig. 12b). These structural contrasts directly determine the response to VWS: at *t* = 12 h, the vortex tilt induced by VWS relative to the no-shear control is substantially larger for stronger storms (Supplementary Fig. 12c). The corresponding potential temperature anomaly difference profiles (Supplementary Fig. 12d) further clarify that, for the weaker storm, VWS produces only modest warming reduction; for the *V*_0_ = 65 m/s storm, VWS drives a pronounced upper-level warming deficit alongside a low-level apparent warming that directly reflects vortex asymmetry and structural disruption of the organized circulation.

The spatial evolution of post-landfall wind fields corroborates this structural breakdown. For *V*_0_ = 35 m/s under VWS = 0, the vortex maintains a coherent, albeit weakening, symmetric circulation through 24 h after landfall (Supplementary Fig. 13a). Under VWS = 15 m/s, asymmetry develops progressively, with wind speed reductions on the northern semicircle and enhancement to the south, but an unorganized vortex structure persists. In contrast, the *V*_0_ = 65 m/s storm under VWS = 0 exhibits a compact and intense core (Supplementary Fig. 13b), but under VWS = 15 m/s the circulation becomes strongly asymmetric by *t* = 6 h, is substantially disrupted by *t* = 12 h, and retains almost no coherent vortex structure by *t* = 18–24 h with strongly reduced wind speed. These results demonstrate that strong tropical cyclones are more sensitive to VWS-induced structural disruption, experiencing much faster decay in high-VWS environments.

On the other hand, SST warming slows post-landfall decay across all intensity groups, but the magnitude of the enhancement diminishes strongly with landfall intensity (Fig. 5d). For weaker storms (*V*_0_ = 30 m/s) at SST = 303 K, the storm even undergoes a brief re-intensification after *t* = 6 h, likely reflecting latent heat release from enhanced moisture. The decay timescale increases nearly threefold from 46.5 h at SST = 301 K to 142.5 h at SST = 303 K (Fig. 5e). In contrast, strong tropical cyclones show a weaker response: for *V*_0_ = 60 m/s, the decay timescale increases from 16.6 h to 20.3 h (22%). The decay rate (Fig. 5f) corroborates this intensity-dependent sensitivity. Although the idealized experimental setup without realistic terrain and with simplified surface drag and fixed SST may not perfectly replicate observed decay, the differential SST sensitivity is robust.

The physical mechanism underlying this intensity-dependent SST sensitivity is illuminated by the energy ratio diagnostic *S*(*t*) (Supplementary Fig. 14a, see “Methods”). *S*(*t*) is defined as the ratio of SST-warming-induced column-integrated moist static energy enhancement (*H*_sst303_ − *H*_sst300_) to the cumulative surface frictional dissipation of the SST = 300 K experiment (*E*_fric-sst300_). *S*(*t*) decreases monotonically with landfall intensity, while *S* for stronger storms is substantially lower, especially during the first 6 h after landfall. This reflects the fundamental energy budget asymmetry that strong storms sustain much larger wind speeds and correspondingly greater frictional dissipation rates (*ε* ∝ |*V*|^3^), such that the incremental energy gain from SST warming and a more humid atmosphere relative to the energy consumption is less important. Weak storms with lower dissipation rates retain a larger fraction of the SST-induced energy surplus, enabling sustained intensity maintenance after landfall.

Vertical profiles of potential temperature anomaly at *t* = 12 h provide further structural evidence. For the storm at *V*_0_ = 30 m/s, progressively warmer SST produces stronger warm-core anomalies throughout the lower and middle troposphere (Supplementary Fig. 14b), consistent with enhanced moisture and latent heat release sustaining the thermal structure of the shallow vortex. For the stronger storm (*V*_0_ = 60 m/s), the warm-core differences between SST experiments are confined to 8–14 km altitude and of smaller relative magnitude (Supplementary Fig. 14c). These thermal responses confirm that weak tropical cyclone decay is more sensitive to SST warming and enhanced environmental moisture, corroborating the observational findings.

## Discussion

Our findings reveal intensity-dependent bifurcation in global tropical cyclone post-landfall decay trends that are overlooked in aggregate regional analyses. Strong tropical cyclones now weaken 18.6% faster after landfall, while weak ones persist 14.5% longer inland over the 1979–2024 period. This bifurcation challenges the assumption that global warming uniformly prolongs tropical cyclone inland persistence, and underscores that intensity-stratified analysis is essential for characterizing how tropical cyclone hazards evolve under a changing climate.

Reanalysis diagnostics and numerical model experiments consistently demonstrate that strong and weak tropical cyclones possess distinct sensitivities to environmental factors. Strong cyclones possess deep and organized vortices^39–41^ with intense energy demands^42–44^ that are highly sensitive to dynamic disruption. Weak cyclones’ shallow, loosely organized structure and lower energy demand^45–47^ enable greater thermodynamic sensitivity. Accelerating translation speeds and increasing atmospheric stability that are directly linked to dynamic disruption, together with a trend toward drier soils, are physically associated with the accelerated decay of global strong tropical cyclones in recent decades. Although SST warming acts to slow the decay of strong tropical cyclones, this thermodynamic benefit is overwhelmed by the unfavorable dynamic and land-surface forcings. In contrast, SST warming and enhanced atmospheric moisture are strongly associated with weak cyclones’ prolonged inland persistence.

CMIP projections indicate that several key environmental drivers may persist: soil moisture faces continued decline^66,67^; faster-moving landfalling tropical cyclones due to enhanced steering flow^59^; higher tropical atmospheric stability linked to amplified upper-tropospheric warming and Walker circulation weakening and tropical expansion^32,34^. The environment of strong landfalling storms may become increasingly unfavorable, while enhanced temperature and moisture will sustain weak storms' persistence, suggesting that the intensity-dependent bifurcation may persist or even amplify in the future.

Importantly, our results indicate that basin-aggregated analyses can obscure intensity-dependent patterns, potentially distorting risk projections. Aggregate decay timescales increase in WP and NA, yet this masks divergent changes across intensity categories. The Southern Hemisphere basins (SP and SI) even show faster decay for all storms. More broadly, assuming uniform thermodynamic responses misattributes drivers and misses the dynamically associated acceleration of strong storm decay. These findings align with the recognition in IPCC AR6^68^ that extreme weather response to changing climate is highly heterogeneous. The intensity-dependent paradigm likely extends to other extreme weather events where structural differences across categories govern distinct climate responses, suggesting that uniform-response assumptions may misrepresent future hazard profiles.

The associated dual-threat scenario demands comprehensive reassessment of disaster preparedness frameworks. Faster-decaying strong tropical cyclones concentrate damage near coastlines, intensifying compound risks for coastal megacities stressed by sea-level rise and enhanced extreme precipitation^6,69^. Increasingly persistent weak systems extend inland threats to unprepared interior communities with limited adaptive capacity. This shifting hazard geography necessitates updating existing risk models and emergency protocols with intensity-stratified frameworks for vulnerable and unprepared regions worldwide.

Several limitations warrant acknowledgment. The 1979–2024 analysis incorporates observations from multiple operational agencies to enhance robustness, but early-period data carry uncertainties arising from limited observational coverage and data quality issues, particularly for weak tropical cyclones. Although pre-2000 trends exhibit greater interannual variability partly related to sparser observational coverage and natural variability, the divergence in intensity-dependent trends strengthens markedly after 2000, coinciding with the period of rapid climate change and improved data quality. The idealized CM1 simulations, while valuable for isolating the sensitivity of post-landfall decay to VWS and SST, employ fixed SST, no background environmental flow, and simplified surface conditions; real-world interactions such as soil moisture gradients, coastal geometry, and land surface heterogeneity may further modulate the intensity-dependent responses. The trend decomposition framework retains the four most physically grounded predictors to minimize multicollinearity, accounting for 46%–65% of trend variance. The residual variance reflects contributions from other environmental factors not included, nonlinear interactions, and large-scale circulation variability and basin-specific teleconnections (e.g., El Niño-Southern Oscillation [ENSO], Atlantic Multidecadal Oscillation [AMO]). The ENSO modulation of decay timescale (Supplementary Fig. 3) suggests a potential contribution, though it does not negate the long-term trend signal. These observed long-term trends likely reflect a superposition of anthropogenic forcing and natural variability. Nevertheless, the significant trends in key hydroclimate factors over the recent 25 years, closely tracking the divergent decay timescale trends, potentially suggest climate change signal as an important contributor. Future work extending this framework to large-ensemble high-resolution CMIP6 experiments will be essential for formal attribution and for assessing whether the intensity-dependent bifurcation amplifies under different scenarios.

## Methods

### Data description

Tropical cyclone observations (maximum sustained wind and position at 3-hourly intervals) were obtained from the IBTrACS V4r01 dataset^48,49^. To build a robust, globally consistent dataset, we incorporated data from multiple agencies across six ocean basins: the US best-track agencies, including the National Hurricane Center (NHC; HURDAT2), Central Pacific Hurricane Center (CPHC), and Joint Typhoon Warning Center (JTWC), provide basin-of-record data for all basins, supplemented by the India Meteorological Department (IMD) for the North Indian Ocean, Météo-France La Réunion (RSMC La Réunion) for the South-West Indian Ocean, the Australian Bureau of Meteorology (BoM) for the South Pacific, and the China Meteorological Administration (CMA) and Hong Kong Observatory (HKO) alongside the Japan Meteorological Agency (JMA) for the Western Pacific. Results were independently validated using 1,014 North Atlantic synthetic tropical cyclones from RAFT^53^, which uses ECMWF Reanalysis v5 (ERA5) as the climate background input.

Global land mask and precise coastal distance for land grids were derived from Terra and Aqua combined Moderate Resolution Imaging Spectroradiometer (MODIS) Land Cover Type Yearly L3 (MCD12C1)^70^. Topographic elevation data came from the Global 30 Arc-Second Elevation (GTOPO30)^71^ dataset. Annual ENSO phase classification was based on the Oceanic Niño Index (ONI) obtained from NOAA’s Climate Prediction Center^72^.

Global tropical cyclone landfall precipitation was analyzed using Multi-Source Weighted-Ensemble Precipitation (MSWEP)^73,74^ at 3-h, 10 km resolution. MSWEP provides consistent global coverage for 1979–2024 with high temporal and spatial resolution, demonstrating superior performance across both gauge-dense and data-sparse regions, making it well suited for global tropical cyclone analysis spanning diverse geographical contexts. Climate environmental factors were extracted from ERA5 reanalysis data^75,76^.

### Landfall tropical cyclone selection and decay timescale

We analyzed 885 global landfalling tropical cyclones from 1979 to 2024. Selection criteria included: (a) landfall duration ≥ 24 h; (b) valid intensity data for 24 h post-landfall; (c) clear decay signal without prolonged stagnation; and (d) landfall intensity above tropical depression threshold. The final dataset comprised storms from six basins: Eastern Pacific (EP), North Atlantic (NA), North Indian Ocean (NI), South Indian Ocean (SI), South Pacific (SP), and Western Pacific (WP). Because different agencies report maximum sustained wind speeds averaged over different time scales (1-min for JTWC, 2-min for CMA, and 3-min for IMD, with others reporting 10-min winds), all wind speed records were converted to 10-min mean wind speeds using standard conversion factors^48,77^.

For each tropical cyclone, we fitted the Kaplan–DeMaria exponential decay model to maximum sustained wind speed (V) data from landfall to 24 h (*t* = 0, 6, 12, 18, and 24 h):

1 $${V}_{\left(t\right)}={V}_{(0)}\times {e}^{(\frac{-t}{\tau })}$$

where t is the time after landfall, and τ is the decay timescale, the time for intensity to reach 1/*e* of landfall intensity^9,50,51^. This exponential decay approach has been validated, achieving high performance (adjusted *R*² ≥ 0.9) for most tropical cyclone events^9^. Moreover, the direct decay rate (wind speed loss within 24 h of landfall) was used to independently validate the trends of post-landfall decay rate.

We divided the analysis into early (1979–2001) and late (2002–2024) periods of equal length to facilitate period comparison while capturing the recent acceleration of climate change. To identify the intensity threshold separating opposing decay trends, we employed a sliding window analysis across landfall intensities, calculating late–early period differences using a window of 10 m/s width and 0.1 m/s step, and used linear interpolation to identify the transition point where this difference crossed zero, yielding a global threshold of 29 m/s (10-min maximum wind speed) for classifying strong (*n* = 429) versus weak (*n* = 456) tropical cyclones. Basin-level thresholds were determined using the same procedure applied independently to each basin. We conducted sensitivity analyses using different intensity thresholds and alternative temporal divisions to test the robustness of our findings (Supplementary Text 1; Supplementary Fig. 5).

Temporal trends were assessed using OLS linear regression analysis on a seven-year moving average. Trend significance was evaluated through the t-statistic of the slope coefficient and corresponding *p*-values. Confidence intervals were calculated using bootstrap resamples (*n* = 10,000), and period differences were evaluated using two-tailed Welch’s *t*-tests.

### Landfall storm precipitation and energy

We analyzed tropical cyclone precipitation and energy distribution across coastal-to-inland distances using the MODIS global land cover dataset at 10-km resolution, calculating distance-to-coast for each grid cell. This framework enabled systematic analysis of tropical cyclone impacts by inland penetration distance.

For each tropical cyclone (*n* = 885), we extracted cumulative precipitation within 500km of the storm track, a standard radius capturing the full precipitation footprint^78^ for 24 h post-landfall, consistent with the period for calculating decay timescale. Storms were categorized into four groups based on intensity (strong and weak) and period (early and late). For each category, mean precipitation at each grid cell was computed, excluding cells with <0.1 mm precipitation. Precipitation was then averaged within coastal-distance bins at 50-km intervals. Period differences within each distance bin were assessed using Welch’s *t*-tests.

The power dissipation ndex (PDI)^1^ was calculated (Eq. 2):

2 $${{{\rm{PDI}}}}=\int {V}^{3}{dt}$$

Values are assigned to grid cells within 150 km of storm tracks, the typical radius of destructive winds^79^, using inverse distance weighting at 3-h intervals through 72 h post-landfall. Mean PDI values were also computed for each 50-km coastal distance bin.

### Environmental factors

We analyzed the relationships between tropical cyclone post-landfall decay rates and 10 environmental factors, selected based on established physical mechanisms governing tropical cyclone intensity maintenance. Factors were categorized into three groups: (i) tropical cyclone characteristics (storm’s physical state or surface parameters), including landfall intensity, translation speed, OAR, and topographic height; (ii) thermodynamic features (moisture and temperature conditions), including SST anomaly, specific humidity anomalies at 850 hPa, and soil moisture anomaly; and (iii) dynamic features (atmospheric stability and vorticity), including 850 hPa vorticity, 500 hPa vertical wind speed, and VWS (Supplementary Table 2).

Translation speed was calculated from the 24-h displacement distance post-landfall. OAR represents the proportion of ocean grid points within 150 km of storm centers based on MODIS land cover data, averaged at 6, 12, and 18 h post-landfall^17^ to characterize surface exposure during the decay window. Topographic height was calculated by averaging terrain height within a 150- km radius of tropical cyclone centers at *t* = 0, 6, 12, and 18 h post-landfall.

VWS was defined as the vector magnitude difference between 200 hPa and 850 hPa winds (Eq. 3):

3 $${{{\rm{VWS}}}}=\sqrt{{({U}_{200}-{U}_{850})}^{2}+{({V}_{200}-{V}_{850})}^{2}}$$

where U and V represent the zonal and meridional wind components, respectively. Soil moisture data from the top two layers (0–28 cm) were used to match the daily time scale of tropical cyclone landfall. Dynamic and thermodynamic factors are extracted from ERA5 reanalysis. Considering that temperature and humidity are largely determined by latitude and longitude, thermodynamic factors represent anomalies relative to climatological means over 1979–2024.

### Correlation and trend analysis

To investigate the sensitivity of decay timescale to each environmental factor, we computed Pearson correlation coefficients at two scales. Event-scale correlations were calculated between individual storm decay timescales and the corresponding environmental variable values (*n* = 429 and 456 for strong and weak tropical cyclones, respectively), capturing storm-to-storm variability. Year-scale correlations were calculated between annual mean decay timescales and annual mean environmental variables, capturing long-term co-variation; because both the decay timescale and several environmental variables exhibit substantial trends over the study period, year-scale correlations may reflect shared temporal evolution in addition to direct physical relationships. Statistical significance was assessed using two-tailed *t*-tests.

Long-term trends in decay timescale and individual environmental variables were estimated using OLS linear regression, with confidence intervals derived from bootstrap resamples (*n* = 10,000). Given the limited event counts and data quality uncertainties during the early satellite era, and the absence of strong or weak tropical cyclones in some years during 1979–1983, year-scale correlation and long-term trend analyses were conducted over 1984–2024.

Spatial annual trends in key ERA5 environmental variables (soil moisture, VWS, SST, and 850 hPa specific humidity) over recent decades with most rapid changes (1999–2024) were computed globally at each grid point using OLS regression applied to tropical cyclone-season means (May–October for the Northern Hemisphere; October–March for the Southern Hemisphere), with grid-point significance assessed by two-tailed *t*-test.

### Two-step trend decomposition of decay timescale

To quantify the fractional contribution of key environmental factors to the observed long-term trends in decay timescale, we employed a two-step trend-decomposition approach, following the logic of process- and trend-decomposition analyses^80,81^. In the first step, OLS regression was performed on standardized event-scale data to estimate the sensitivity coefficient (*β*) of decay timescale to each predictor, using all storm events with complete predictor records. To guard against multicollinearity, predictor sets were restricted to four physically motivated variables per intensity category (soil moisture, translation speed, VWS, and 850 hPa vorticity for strong tropical cyclones; SST, OAR, 850 hPa specific humidity, and landfall intensity for weak storms), with multicollinearity assessed via variance inflation factors (VIF < 2 for all retained predictors; Supplementary Fig. 15a, d).

In the second step, the long-term OLS trend (△X) of each predictor’s annual time series was computed. The fractional contribution of each factor to the observed decay timescale trend ($\triangle {\tau }_{{obs}}$) was then estimated (Eq. 4):

4 $${\mbox{Contribution }}(\%)=\frac{{\beta}_{\mbox{unstandardized}} \times \Delta X}{\Delta{\tau}_{\mbox{obs}}} \times 100 $$

where ${\beta }_{{{{\rm{unstandardized}}}}}$ was obtained by multiplying the standardized regression coefficient by the ratio of the standard deviation of decay timescale to that of the corresponding predictor. Robustness was assessed using Ridge regression as a regularized alternative to OLS, with the regularization parameter selected by leave-one-out cross-validation. Statistical significance of each contribution was determined by bootstrap resampling (*n* = 10,000) of the event-scale data. Additional diagnostics included partial correlation controlling for all other retained predictors and leave-one-variable-out sensitivity tests that quantified the change in total explained trend when each physical predictor was excluded (Supplementary Fig. 15). This decomposition should be interpreted as a statistical environmental contribution analysis rather than formal causal attribution, the unexplained residual may reflect variables not included, nonlinear processes, and large-scale circulation variability not captured by the selected local environmental predictors.

### Idealized CM1 simulations

To further examine the intensity-dependent sensitivity, we conducted idealized simulations of tropical cyclone landfall using Cloud Model 1 (CM1, version 18.3)^63–65^, following the experimental design^9^ (Supplementary Table 3). The environmental sounding is based on measurements over the North Atlantic during the hurricane seasons of 1995–2002^82^.

To isolate the sensitivity of post-landfall decay to VWS, tropical cyclones were first spun up over the ocean under a quiescent environment (SST = 301 K, VWS = 0) until reaching target landfall intensities of *V*_0_ = 35, 45, 55, and 65 m/s, with restart files saved at 30-min intervals. Simulations were then restarted from the appropriate restart file under four VWS environments (0, 5, 10, and 15 m/s). VWS was imposed by prescribing a wind profile in the environmental sounding that increases with height from zeronear the surface to yield the target 200–850 hPa wind speed difference, while maintaining zero surface winds to minimize storm translation. Landfall was simulated by setting the enthalpy exchange coefficient (*C*_e_) to zero, while retaining surface momentum drag^9,43,44,52^. The moving domain option was applied for experiments with environmental VWS > 0.

To isolate the sensitivity of post-landfall decay to SST, a parallel set of spin-up simulations was conducted under four SST conditions (300, 301, 302, and 303 K). For each SST, the environmental sounding was modified by (1) uniformly shifting the temperature profile at all altitudes, and (2) adjusting the specific humidity profile to preserve the relative humidity profile^9,83^. All other settings were held constant. Restart files were extracted at target landfall intensities of *V*_0_ = 30, 40, 50, and 60 m/s for each SST condition, and landfall was simulated under the corresponding SST and humidity environment using the same settings as the VWS experiments.

### Model output diagnostics

To characterize the tropical cyclone structural differences across landfall intensities, we computed azimuthally averaged 10-m wind speed profiles and vertical profiles of potential temperature anomaly at the time of landfall (*t* = 0 h) under the VWS = 0, SST = 301 K control condition.

The potential temperature anomaly (*θ'*) was defined as the difference between the area-averaged potential temperature within 50 km of the tropical cyclone center and the azimuthal mean between 150 and 250 km radius environmental reference at each vertical level^84,85^, providing a measure of warm-core intensity and depth. A stronger and vertically coherent warm core indicates a more organized, energetically intense vortex structure^84,86^.

To quantify VWS-induced structural disruption, we diagnosed vortex tilt as the horizontal displacement between the surface pressure-minimum center and the pressure-minimum center at each vertical level. Vortex tilt reflects the degree of vertical misalignment imposed by environmental shear; larger tilt indicates greater disruption of the coherent vortex column^20,87^. To assess the thermodynamic consequences of VWS, we computed the difference in $\theta {\prime}$ profiles, a reduction in upper-level $\theta {\prime}$ indicates a weakening of the warm-core structure, related to shear-induced ventilation and inner-core asymmetry^88^.

To quantify the intensity-dependent energetic response to SST warming, we defined an energy surplus ratio (Eq. 5):

5 $$S(t)=\frac{{H}_{sst303}\left(t\right)-{H}_{sst300}\left(t\right)}{{E}_{{\mbox{fric}},sst300,{\mbox{cum}}}\left(t\right)}$$

where ${H}_{{SST}}(t)$ is the column-integrated moist static energy averaged within 150 km of the tropical cyclone center (Eq. 6):

6 $$H\left(t\right)=\int \rho h\,{dz}$$

7 $$h={c}_{{{{\rm{p}}}}}T+{gz}+{L}_{{{{\rm{v}}}}}q$$

h is the moist static energy (J kg⁻¹, Eq. 7), where ${c}_{p}$ is the specific heat of dry air at constant pressure, T is temperature, g is gravitational acceleration, z is height, ${L}_{{{{\rm{v}}}}}$ is the latent heat of vaporization, and q is water vapor mixing ratio; ρ is air density; and the overbar denotes the horizontal area average within 150 km. The resulting H has units of J m⁻^2^.

The cumulative surface frictional energy dissipation is (Eq. 8):

8 $${E}_{{fric},sst300,{cum}}\left(t\right)={\int }_{\!\!\!\!\!0}^{t}{\rho }_{s}{C}_{d}{\left|{V}_{10}\right|}^{3}\,dt$$

where ${\rho }_{s}$ is near-surface air density, ${C}_{d}$ is the drag coefficient, and $\left|{V}_{10}\right|$ is the 10-m wind speed, also area-averaged within 150 km of tropical cyclone center (J m^−2^). S(t) is dimensionless and quantifies how effectively SST-warming-induced additional column energy offsets the storm’s frictional energy consumption after landfall. We note that ${E}_{{fric},sst300,{cum}}$ accounts only for surface momentum dissipation; total atmospheric energy dissipation is much larger, so S(t) underestimated the proportion of total friction energy dissipation.

## Supplementary information

Supplementary Information

Transparent Peer Review file

## Source data

Source Data

## Data Availability

International Best Track Archive for Climate Stewardship (IBTrACS) V4.0.1 dataset is available from NOAA National Centers for Environmental Information (https://www.ncei.noaa.gov/products/international-best-track-archive). North Atlantic synthetic tropical cyclone track, intensity, and rainfall dataset from RAFT can be found in Zenodo (https://zenodo.org/records/10392725). The European Centre for Medium-Range Weather Forecasts Reanalysis V5 (ERA5) data on pressure levels is available from ECMWF (https://cds.climate.copernicus.eu/datasets/reanalysis-era5-pressure-levels-monthly-means?tab=download). The ERA5 data on single levels is available from ECMWF (https://cds.climate.copernicus.eu/datasets/reanalysis-era5-single-levels-monthly-means?tab=download). The Terra and Aqua combined Moderate Resolution Imaging Spectroradiometer (MODIS) Land Cover Type Yearly L3 (MCD12C1) is available from NASA Earth Data (https://ladsweb.modaps.eosdis.nasa.gov/missions-and-measurements/products/MCD12C1#data-availability). The Global 30 Arc-Second Elevation (GTOPO30) is available from USGS (https://www.usgs.gov/centers/eros/science/usgs-eros-archive-digital-elevation-global-30-arc-second-elevation-gtopo30). The Multi-Source Weighted-Ensemble Precipitation (MSWEP) data are available from GloH2O upon registration (https://www.gloh2o.org/mswep/). The Oceanic Niño Index data is obtained from NOAA’s Climate Prediction Center (https://www.cpc.ncep.noaa.gov/products/analysis_monitoring/ensostuff/ONI_v5.php). The processed tropical cyclone decay timescale, precipitation, environmental sensitivity and model simulation datasets generated in this study are deposited at Zenodo (https://doi.org/10.5281/zenodo.21863583). Source data are provided with this paper.

## Code availability

All analysis code used to produce the results and figures in this study is publicly available at Zenodo (https://doi.org/10.5281/zenodo.21863583).
