# Supplementary material for: Divergent trends in post-landfall decay of strong and weak tropical cyclones
Source: Nat Commun. 2026 Sep 24;17:11099. doi: 10.1038/s41467-026-77958-3 (PMC13696725; doi:10.1038/s41467-026-77958-3)
Supplement: Supplementary file 1 [file 41467_2026_77958_MOESM1_ESM.pdf]

# Supplementary Information for

## Divergent trends in post-landfall decay of strong and weak tropical cyclones

[1]Chenxi Hu, [2,3]Xinxin Sui, [1,4]Dev Niyogi, [5,6]Clint N. Dawson, and [*1]Zong-Liang Yang

[1]Department of Earth and Planetary Sciences, Jackson School of Geosciences, The University of Texas at Austin, Austin, TX, USA

[2]National Science Foundation, National Center for Atmospheric Research, Boulder, CO, USA

[3]Civil and Environmental Engineering, Colorado School of Mines, Golden, CO, USA

[4]Department of Civil, Architectural, and Environmental Engineering, The University of Texas at Austin, Austin, TX, USA

[5]Department of Aerospace Engineering and Engineering Mechanics, Cockrell School of Engineering, The University of Texas at Austin, Austin, TX, USA

[6]Oden Institute for Computational Engineering and Sciences, The University of Texas at Austin, Austin, USA

[*]Corresponding Author: Zong-Liang Yang

Email: liang@jsg.utexas.edu

**This Supplementary Information includes:**

Supplementary Text 1

Supplementary Figure 1 to 15

Supplementary Table 1 to 3

Supplementary References

## Supplementary Text

### 1. Robustness analysis: alternative intensity thresholds and temporal splits

To verify the robustness of our findings beyond intensity threshold and period division selection (Supplementary Fig. 5), analysis was extended to compared results using alternative intensity threshold (28 m/s and 30 m/s) and temporal divisions (1979–2000 vs 2001–2024 and 1979–2002 vs 2003–2024). Results yielded consistent intensity-dependent trends with different intensity threshold: weak tropical cyclones showed significant increasing decay timescales (Threshold = 28 m/s: 0.2 h/yr, 95% confidence interval (CI): 0.13–0.27, $P < 0.01$; Threshold = 28 m/s: 0.23 h/yr, 95% CI: 0.16–0.29, $P < 0.01$), while strong tropical cyclones demonstrated decreasing trends (Threshold = 28 m/s: –0.17 hours/yr, 95% CI: –0.22 to –0.11, $P < 0.01$; Threshold = 30 m/s: –0.20 hours/yr, 95% CI: –0.27 to –0.12, $P < 0.01$; Supplementary Fig. 5a, c). Comparing early (1979–2001) and late (2002–2024) periods confirmed the intensity-dependent bifurcation pattern under different intensity threshold, with weak cyclones exhibiting significant longer mean decay timescales by about 14.9%/14.1% (Threshold = 28 m/s: 36.1 versus 41.5 hours, 95% CI: 33.4–38.9 versus 38.6–44.5; Threshold = 30 m/s: 35.4 versus 40.4 hours, 95% CI: 32.9–37.1 versus 37.7–43.1) and strong cyclones showing faster decay by about 15.2%/20.2% (Threshold = 28 m/s: 33.6 versus 27.3 hours, 95% CI: 29.6–35.6 versus 25.9–29.3; Threshold = 28 m/s: 32.7 versus 26.1 hours, 95% CI: 29.3–36.2 versus 24.6–27.6; Supplementary Fig. 5b, d).

Similarly, comparisons using alternative temporal splits yielded results highly consistent with our primary analysis, with weak cyclones exhibiting significant longer mean decay timescales by about 14.6%/14.6% (Division = 2000: 36.0 versus 41.2 hours, 95% CI: 33.2–38.7 versus 38.4–44.1; Division = 2002: 36.1 versus 41.4 hours, 95% CI: 33.6–38.6 versus 38.5–44.3) and strong cyclones showing faster decay by about 17.3%/17.8% (Division = 2000: 31.9 versus 26.4 hours, 95% CI: 28.6–35.2 versus 24.9–27.9; Division = 2002: 32.2 versus 26.4 hours, 95% CI: 29.0–35.2 versus 24.9–27.9; Supplementary Figs. 5e, f). The robustness across multiple intensity threshold and temporal divisions confirms the robustness observed patterns.

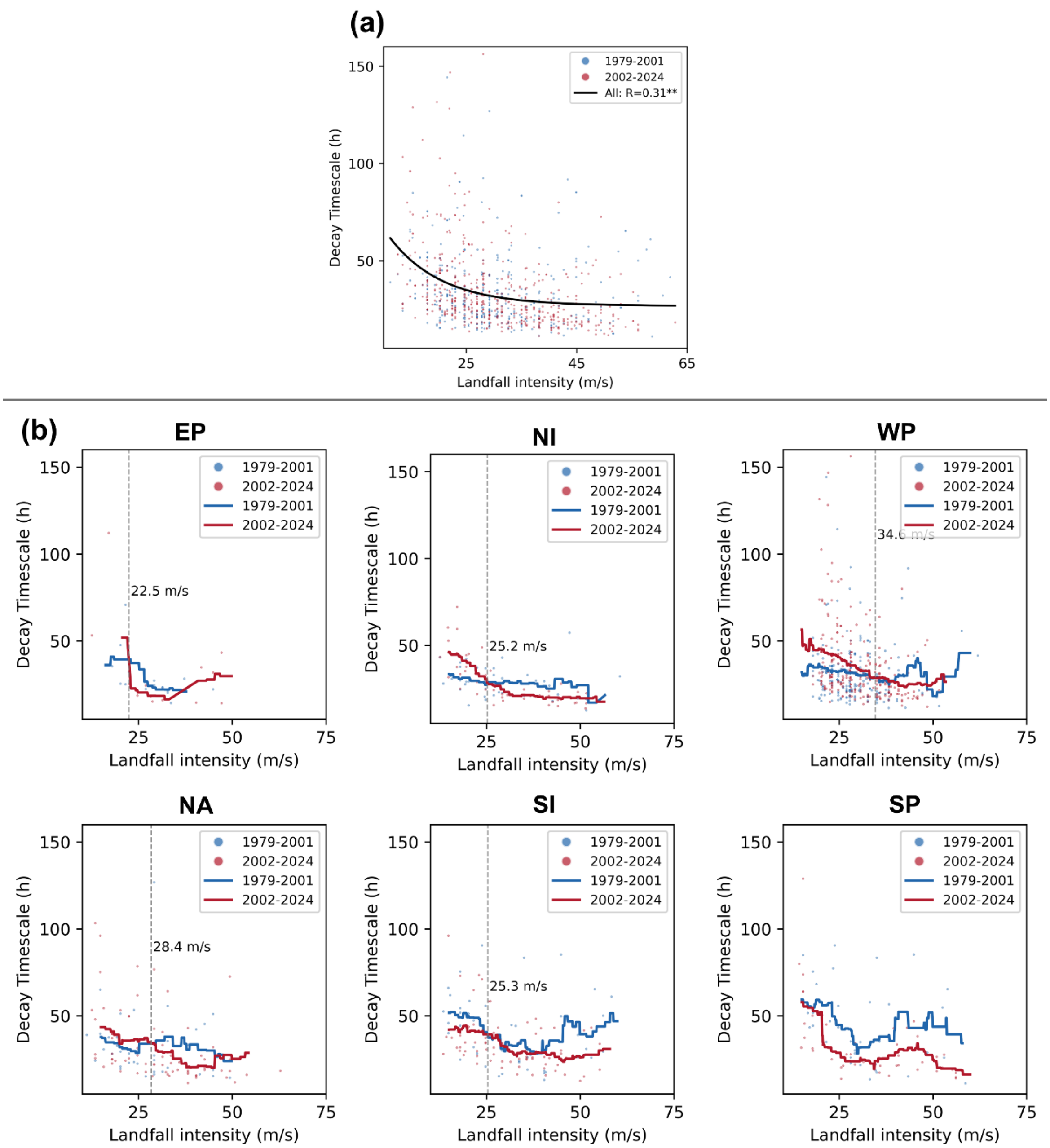


**Supplementary Fig. 1. (a)** Relationship between landfall intensity (m/s) and post-landfall decay timescale (h) for tropical cyclones during early (blue dots) and late (red dots) periods for global events, with fitted exponential curves shown in black curve with correlation coefficient (R) and significant level. Asterisks denote significant relationship (** $P < 0.01$). **(b)** Relationship between landfall intensity and decay timescale for landfall tropical cyclones in specific basin: Eastern Pacific (EP), North Atlantic (NA), North Indian Ocean (NI), South Indian Ocean (SI), Southern Pacific (SP), and Western Pacific (WP); each dot represents an individual landfall event. Blue and red lines show the running mean of decay timescale across landfall intensity bins; dashed vertical line marks the intensity threshold at which the difference in decay timescale between the two periods reverses sign.

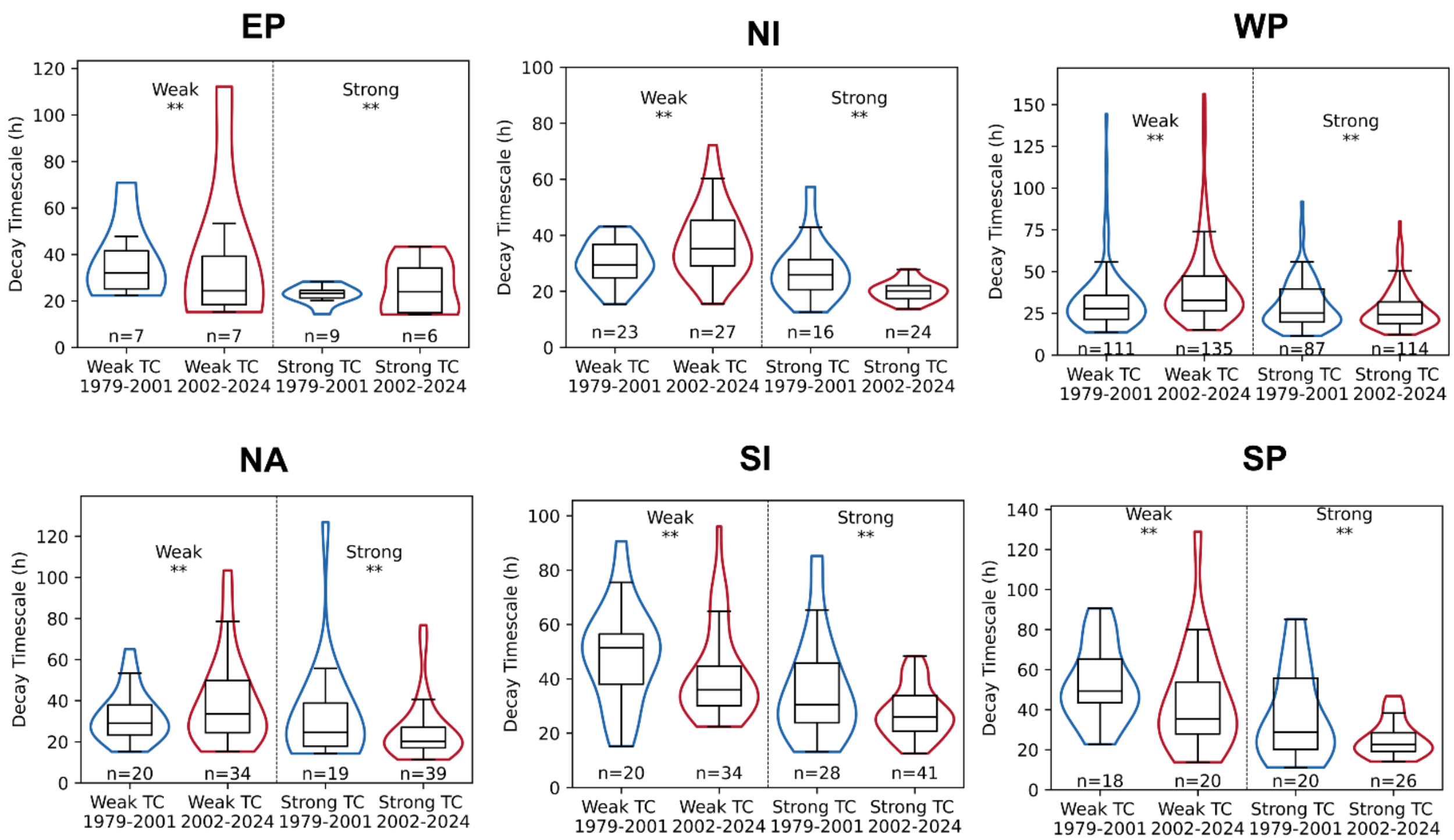


**Supplementary Fig. 2.** Distribution of decay timescales across intensity categories and periods in each basin: Eastern Pacific (EP), North Atlantic (NA), North Indian Ocean (NI), South Indian Ocean (SI), Southern Pacific (SP), and Western Pacific (WP). Violin plots show kernel density estimates of the full distribution; overlaid box plots indicate the median (center line), interquartile range (box limits), and 1.5× interquartile range (whiskers); outliers are not shown. Sample sizes indicated as "*n*". Asterisks denote significant differences between periods (** $P < 0.01$).

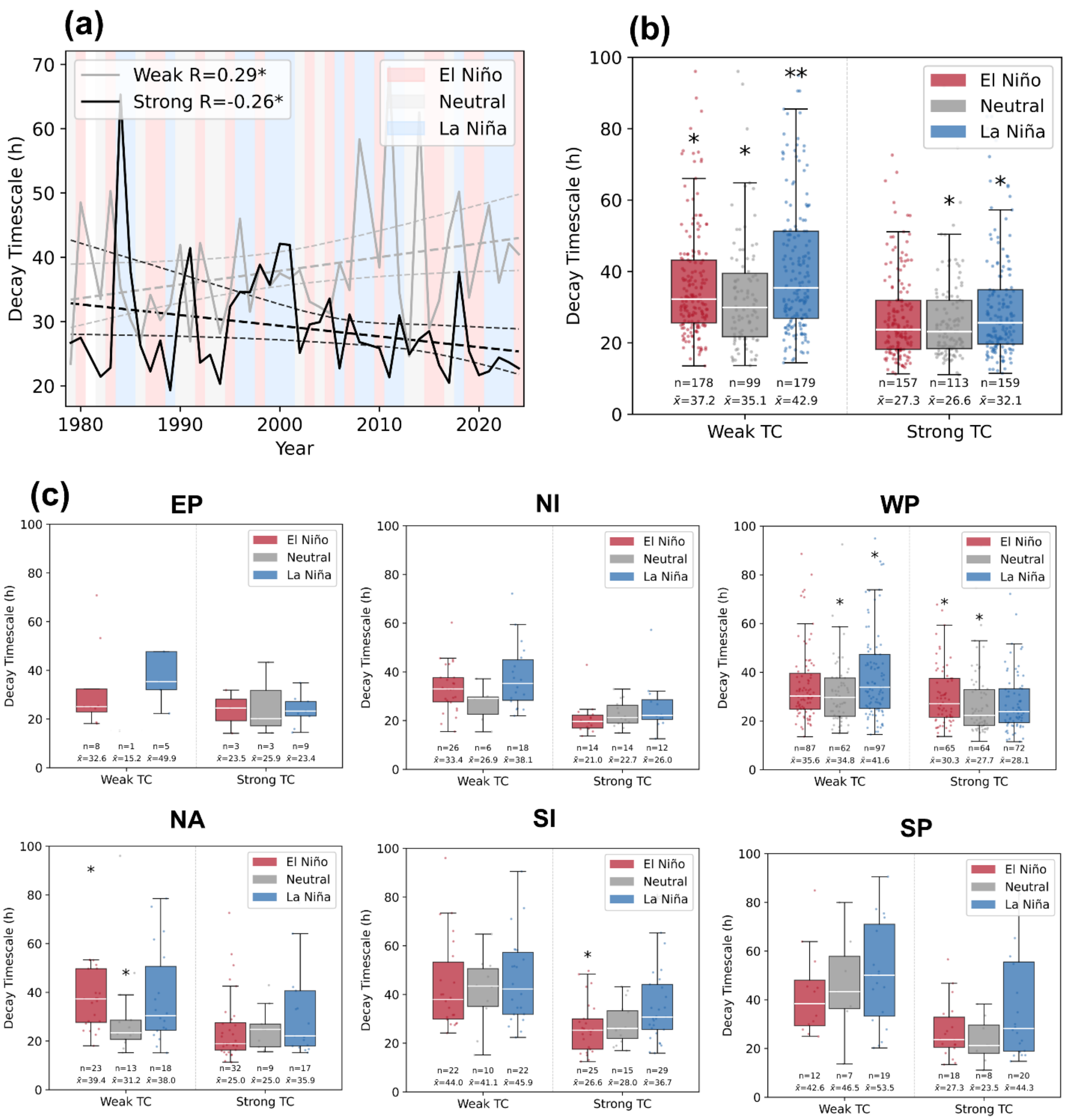


**Supplementary Fig. 3. (a)** Temporal trends in annual mean decay timescale for strong and weak tropical cyclones. Trend coefficients ($R$), 95% bootstrap confidence interval (CI), and significance levels are shown. **(b)** Box plots of global decay timescale distributions for weak and strong tropical cyclones stratified by El Niño-Southern Oscillation (ENSO) phase: El Niño, Neutral, and La Niña, pooled across all six ocean basins. ENSO phase is classified using the December–February Oceanic Niño Index (ONI), with El Niño defined as ONI $\geq +0.5$, La Niña as ONI $\leq -0.5$, and Neutral otherwise. Boxes show the interquartile range, with the white horizontal line indicating the median; whiskers extend to 25% and 75% range. Individual events are overlaid as scatter points. Sample size ($n$) and mean ($\bar{x}$, in hours) are annotated below each box. Asterisks above boxes indicate statistical significance of pairwise Mann–Whitney U tests between ENSO phases within each intensity group (* significant against at least one other phase at $P < 0.05$; ** significant against both other phases at $P < 0.05$). **(c)** same as **(b)** except for specific basin: Eastern Pacific (EP), North Atlantic (NA), North Indian Ocean (NI), South Indian Ocean (SI), Southern Pacific (SP), and Western Pacific (WP).

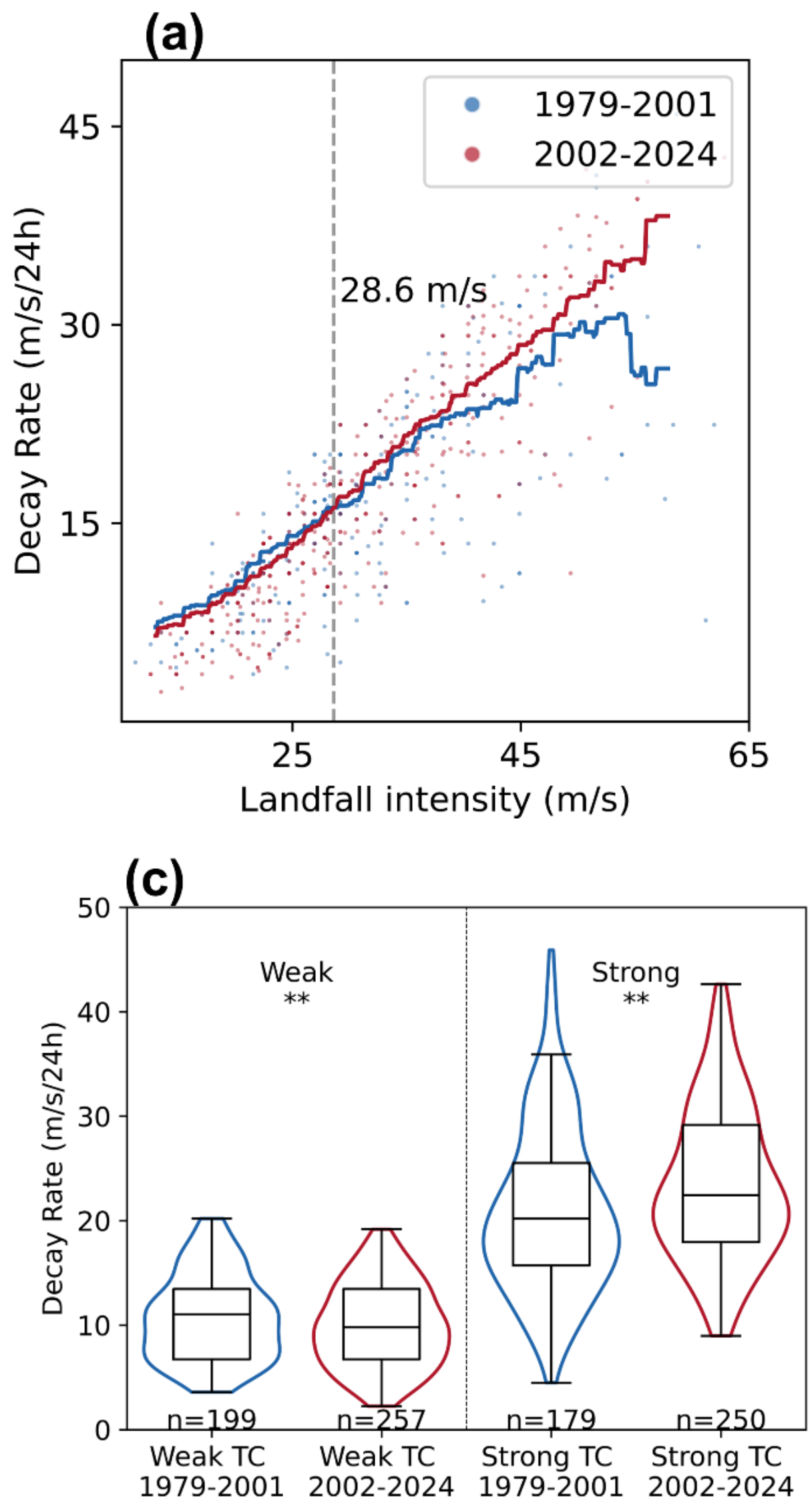


**Supplementary Fig. 4. (a)** Relationship between landfall intensity and direct decay rate (wind speed loss within 24 hours of landfall) for global landfall tropical cyclone. Blue and red lines show the running mean of decay timescale across landfall intensity bins during early and late period; dashed vertical line marks the intensity threshold (28.6 m/s) at which the difference in decay rate between the two periods reverses sign. **(b)** Temporal trends in decay rate for strong and weak tropical cyclones using seven-year running means. Dashed lines show linear regression fits; outer dashed lines indicate the 95% bootstrap confidence interval (CI). Correlation coefficients (*R*) and significance levels are annotated; vertical dashed line marks the period boundary (2001). **(c)** Distribution of decay rate across intensity categories and periods. Violin plots show kernel density estimates of the full distribution; overlaid box plots indicate the median (center line), interquartile range (box limits), and 1.5× interquartile range (whiskers); outliers are not shown. Sample sizes indicated as “*n*”. Asterisks denote significant differences between periods (** $P < 0.01$). **(d)** Annual trend of decay rate for global tropical cyclones, with red, blue, and light blue lines representing annual maximum, annual mean, and annual minimum. Trend coefficients, 95% bootstrap CI, and significance levels are shown.

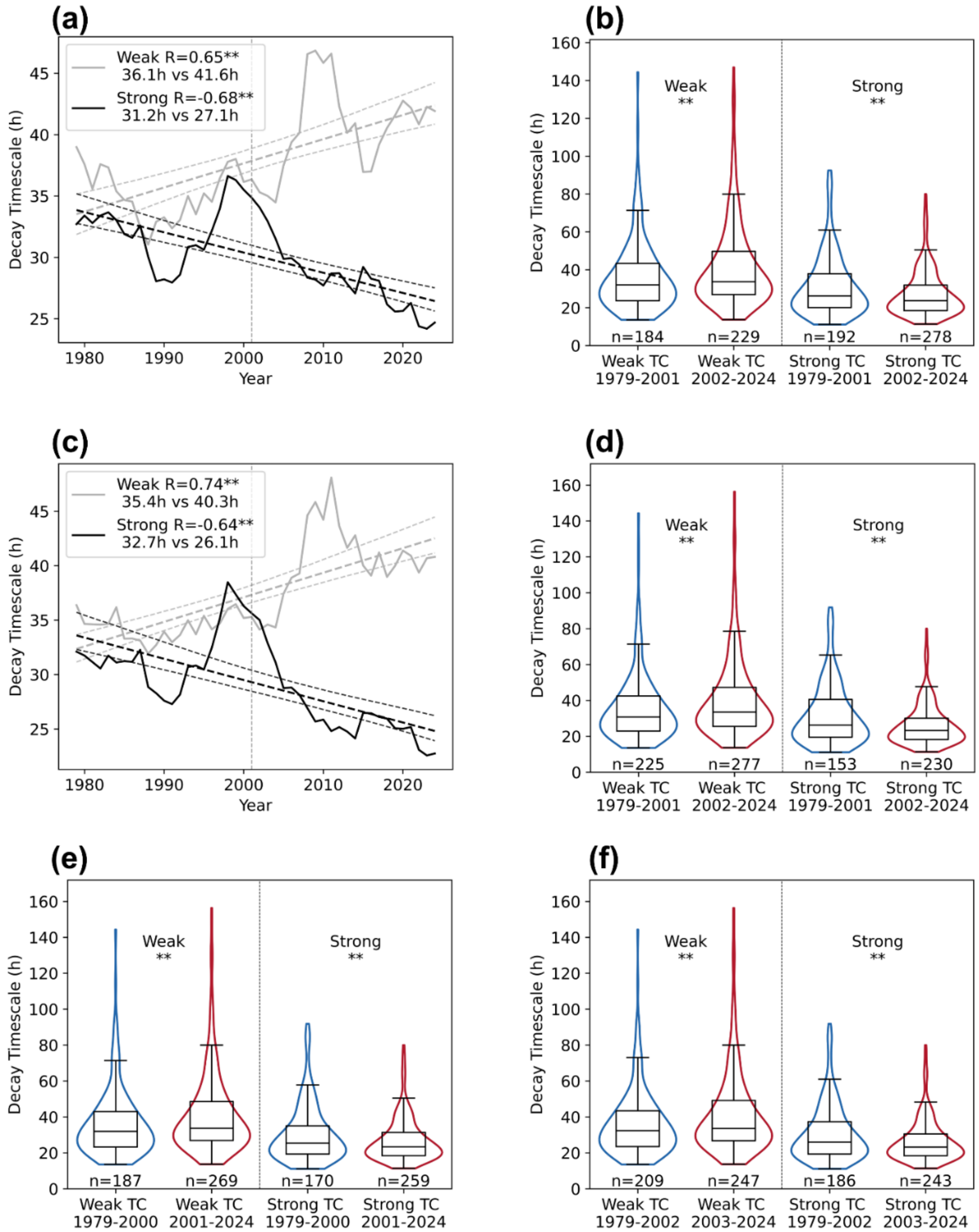


**Supplementary Fig. 5. (a)** Temporal trends in annual mean decay timescale, for strong and weak tropical cyclones (threshold=28 m/s). Correlation coefficients ($R$) and significance levels are annotated; vertical dashed line marks the period boundary (2001). **(b)** Distribution of decay timescales across intensity categories and periods (threshold=28 m/s). Violin plots show kernel density estimates of the full distribution; overlaid box plots indicate the median (center line), interquartile range (box limits), and 1.5× interquartile range (whiskers); outliers are not shown. Sample sizes indicated as "*n*". Asterisks denote significant differences between periods (** $P < 0.01$). **(c), (d)** same as **(a), (b)** except for threshold = 30 m/s. **(e), (f)** same as **Fig. 2b** except for alternative temporal splits: 1979–2000 versus 2001–2024, and 1979–2002 versus 2003–2024.

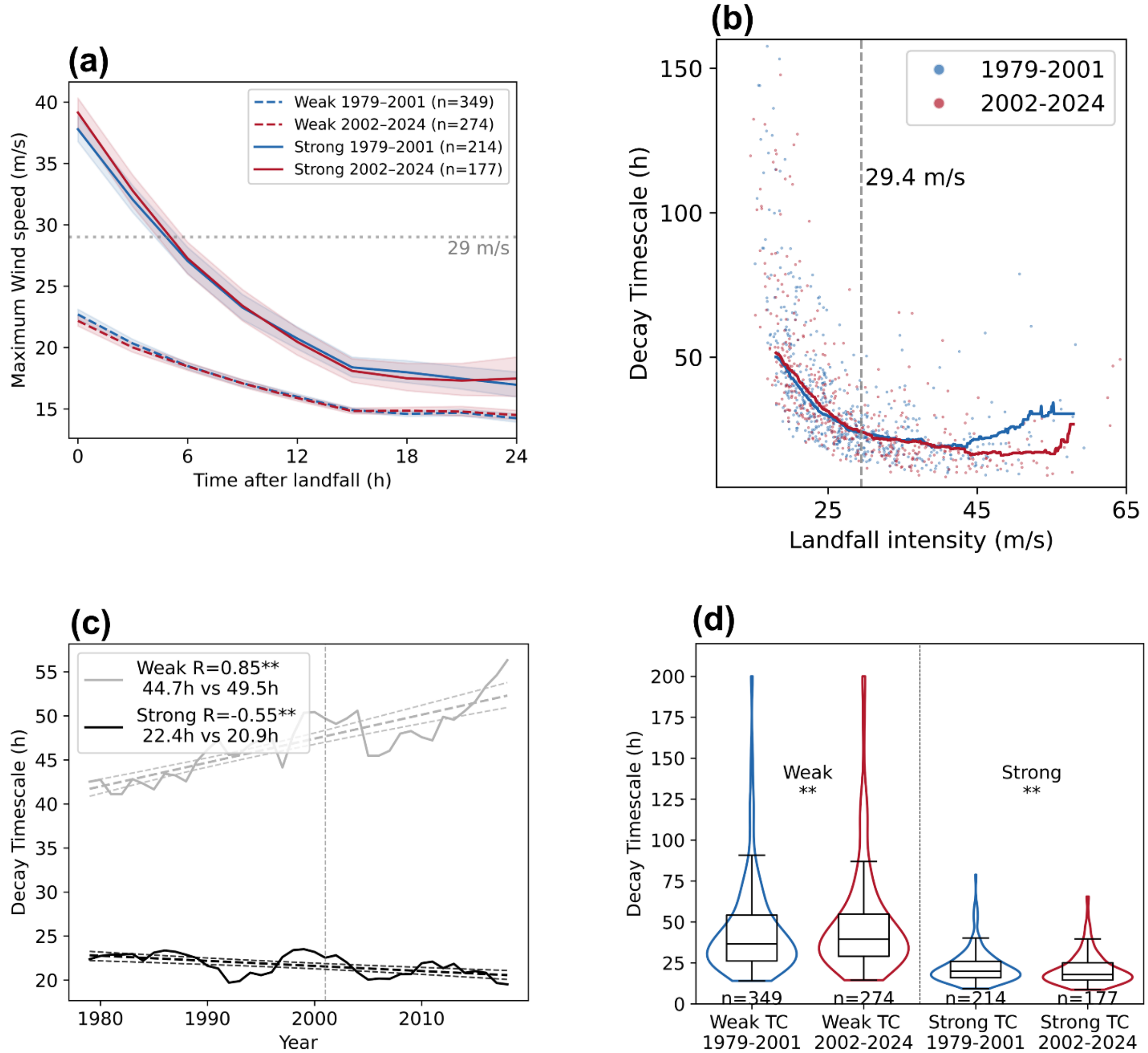


**Supplementary Fig. 6. (a)** Time series of post-landfall wind speed for strong (solid line) and weak (dashed line) tropical cyclones from RAFT dataset, showing early (blue, 1979–2001) versus late (red, 2002–2024) period means; shading denotes the 95% bootstrap CI (*n* = 10,000 resamples). **(b)** Relationship between landfall intensity and decay timescale for 1014 RAFT synthetic North Atlantic tropical cyclones; each dot represents an individual landfall event. Blue and red lines show the running mean of decay timescale across landfall intensity bins during early and late period; dashed vertical line marks the intensity threshold (29.4 m/s) at which the difference in decay timescale between the two periods reverses sign. **(c)** Temporal trends in annual mean decay timescale for RAFT strong and weak tropical cyclones. Dashed lines show linear regression fits; outer dashed lines indicate the 95% bootstrap CI. Correlation coefficients ($R$) and significance levels are annotated; vertical dashed line marks the period boundary (2001). **(d)** Distribution of decay timescales across intensity categories and periods. Violin plots show kernel density estimates of the full distribution; overlaid box plots indicate the median (center line), interquartile range (box limits), and 1.5× interquartile range (whiskers); outliers are not shown. Sample sizes indicated as "*n*". Asterisks denote significant differences between periods (** $P < 0.01$).

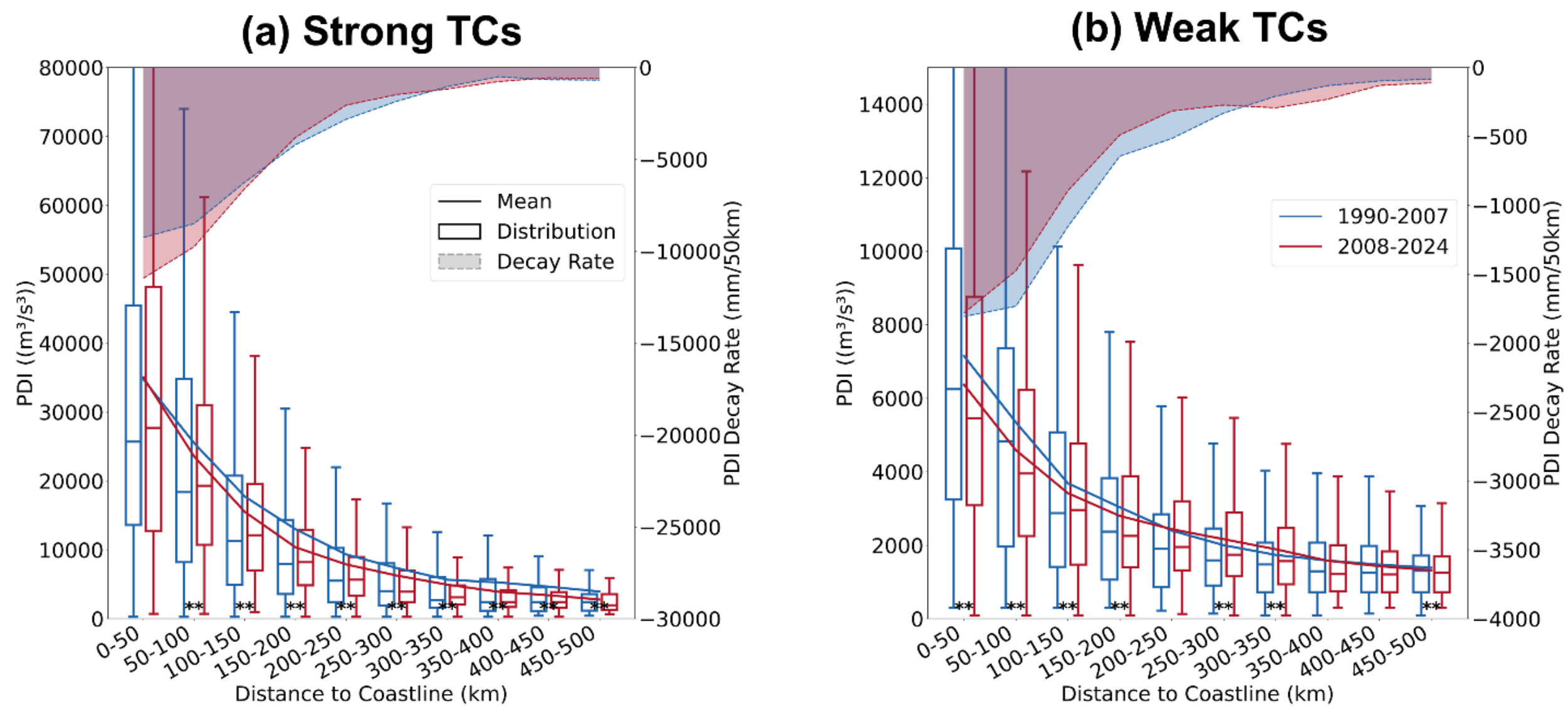


**Supplementary Fig. 7.** Accumulated Power Dissipation Index (PDI) after landfall as a function of distance to coastline for **(a)** strong and **(b)** weak tropical cyclones (TC). Lines show mean PDI for each 50-km distance bin; box plots indicate the median (center line), interquartile range (box limits), and 1.5× interquartile range (whiskers); outliers are not shown. Blue and red colors correspond to the early (1979–2001) and late (2002–2024) periods, respectively. Shaded areas on the secondary axis show the rate of PDI decay with increasing distance from the coastline (mm/50 km). Asterisks denote statistically significant differences in PDI between periods (* $P < 0.05$, ** $P < 0.01$).

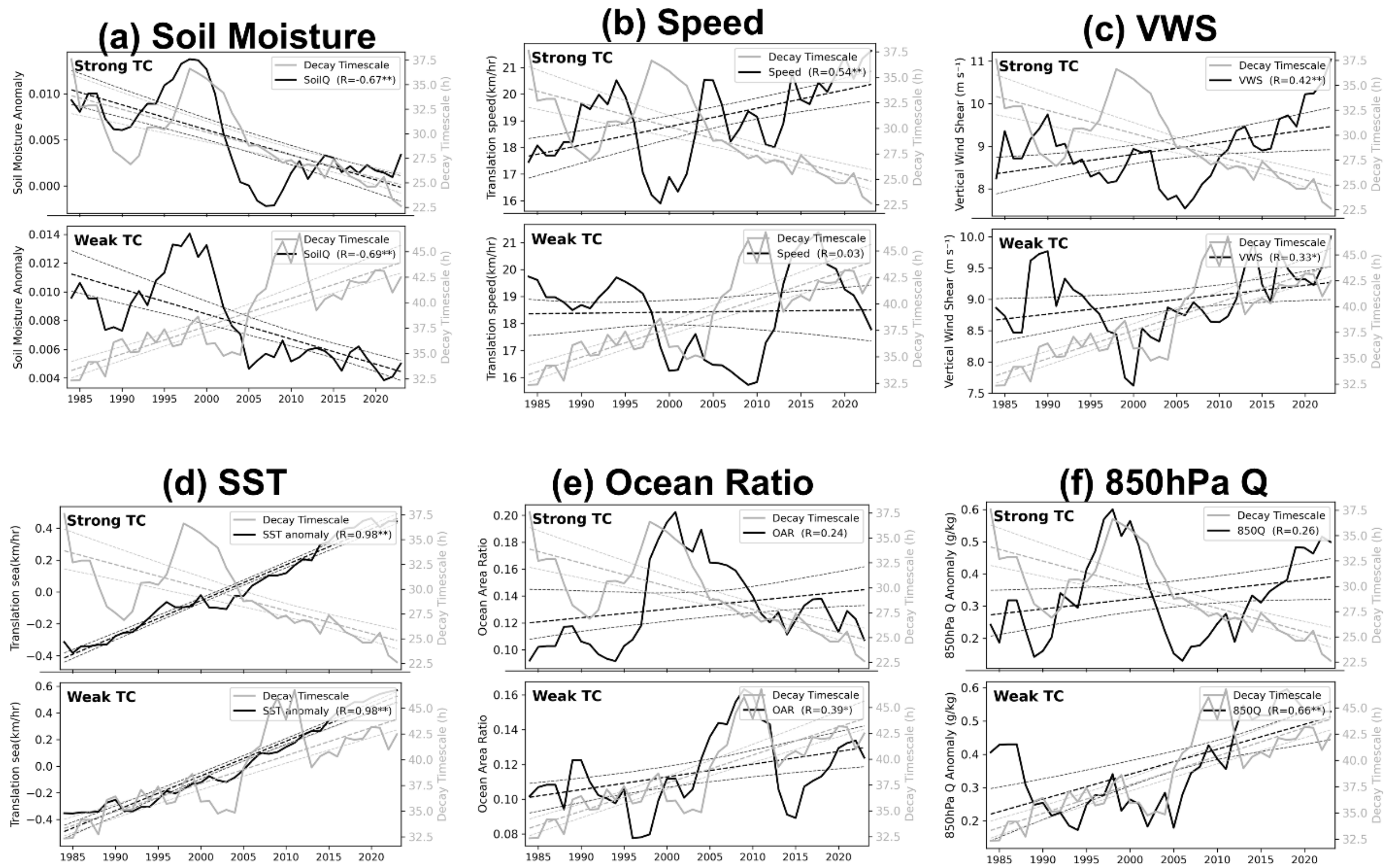


**Supplementary Fig. 8.** Time series in key environmental variables combined with tropical cyclone decay timescale for strong and weak storms. Each column shows the seven-year running mean time series of environmental variable (black line) alongside the decay timescale (gray line) for strong (upper sub-panel) and weak tropical cyclones (lower sub-panel): **(a)** soil moisture anomaly, **(b)** translation speed, **(c)** vertical wind shear (VWS), **(d)** sea surface temperature (SST) anomaly, **(e)** Ocean area ratio (OAR), and **(f)** 850-hPa specific humidity anomaly. Dashed lines show linear regression fits; outer dashed lines indicate the 95% bootstrap confidence interval (CI). Correlation coefficients (*R*) and significance levels are annotated (* $P < 0.05$, ** $P < 0.01$).

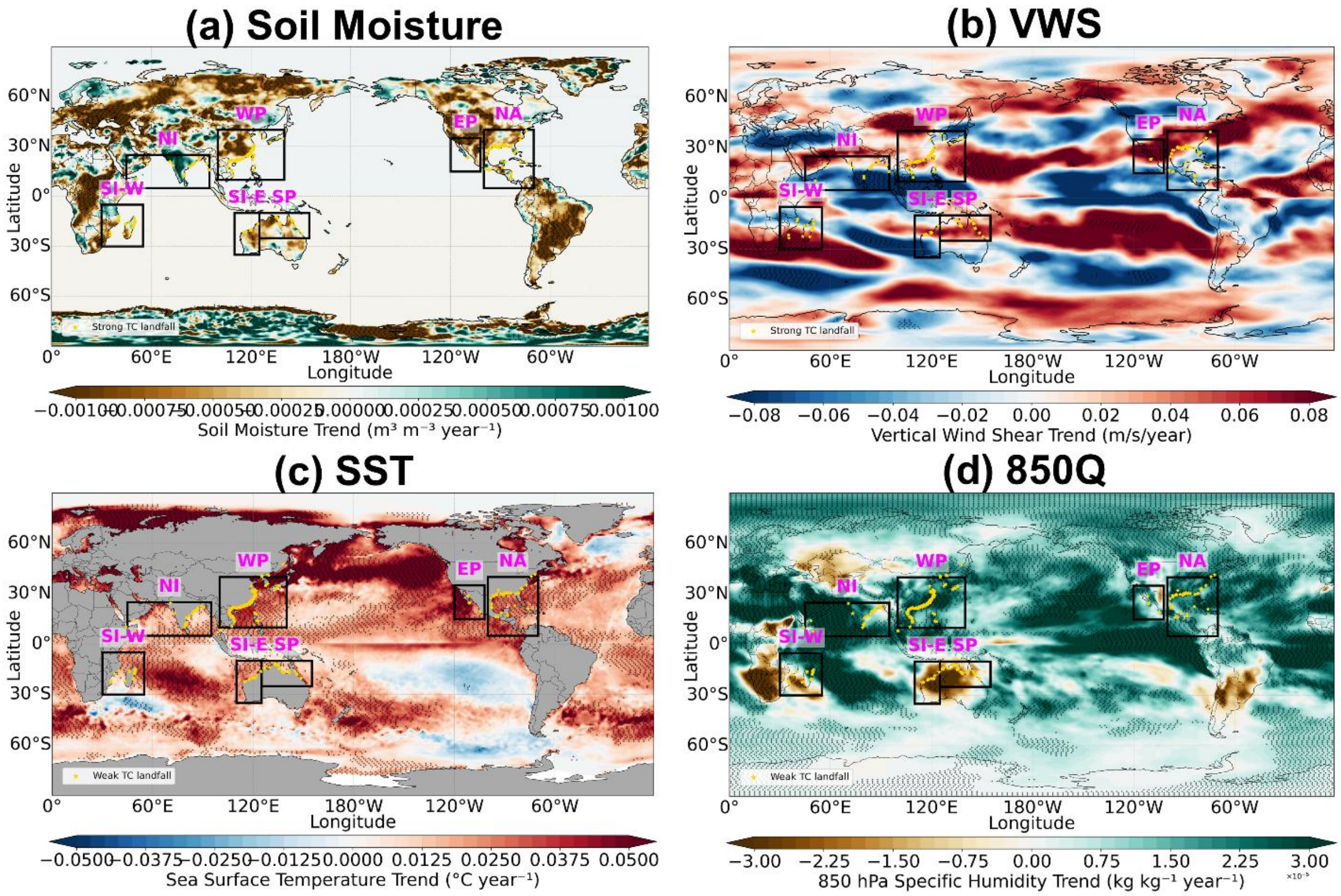


**Supplementary Fig. 9.** Global spatial distribution of annual trends during 1999 to 2024 in key environmental variables **(a)** soil moisture, **(b)** vertical wind shear (VWS), **(c)** sea surface temperature (SST), and **(d)** 850-hPa specific humidity, computed over the Northern Hemisphere tropical cyclone season (May–October) and Southern Hemisphere tropical cyclone season (October–March). Stippling indicates grid points where the trend passed 95% statistically significant level ($P < 0.05$). Black rectangles delineate the six storm basin regions: Eastern Pacific (EP), North Atlantic (NA), North Indian Ocean (NI), South Indian Ocean (SI), Southern Pacific (SP), and Western Pacific (WP). Gold stars denote tropical cyclone landfall locations during 1999–2024 with strong tropical cyclone landfalls shown in **(a)** and **(b)**, and weak landfalls shown in **(c)** and **(d)**.

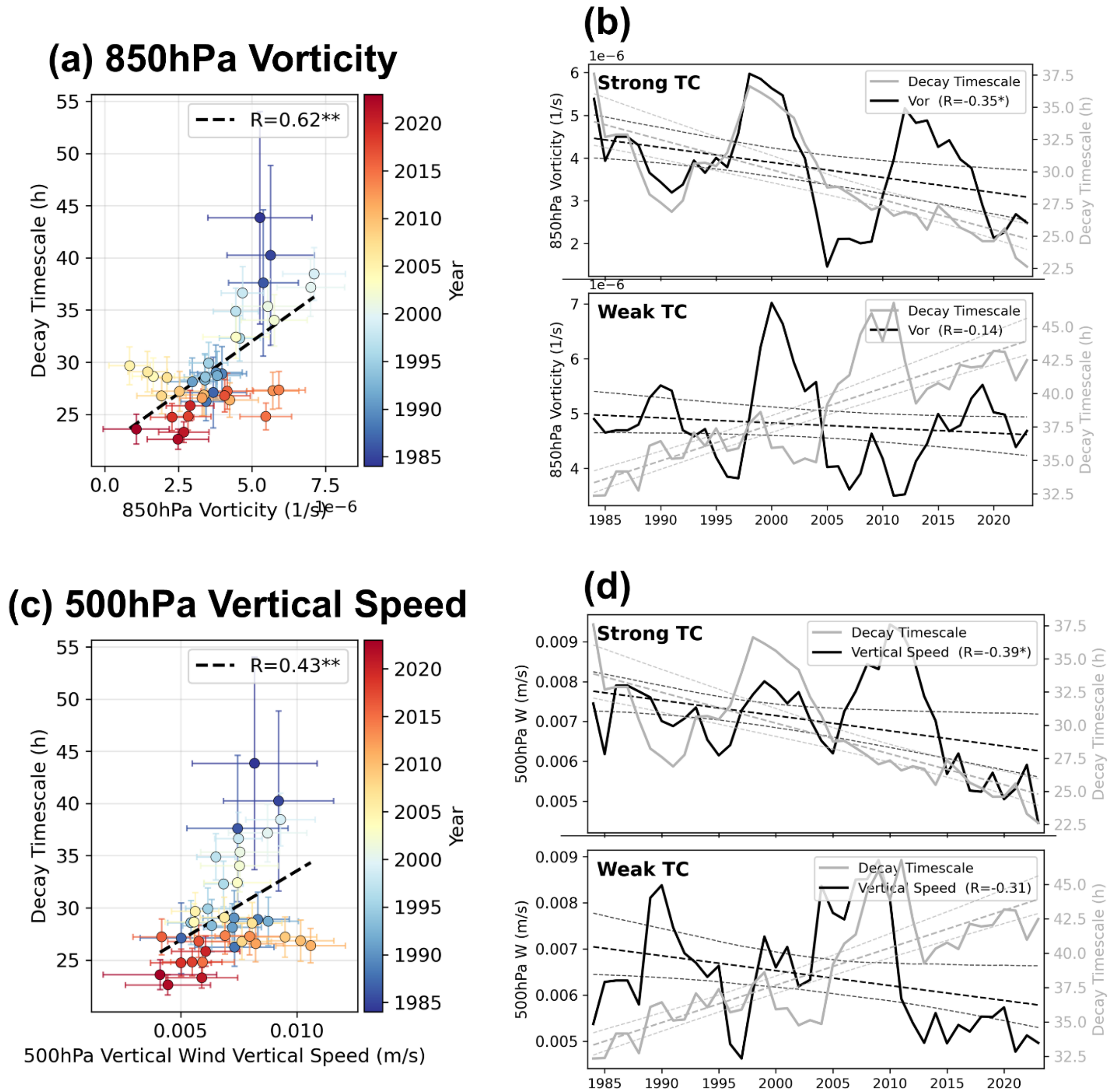


**Supplementary Fig. 10. (a)** Scatter plot between annual mean decay timescale and 850-hPa vorticity for strong tropical cyclones (TC), with dot color indicating year and error bars denoting ±1 standard error of five-year window. The dashed line shows the ordinary least squares (OLS) regression fit with the Pearson correlation coefficient (*R*) and significance level indicated (** $P < 0.01$). **(b)** Time series of 850-hPa vorticity (black line) and decay timescale (gray line) for strong tropical cyclones (upper sub-panel) and weak tropical cyclones (lower sub-panel). **(c, d)** As in **(a)** and **(b)** but for 500-hPa vertical wind speed. Dashed lines show linear regression fits; outer dashed lines indicate the 95% bootstrap confidence interval (CI). Correlation coefficients and significance levels are annotated (* $P < 0.05$, ** $P < 0.01$).

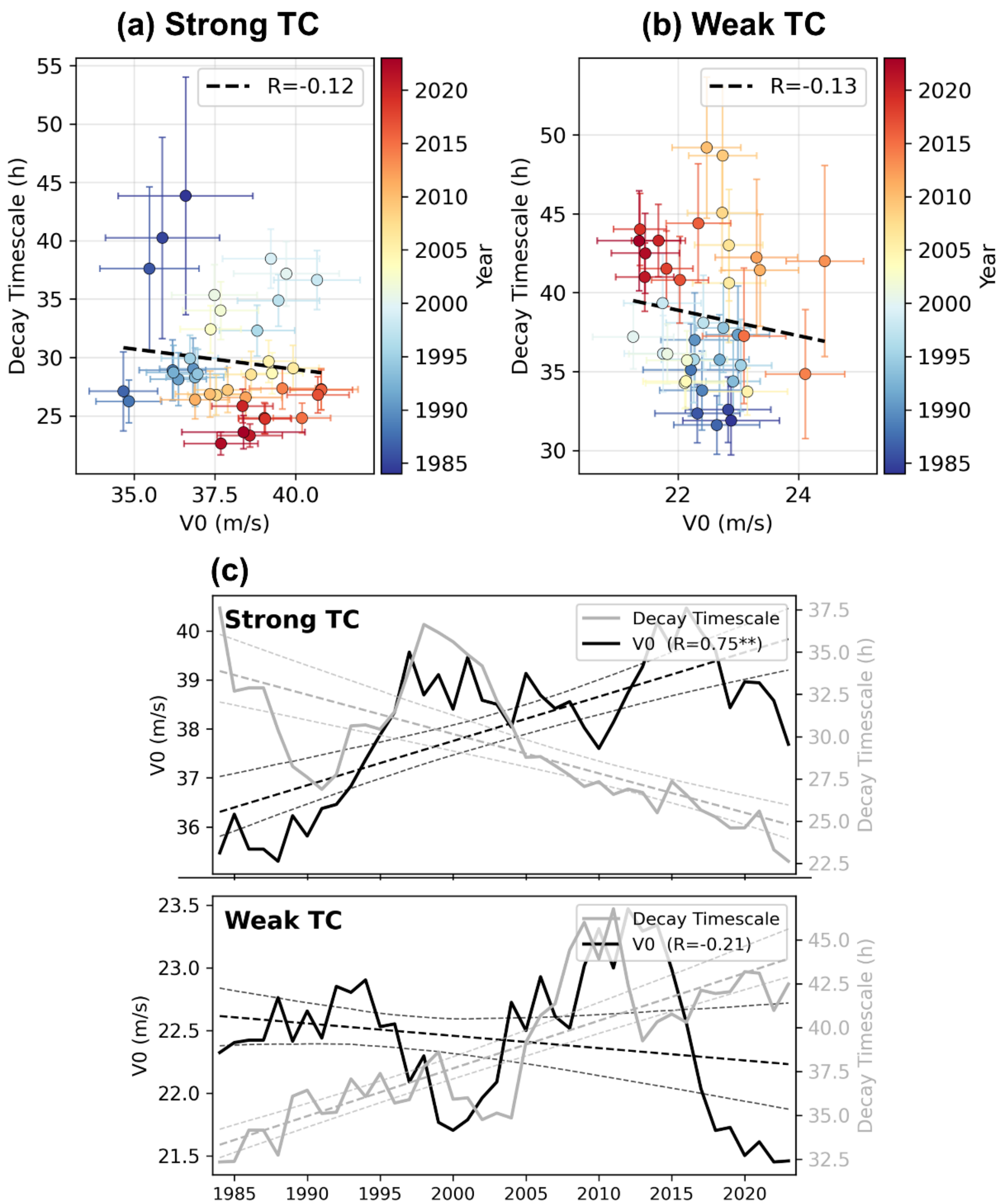


**Supplementary Fig. 11.** Scatter plots between annual mean decay timescale and landfall intensity for **(a)** strong and **(b)** weak tropical cyclones (TC), with dot color indicating year and error bars denoting ±1 standard error of five-year window. The dashed line shows the ordinary least squares (OLS) regression fit with the Pearson correlation coefficient (*R*) and significance level indicated (** $P < 0.01$). **(c)** Time series of landfall intensity (black line) and decay timescale (gray line) for strong (upper sub-panel) and weak tropical cyclones (lower sub-panel). Dashed lines show linear regression fits; outer dashed lines indicate the 95% bootstrap confidence interval (CI). Correlation coefficients and significance levels are annotated (* $P < 0.05$, ** $P < 0.01$).

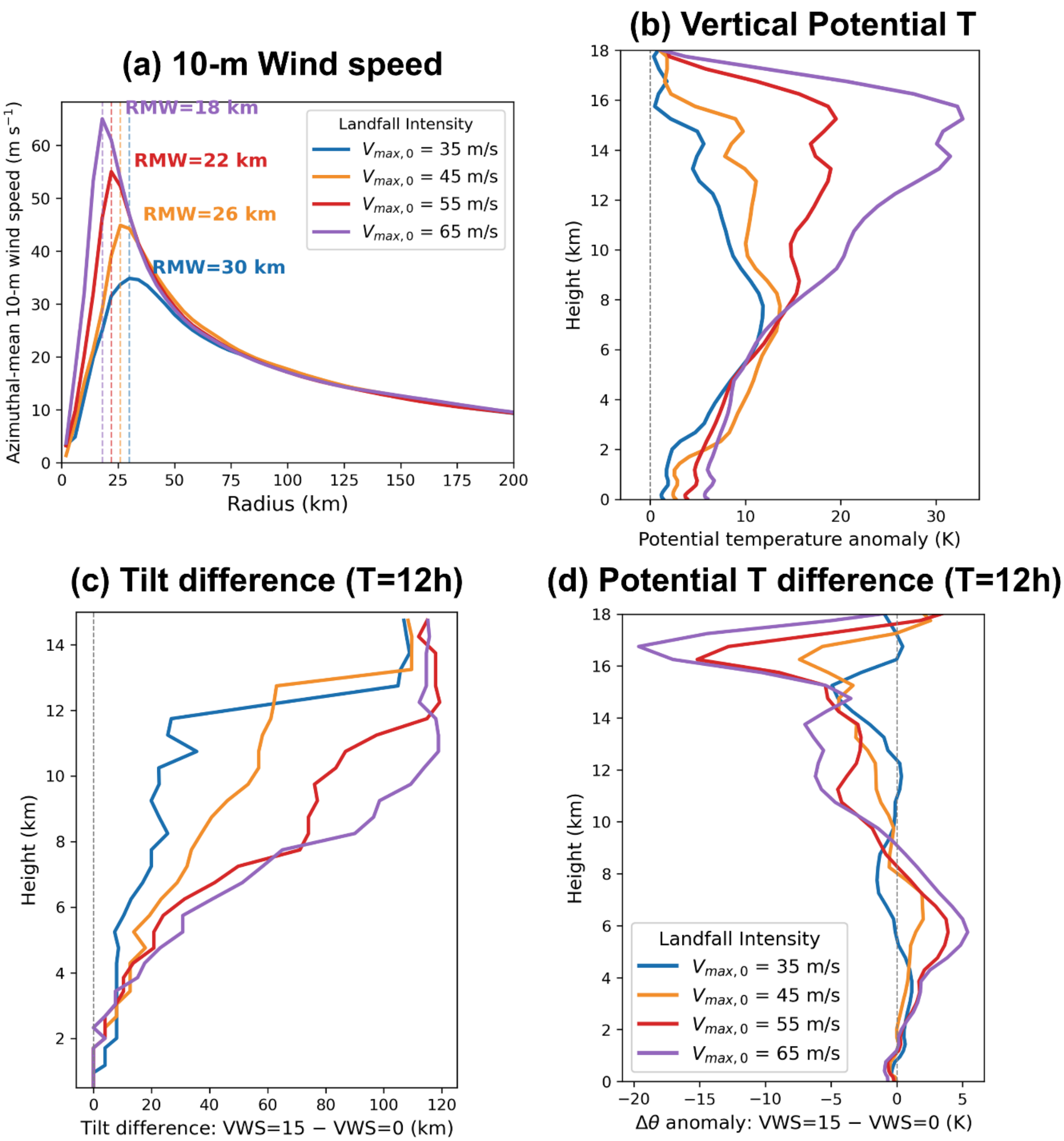


**Supplementary Fig. 12. (a)** Azimuthally averaged 10-m wind speed as a function of radius from the tropical cyclone center to 200km radius, for four landfall intensities, derived from the landfall hour ($T$ = 0 h) under vertical wind shear (VWS) = 0 and sea surface temperature (SST) = 301 K conditions. Dashed vertical lines indicate the radius of maximum wind (RMW) for each experiment. **(b)** Vertical profiles of potential temperature ($\theta$) anomaly at the tropical cyclone core at $T$ = 0 h, computed as the difference between the area-averaged potential temperature within 50 km of the tropical cyclone center and the azimuthal mean between 150 and 250 km radius. **(c)** Vertical profiles of vortex tilt difference at $T$ = 12 h, defined as the horizontal displacement of the pressure-minimum center at each vertical level relative to the surface center, computed as the difference between the VWS = 15 m/s and VWS = 0 m/s experiments for each landfall intensity group. Positive values indicate greater vortex tilt induced by VWS relative to the no-shear control. **(d)** As in **c**, but showing the vertical profile of difference ($\Delta\theta$) in potential temperature anomaly between the VWS = 15 m/s and VWS = 0 m/s experiments at $T$ = 12 h.

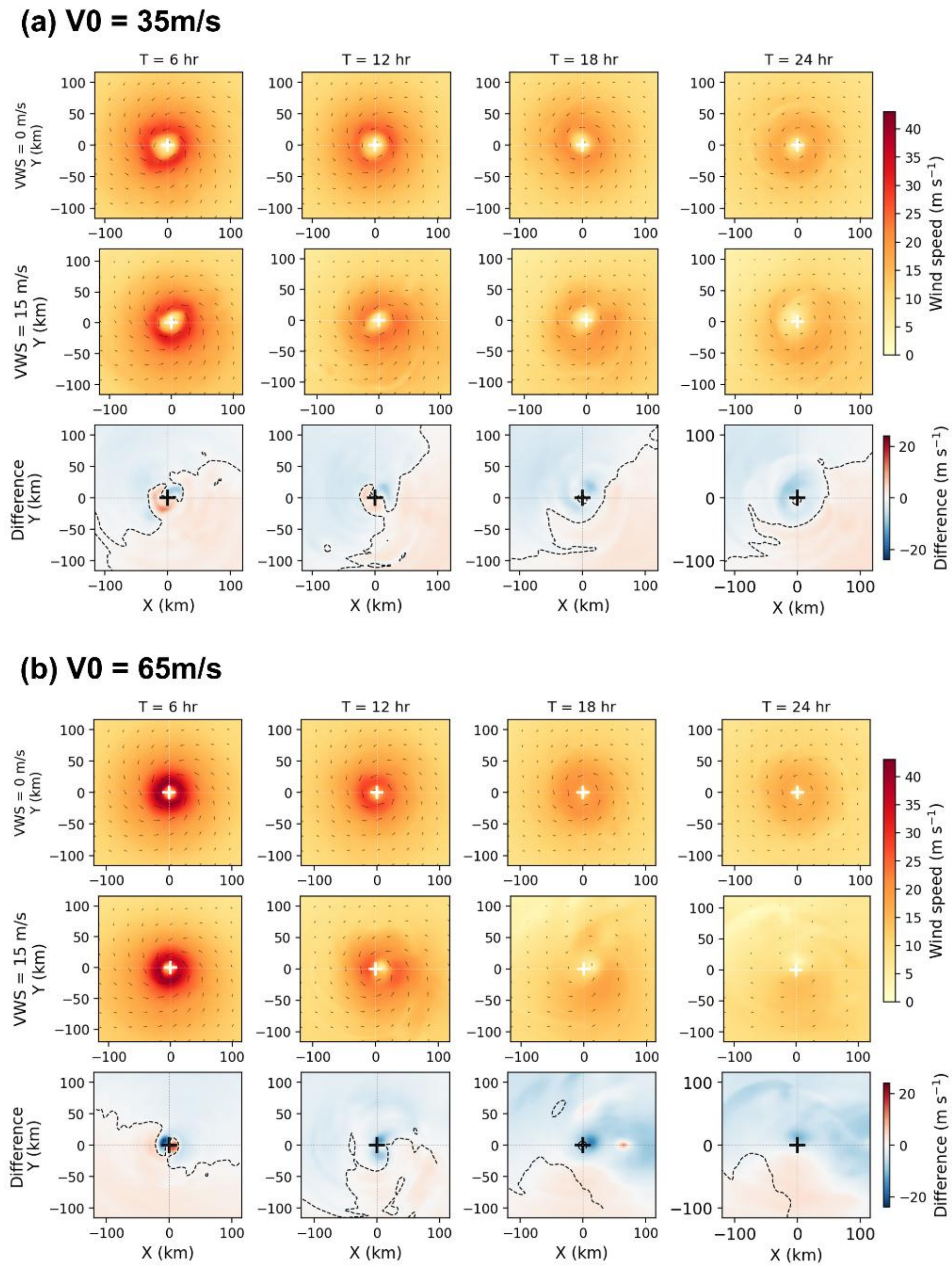


**Supplementary Fig. 13. (a)** Horizontal maps of 10-m wind speed (shading) and wind vectors (arrows) centered on the tropical cyclone surface pressure minimum (white cross) at $T = 6$, 12, 18, and 24 h after landfall, for the landfall intensity group landfall intensity ($V_0$) = 35 m/s. The top, middle, and bottom rows show the vertical wind speed (VWS) = 0 m/s experiment, the VWS = 15 m/s experiment, and their difference (VWS = 15 minus VWS = 0), respectively. In the difference panels, positive (red) and negative (blue) values indicate regions where wind speed is greater and smaller, respectively, under VWS = 15 m/s relative to the no-shear control; the black cross marks the tropical cyclone center and the dashed contour indicate the zero-difference line. **(b)** As in **(a)**, but for the landfall intensity group $V_0 = 65$ m/s.

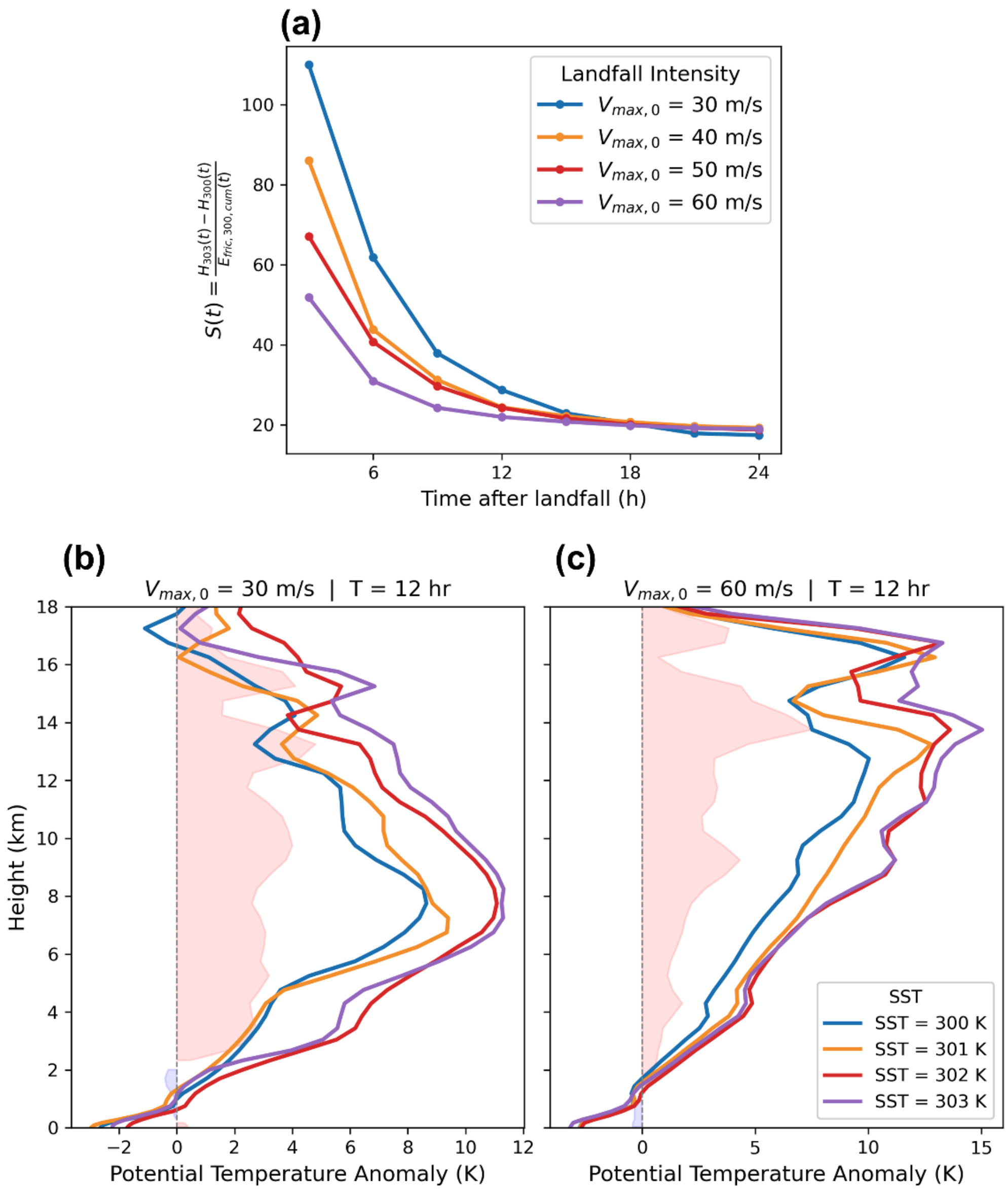


**Supplementary Fig. 14.** **(a)** Time series of the energy ratio $S(t) = [H_{303}(t) - H_{300}(t)]/E_{fric,300,cum}(t)$ after landfall for four landfall intensity groups, where $H_{303}(t)$ and $H_{300}(t)$ are the column-integrated moist static energy, J m$^{-2}$) averaged within 150 km of the tropical cyclone center at time $t$ for the sea surface temperature (SST) = 303 K and SST = 300 K experiments, respectively, and $E_{fric,300,cum}(t)$ is the cumulative surface friction dissipation (J m$^{-2}$) of the SST = 300 K experiment up to time t, also averaged within 150 km of the tropical cyclone center. The ratio S is dimensionless and quantifies the magnitude of SST-warming-induced thermal energy storage enhancement relative to the cumulative frictional energy consumption. (**b**, **c**), Vertical profiles of potential temperature anomaly at $T = 12$ h under SST = 300, 301, 302, and 303 K, for landfall intensities of **(b)** $V_0 = 30$ m/s and **(c)** $V_0 = 60$ m/s. The anomaly is computed as the difference between the area-averaged potential temperature within 50 km of the tropical cyclone center and the azimuthal mean between 150 and 250 km radius. The red shading indicates the region where the SST = 303 K profile exceeds the SST = 300 K profile.

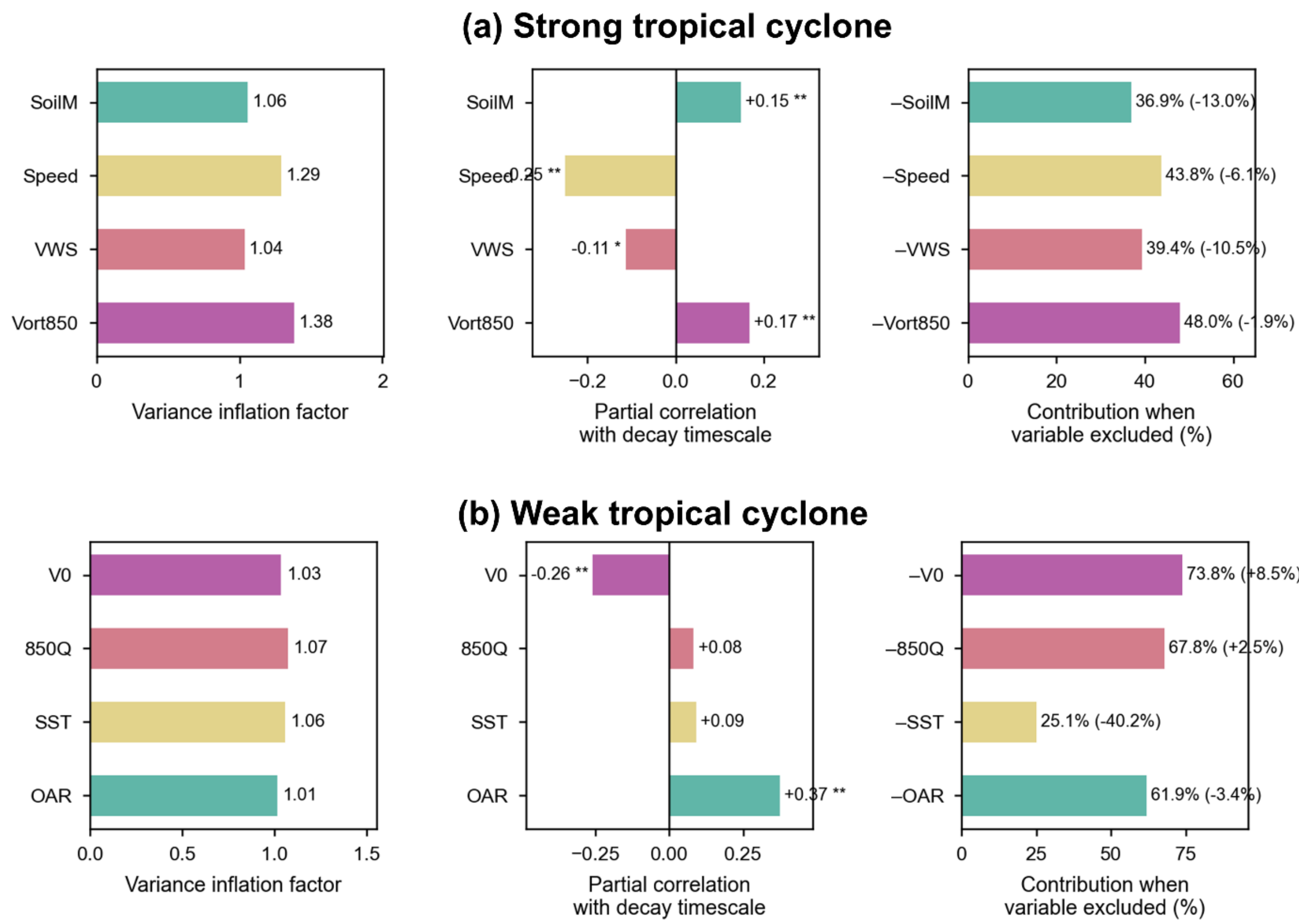


**Supplementary Fig. 15. (a)** Left panel: Variance inflation factor (VIF) for each predictor variable in the OLS regression model for strong tropical cyclones. VIF values quantify the degree of multicollinearity among predictors, with values substantially above 1 indicating potential collinearity. Mid panel: Partial correlations between each predictor and the decay timescale for strong tropical cyclones, computed by regressing out the influence of all other predictors from both the variable of interest and the target variable before computing the Pearson correlation coefficient. Asterisks indicate statistical significance (* $P < 0.05$, ** $P < 0.01$). Right panel: Leave-one-out (LOO) sensitivity analysis showing the total explained contribution (%) of the remaining predictors when each variable is sequentially excluded from the OLS regression. Values represent the percentage of the observed decay timescale trend explained by the reduced predictor set, with the change relative to the full-model contribution given in parentheses. **(b)** Same as **(a)** except for weak tropical cyclones. Soil moisture (SoilM), translation speed (Speed), vertical wind shear (VWS), and 850hPa vorticity (850Vor) for strong tropical cyclones; landfall intensity (V0), 850hPa specific humidity anomaly (850Q), sea surface temperature anomaly (SST), and ocean area ratio (OAR) for weak tropical cyclones.

**Supplementary Table. 1.** Tropical cyclone (TC) precipitation difference between early and late period for strong and weak events over coastal (0–50km from coastline) and inland (50–500km from coastline) for each basin.

| Strong TC precipitation difference (mm/event) | Coastal (0–50km) | Inland (50–500km) | Weak TC precipitation difference (mm/event) | Coastal (0–50km) | Inland (50–500km) |
|---|---|---|---|---|---|
| NA | 16.17 (51.4%) | -0.83 (-4.2%) | NA | -3.90 (-13.0%) | 3.58 (22.5%) |
| EP | 44.47 (168.3%) | -1.46 (-13.1%) | EP | 3.22 (7.9%) | 2.85 (26.6%) |
| NI | 16.20 (45.8%) | 3.29 (21.6%) | NI | -17.19 (-28.8%) | 0.06 (0.3%) |
| SP | 15.38 (39.8%) | 6.13 (51.2%) | SP | -3.23 (-7.9%) | -1.10 (-7.2%) |
| WP | 5.47 (11.1%) | -1.15 (-5.1%) | WP | -3.83 (-8.2%) | 2.29 (11.5%) |
| SI | 14.27 (50.4%) | -6.35 (-27.2%) | SI | 8.74 (63.8%) | -6.15 (-27.7%) |

**Supplementary Table. 2.** Key factors in three categories.

| TC Characteristics | Thermodynamic | Dynamic |
|---|---|---|
| Landfall intensity (m/s) (10-m maximum wind speed at landfall hour) | Sea Surface Temperature Anomaly (K) (SST anomaly relative to the 1979–2024 climatological mean at landfall location) | 850hPa Vorticity (1/s) (850hPa Vorticity at landfall location) |
| Translation speed (km/h) (Distance traveled within 24 hours after landfall) | 850hPa Specific Humidity Anomaly (g/kg) (850hPa Q anomaly relative to the climatological mean at landfall location) | 500hPa Vertical Wind Speed (m/s) (500hPa vertical wind speed at landfall location) |
| Ocean Area Ratio (Proportion of ocean area within a 150 km radius within 24 hours after landfall) | Soil Moisture Anomaly ($m^3/m^3$) (Soil Q anomaly relative to the climatological mean at landfall location) | Vertical Wind Shear (m/s) (Vector wind difference between 200hPa and 850hPa at landfall location) |
| Topographic height (m) (Mean topographic height within a 150 km radius within 24 hours after landfall) | | |

**Supplementary Table. 3.** Parameters for CM1 model simulations.

| Model Items | Setting |
|---|---|
| Resolution | dx, dy = 3km, dz = 500m |
| Spatial grid | 384 (x) × 384 (y) × 59 (z) |
| Turbulence model | Smagorinsky-type model; the mixing length in the (x, y) plane, $l_h$, increases from 100 m to 1 km as the underlying surface pressure decreases from 1015 to 900 hPa; the mixing length in the z direction, $l_v$, increases from 10 m to 100 m as z increases. |
| Boundary conditions | Top and lateral: full slip.<br>Bottom: $C_d$ varies with V; for $V \leq 5$ m/s, $C_d = 0.001$; for $5$ m/s $\leq V \leq 25$ m/s, $C_d$ linearly increases from 0.001 to 0.0024; for $V > 25$ m/s, $C_D$ = 0.0024[1,2]; $C_e = 0.0012$[3] over ocean and $C_e = 0$ over land. |
| Sea-surface temperature (SST) | 300K, 301K, 302K, and 303K |
| Vertical Wind Shear (VWS) | 0 m/s, 5 m/s, 10 m/s, 15 m/s |
| Coriolis force | 0.00005 $s^{-1}$ |
| Moving domain | i_move = 1 for VWS > 0 |
| Sounding | “Dunion_MT sounding”[4], a benchmark sounding for hurricane simulations in CM1[5,6,7]. |

**Supplementary References**


1. Fairall, C., Bradley, E. F., Hare, J., Grachev, A. & Edson, J. Bulk parameterization of air-sea fluxes: updates and verification for the COARE algorithm. J. Clim. 16, 571–591 (2003).
2. Donelan, M. et al. On the limiting aerodynamic roughness of the ocean in very strong winds. Geophys. Res. Lett. 31, L18306 (2004).
3. Drennan, W. M., Zhang, J. A., French, J. R., McCormick, C. & Black, P. G. Turbulent fluxes in the hurricane boundary layer. Part II: Latent heat flux. J. Atmos. Sci. 64, 1103–1115 (2007).
4. Dunion, J. P. Rewriting the climatology of the tropical North Atlantic and Caribbean Sea atmosphere. J. Clim. 24, 893–908 (2011).
5. Bryan, G. H. & Fritsch, J. M. A benchmark simulation for moist nonhydrostatic numerical models. Mon. Weath. Rev. 130, 2917–2928 (2002).
6. Bryan, G. H. & Rotunno, R. The maximum intensity of tropical cyclones in axisymmetric numerical model simulations. Mon. Weath. Rev. 137, 1770–1789 (2009).
7. Bryan, G. H. Effects of surface exchange coefficients and turbulence length scales on the intensity and structure of numerically simulated hurricanes. Mon. Weath. Rev. 140, 1125–1143 (2012).
