# Supplementary material for: Divergent trends in post-landfall decay of strong and weak tropical cyclones
Source: Nat Commun. 2026 Sep 24;17:11099. doi: 10.1038/s41467-026-77958-3 (PMC13696725; doi:10.1038/s41467-026-77958-3)
Supplement: Supplementary file 2 [file 41467_2026_77958_MOESM2_ESM.pdf]

nature portfolio

Peer Review File

Divergent Trends in Post-landfall Decay of Strong and Weak Tropical Cyclones

Corresponding Author: Professor Zong-Liang Yang

This file contains all reviewer reports in order by version, followed by all author rebuttals in order by version.

Version 0:

Reviewer comments:

Reviewer #1

(Remarks to the Author)
Summary: This study proposed an intensity-dependent bifurcation in global trends of landfalling tropical cyclone (TC) decay rate, i.e., intense TCs decay faster, while weak TCs tend to persist longer over land. These contrasting trends were attributed to the differences in structure and response between intense and weak TCs based on a machine-learning analysis: intense TCs are more sensitive to the dynamic conditions and loss their intensity rapidly when TCs collapse after landfall, but weak TCs are easily to be persist if there are enough warm moisture supply. The insight proposed in this study is interesting, however, the analysis should be strengthened, both in trend analysis and the physical mechanism analysis. Some conclusions were made by speculating without rigorous analysis and validation. With these concerns, I would recommend to reject the publication of this manuscript in Nature Communications.

#1 The trend analysis should be strengthened by using more observations from different institutions, longer observed period, and also multi-model ensembles, given the inhomogeneity, and large uncertainty of recorded TC intensity.

Fig 2: The inner-year variability should be given. Both decay timescale and landfall latitude show great interdecadal variations, even in a very short study period of 35 years. Are these results sensitive to the selection of threshold of strong and weak TCs?

Line 115-116: I may not agree with the claim of continuous decreasing trend of strong TC decay timescale, since there is an obvious peak value around 1998. Also, a valley value occurred in 2003 for decay timescale of weak TCs.

Line 131-135: Still, the ocean area ratio shows large inter-decadal variation in a short period. The ocean area ratio surged from 1990 to ~2007, and then decreased sharply, which makes the trend less reliable. I would suggest to use a longer study period or employ the multi-model ensemble analysis to increase the reliable of this analysis.

Line 137-140, and Line 253-254: The 855 ERA5-driven synthetic TCs in a one of six basins are not enough for validating the observed trends of 35 years, given the large inter-decadal variations.

Line 139-140, and Line 253-254: The conclusion was drawn without any rigorous climate change attribution analysis but only a comparison of observation and ERA5 data of 35 years.

#2 The physical mechanism analysis should be strengthened by providing some necessary evidence aside from the results of machine learning. For example, the observed structural differences between strong and weak TCs, and the evidence on different responses of strong and weak TCs to dynamic and thermodynamic factors.

Line 217-219: Why detrend the temperature? I am wondering the reasonability of drawing the limited predictive importance of temperature to TCs from the weak correlation after detrend. Since it is well known that the temperature, especially SST, has significant impact on TC activity, and is one of the most important predictors of TC activity (i.e., Elsner et al., 2008; Knutson et al., 2010; Li & Chakraborty, 2020).

Line 227: Both strong and weak tropical cyclones have experienced decreased soil moisture.

Line 232: The statement of “regions experiencing moistening trends” needs support.

Line 233-234: I don’t understand why dryer soil would responsible to the more frequent strong tropical cyclones in SP? If the “drying” means less precipitation, then why more frequent strong tropical cyclones cause less precipitation. Even though tropical cyclones are one important trigger of precipitation, it is not the only system that produces precipitation.

Figure 4: I would suggest replacing Figs 4b and 4c using Extended Data Fig 8, which provide more detailed information, or maybe consider to combine these figures.

Extended Data Table 3: Even though the authors claimed that the thermodynamic factors, particular the low-level moisture, are important drivers of slower decay of weak TCs, the correlation between these factors and weak TC decay timescale are not very high, and did not show obvious differences compared to that between strong TCs.

References:
Elsner, J. B., Kossin, J. P., & Jagger, T. H. (2008). The increasing intensity of the strongest tropical cyclones. Nature, 455(7209), 92–95. https://doi.org/10.1038/nature07234
Knutson, T. R., McBride, J. L., Chan, J., Emanuel, K., Holland, G., Landsea, C., Held, I., Kossin, J. P., Srivastava, A. K., & Sugi, M. (2010). Tropical cyclones and climate change. Nature Geoscience, 3(3), 157–163. https://doi.org/10.1038/ngeo779
Li, L., & Chakraborty, P. (2020). Slower decay of landfalling hurricanes in a warming world. Nature, 587(7833), 230–234. https://doi.org/10.1038/s41586-020-2867-7

Others:

Line 45: ref #17 did not support the conclusion about decreasing cyclone durations from poleward shifts, please check.

Line 83: This conclusion should be made with cautious. In Lai et al. (2024) (also ref #10 in this study), the relationship between TC decay timescale and landfall intensity was also examined, which shows a significant ($p = 0.06$) positive correlation between them, i.e., stronger landfall intensity yields larger decay timescale (slower decay), which is the opposite of this study. One of the possible reasons causing the opposite results is that when the landfall intensity <25m/s, the decay timescale decreases sharply as landfall intensity increasing, while such a relationship does not exist for TCs with landfall intensity >25 m/s (Noted that analysis of Lai et al. (2024) was based on TCs with landfall intensity >24.5 m/s).

Line 106-109: I have doubt on this statement, since the upper bound and lower bound are envelope bounds of all TCs, rather than the decay curve of any TC. For an extreme example (which may be not true), one strong storm (upper bound) can decay very fast and reach lower bound. May consider to show more detailed decay curves.

Fig 3 and Extended Data Fig. 7: The statistic significant level of differences between periods were provided, however, what are the differences refer to? differences of rainfall/PDI between periods, or the decay rate of rainfall/PDI between periods?

Fig 3: I would suggest to revise these maps: The 3D rotation is not necessary, which will not provide any more information, but makes it harder to identify the geographical location. Further, it is difficult to distinguish the colors (and the indicated values) in rotated views. It is hard to tell, at what distance, how much the difference in precipitation rate is.

(Remarks on code availability)
The code and relative dataset provided are organized well.

Reviewer #2

(Remarks to the Author)
This study explores the post-landfall decay of strong and weak TCs. It is interesting to find that the strong storms now weaken 22% faster after landfall and weak storms persist 19% longer inland in recent 35 years. Then analyzed the environmental influencing factors using machine learning methods. The article has innovative findings, but lacks appropriate in-depth analysis. Overall, this study holds important practical significance. However, there are some major issues that need to be addressed.

1.In your study, you choose decay timescale as the indicator to analyze the post-landfall decay of strong and weak TCs. You mentioned that “larger decay timescale indicates slower decay”. Why don't you just use the intensity decay rate (intensity change after landing divided by the duration after landing) of landfalling TCs to analyze? Are the results of intensity decay rate similar to the decay timescale?
2.Please clarify the locations of strong and weak TCs in Figure 2a
3.In line 118-120: You mentioned that “Independent validations using time-to-decay to tropical depression (11 m s^{-1}) confirms these opposing trends (Extended Data Fig. 2), indicating robust intensity-dependent changes in tropical cyclone decay rate.” the meaning of “time-to-decay” that I understand is the duration of post-landfall TCs. However, time-to-decay cannot accurately represent the intensity decay rate of ladnfalling TCs. Because different landfall intensity can cause different decay rates after landfall although the time-to-decay is same. Therefore, I don't think Extended Data Fig. 2 can fully support your above conclusion. Why not use the intensity decay rate directly?
4.Suggest adding some mechanism analysis of environmental factors to analyze the reasons for the different intensity decay of strong and weak typhoons. The entire article only has statistical analysis and lacks appropriate mechanism analysis.

5.Many studies (e.g. Wu et al. 2005, Tu et al. 2009, Park et al. 2011, Liu et al. 2020; Zhang et al. 2023) have found that the landfall latitude of TCs in the Western Pacific (WP) tend to move northward as a whole, but your statistical results show that the landfall latitude tend to move southward (Extended Data Fig. 4f). Can you explain why your results are contrary to previous work?
Zhang, L., Cheng, T. F., Lu, M., Xiong, R., & Gan, J. (2023). Tropical cyclone stalling shifts northward and brings increasing flood risks to East Asian Coast. Geophysical Research Letters, 50(10), e2022GL102509.
Wu, L., B. Wang, and S. Geng, 2005: Growing typhoon influence on East Asia. Geophys. Res. Lett., 32, L18703, https://doi.org/
10.1029/2005GL022937.
Tu, J. Y., C. Chou, and P.-S. Chu, 2009: The abrupt shift of typhoon activity in the vicinity of Taiwan and its association with western North Pacific–East Asian climate change. J. Climate, 22, 3617–3628, https://doi.org/10.1175/2009JCLI2411.1.
Park,D.-S. R., C.-H.Ho, J.-H. Kim, and H.-S. Kim, 2011: Strong landfall typhoons in Korea and Japan in a recent decade. J. Geophys. Res., 116, D07105, https://doi.org/10.1029/2010JD014801.
Liu, L., Wang, Y., Zhan, R., Xu, J., & Duan, Y. (2020). Increasing destructive potential of landfalling tropical cyclones over China. Journal of Climate, 33: 3731-3743.
6.Please explain the meaning of “ocean area ratio” and how to calculated it. It is averaged from landfall to TD? It is related to TC size? When does a TC make landfall start counting? Is the calculation starting from the center of the typhoon crossing the coastline? It will affect the ocean area ratio.
7.In line 126-130: please explain the relationship between decay rate of weak and strong TCs and the landfall latitude. Why strong TC equatorward shirt is favorable to intensity decay? The lines of 131-136 cannot explain the different decay of weak and strong TCs incent decades. Suggest analyzing the underlying surface, environmental field, and characteristics of the typhoon itself to identify the reasons for this phenomenon.
8.The distribution of strong and weak typhoons in Extended Data Fig. 7 does not correspond?

9.Minor
Figure 3 (in line 183-184): (a,b) change to (c,d)

(Remarks on code availability)

Reviewer #3

(Remarks to the Author)
This manuscript investigates the opposing trends in post-landfall decay of strong and weak tropical cyclones. Analysing 506 events from 1990–2024, the authors show that strong storms exhibit a smaller decay timescale, while weak storms show a longer decay timescale. The study highlights the non-uniform response of post-landfall decay under climate change. The topic is relevant to tropical cyclone post-landfall risk, and the manuscript is generally well structured. I have few questions regarding the physical explanation behind the reported trends. Please see my comments below.

Minor comments:
Discussion (Line 260-269):
The opposing trends are related to structural differences between strong and weak storms. However, it is not clear why strong storms show a decreasing trend in decay timescale, while weak storms show an increasing trend (Fig. 2c). The authors suggest that contrasting climate regimes for weak and strong storms are responsible for these trends, yet TC characteristics still appear to be the dominant drivers (Fig. 4). Could this be related to strong storms becoming stronger while weak storms become weaker under climate change?

Introduction:
While the authors review studies emphasizing the role of thermodynamics (e.g., SST and moisture) in tropical cyclone behaviour, it may be helpful to also include some context of the dynamical controls on tropical cyclones, especially since the manuscript compares TC sensitivities to thermodynamic and dynamical factors.

Line 79-80: Should “decay rate” refer to “decay timescale”? It may be helpful to use consistent terminology throughout the manuscript.
Line 141: The purpose of Fig. 2a is not entirely clear to me.
Line 183: (a,b)->(c,d)

(Remarks on code availability)

Version 1:

Reviewer comments:

Reviewer #1

(Remarks to the Author)
I have carefully read the authors’ point-by-point responses and examined the revised manuscript. I am pleased with the

thorough and thoughtful revisions. The authors have addressed all my concerns: they have substantially expanded the observational dataset (from 506 to 885 landfalling events), extended the analysis period to 1979-2024, added multi-institution data, and performed sensitivity tests on thresholds and time splits. And most importantly, it provided robust mechanistic support through correlation analyses, environmental trend decomposition, and idealized numerical model experiments (CM1). The revised manuscript has been greatly improved in statistically robustness and physically coherence. I would like to support the publication of this study in Nature Communications after revising the following suggestions:

Minor comments:

1. In the revised Fig. 2a, the time series for strong and weak TCs are now shown separately. Please ensure that the caption clearly defines what the solid/dashed lines and blue/red shadings represent (e.g., standard error or standard deviation).

2. In the manuscript, both “Ocean Area Ratio” and “ocean area ratio” were used. Please maintain consistency in terminology and check other similar places. Besides, please incorporate “OAR” and the citation (i.e., Chan et al., 2022) into the Section Materials and Methods – Environmental factors, making it easy for readers to find the OAR.

3. I appreciate that you have removed the potentially misleading detrending analysis and now clearly state that SST warming slows the decay for all storms, but its benefit is overwhelmed by other unfavorable factors for strong TCs. Please ensure that no ambiguous wording about the role of SST remains in the final text.

4. For CM1 experiments, they use fixed SST and no background flow, which simplifies reality. I would appreciate a sentence acknowledging that these experiments isolate specific mechanisms, but that real-world interactions (e.g., with soil moisture gradients or coastal geometry) may further modulate the response.

5. It is suggested to carefully reviewing all maps shown in the figures, particularly national borders.

(Remarks on code availability)
Overall, the authors have provided the code used to generate figures from processed or analyzed data (results). However, this code does not reveal the steps involved in processing and analyzing the public accessible data to arrive at the reported results, which limits the reproducibility of the study.

Reviewer #2

(Remarks to the Author)
The revised paper is an improvement and can be accepted with some revisions.
1. You divide TCs into strong TCs and weak TCs. And you show that strong storms weaken faster after landfall, whereas weak storms persist 14.5% longer inland. Is this related to the annual changes in TC intensity? Has the strong TC been strengthening year by year and the weak TC been weakening year by year?
2. Your intensity changes are considered within 24 hours after landfall, but the TC precipitation is considered within 72 hours after landfall? Suggest that these two times should be consistent.
3. In your response, you mentioned that decay timescale (τ) was chosen as the primary metric because it normalizes for the strong dependence of absolute intensity loss on landfall intensity. In table 1, why is it still significant between weak TCs and landfall intensity after removing the impact of landfall intensity?
4. In figure 2d, you analyzed the characteristics of the landfall latitude, but I didn't see the necessity of analyzing landfall latitude in the article. Suggest adding some description and discussion.

(Remarks on code availability)
The code and relative dataset provided are organized well.

Reviewer #3

(Remarks to the Author)
Regarding my major concern about the driving causes behind the diverging trends between strong and weak storms, I am still not fully convinced.

First, I understand that the analysis is based on grouped strong and weak storms. However, I do not think this necessarily rules out the influence of trends in storm intensity itself. Potential changes in TC intensity and structure could be important factors contributing to the observed decay trends.

Second, while the sensitivity analysis is informative, I do not think it necessarily establishes a cause-and-effect relationship.

Third, I was wondering whether you have examined any trends in the observed VWS.

Overall, I remain concerned that the mechanism underlying the observed trends has not yet been fully explained.

(Remarks on code availability)

Version 2:

Reviewer comments:

Reviewer #2

(Remarks to the Author)
The authors have addressed and explained all my previous concerns. I have no further comments.

(Remarks on code availability)

Reviewer #3

(Remarks to the Author)
I am pleased with the revisions.

(Remarks on code availability)

Open Access This Peer Review File is licensed under a Creative Commons Attribution 4.0 International License, which permits use, sharing, adaptation, distribution and reproduction in any medium or format, as long as you give appropriate credit to the original author(s) and the source, provide a link to the Creative Commons license, and indicate if changes were made.
In cases where reviewers are anonymous, credit should be given to 'Anonymous Referee' and the source.
The images or other third party material in this Peer Review File are included in the article's Creative Commons license, unless indicated otherwise in a credit line to the material. If material is not included in the article's Creative Commons license and your intended use is not permitted by statutory regulation or exceeds the permitted use, you will need to obtain permission directly from the copyright holder.
To view a copy of this license, visit https://creativecommons.org/licenses/by/4.0/

We thank you for your thoughtful and constructive comments, which have substantially strengthened our work. We have carefully revised the manuscript to address each of your comments and concerns by expanding the dataset from multiple operational meteorological agencies, extending the analysis period to 1979–2024, increasing the sample from 506 to 885, adding more analysis, and conducting idealized numerical model experiments with sensitivity tests. Below, we respond point-by-point to your comments (shown in blue), with our responses in black and additions to the manuscript highlighted in red.

Response to Reviewer 1

Summary: This study proposed an intensity-dependent bifurcation in global trends of landfalling tropical cyclone (TC) decay rate, i.e., intense TCs decay faster, while weak TCs tend to persist longer over land. These contrasting trends were attributed to the differences in structure and response between intense and weak TCs based on a machine-learning analysis: intense TCs are more sensitive to the dynamic conditions and loss their intensity rapidly when TCs collapse after landfall, but weak TCs are easily to be persist if there are enough warm moisture supply. The insight proposed in this study is interesting, however, the analysis should be strengthened, both in trend analysis and the physical mechanism analysis. Some conclusions were made by speculating without rigorous analysis and validation. With these concerns, I would recommend to reject the publication of this manuscript in Nature Communications.

#1 The trend analysis should be strengthened by using more observations from different institutions, longer observed period, and also multi-model ensembles, given the inhomogeneity, and large uncertainty of recorded TC intensity.
We appreciate your constructive suggestions. We understand and agree that the quality of observational data directly determines the robustness of our conclusions. Therefore, we have expanded the global observations to include more operational meteorological agencies across six basins, extending the analysis period from 1990–2024 to 1979–2024 and increasing the sample from 506 to 885 global landfalling tropical cyclones.

The expanded dataset confirms and strengthens the robustness of major conclusions: (1) the intensity-dependent bifurcation between strong and weak tropical cyclone decay (which is particularly robust in the post-2000 period); (2) Global analysis identified an intensity-dependent bifurcation in global trends of tropical cyclone post-landfall decay, centered near 29 m s^{-1} (10-min maximum wind speed) at landfall intensity. The previous threshold 33 m s^{-1} is based on 1-min maximum wind speed that equals to 29.04 m s^{-1} (33×0.88; Harper et al., 2010) in 10-min maximum wind speed, strongly support the robustness of the intensity-dependent bifurcation; (3) The physical mechanisms are also robust: the divergent changes are physically coherent with the key environmental conditions. Please find further details in

our response below.

Moreover, while climate model ensembles may offer a useful perspective, their coarse resolution cannot resolve the complex sub-grid-scale processes governing post-landfall decay, and inter-model spread in key factors (e.g., soil moisture, atmospheric stability) largely reflects structural model bias rather than true climate signals. Accordingly, the expanded observational dataset provides a more robust basis for our conclusions.

The revised manuscript is attached below for your reference:

"To build a robust, globally consistent dataset, we incorporated data from multiple agencies across six ocean basins: the U.S. best-track agencies, including the National Hurricane Center (NHC; HURDAT2), Central Pacific Hurricane Center (CPHC), and Joint Typhoon Warning Center (JTWC), provide basin-of-record data for all basins, supplemented by the India Meteorological Department (IMD) for the North Indian Ocean, Météo-France La Réunion (RSMC La Réunion) for the South-West Indian Ocean, the Australian Bureau of Meteorology (BoM) for the South Pacific, and the China Meteorological Administration (CMA) and Hong Kong Observatory (HKO) alongside the Japan Meteorological Agency (JMA) for the Western Pacific.

We analyzed 885 global landfalling tropical cyclones from 1979 to 2024."

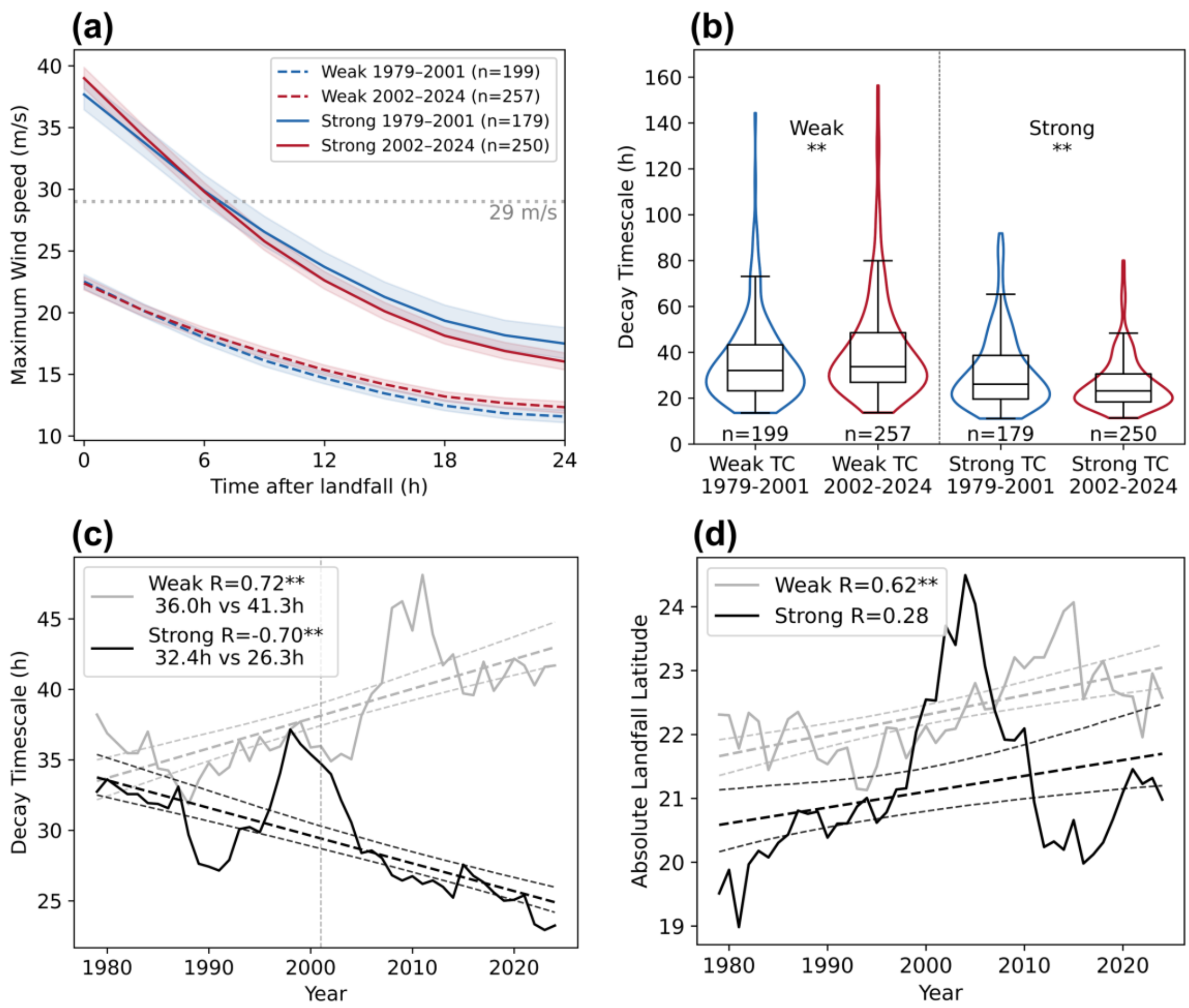

Fig. 2. Intensity-dependent changes in tropical cyclone post-landfall decay from 1979 – 2024. ***(a)*** *Time series of post-landfall wind speed for strong (solid line) and weak (dashed line) tropical cyclones, showing early (blue, 1979–2001) versus late (red, 2002–2024) period means with variability (shaded).* ***(b)*** *Distribution of decay timescales across intensity categories and periods. Violin plots combine probability density with box plots; sample sizes indicated as "n". Asterisks denote significant differences between periods (**$P < 0.01$). Temporal trends in* ***(c)*** *mean decay timescale and* ***(d)*** *mean absolute landfall latitude indicating poleward migration patterns, for strong and weak tropical cyclones using five-year running means. Trend coefficients, 95% CI, and significance levels are shown in* ***c*** *and* ***d****.*

Fig 2: The inner-year variability should be given. Both decay timescale and landfall latitude show great interdecadal variations, even in a very short study period of 35 years. Are these results sensitive to the selection of threshold of strong and weak TCs?

We thank you for raising this important point. The interannual variability in decay timescale is physically meaningful and may capture the variability related with large-scale climate effects such as ENSO. We have clarified this in the revised manuscript with Extended Data Fig. 3. Moreover, we conducted sensitivity tests using alternative thresholds of 28 and 30 m s^{-1} in addition to the data-driven threshold of 29 m s^{-1}, as well as alternative temporal period divisions (Extended Data Text. 1 and Extended Data Fig. 5). The key conclusions are reproduced consistently across all threshold and period choices.

Extended Data Fig. 3a presents the unsmoothed annual time series, which clearly shows the divergence in long-term tendencies between strong and weak tropical cyclones despite large year-to-year fluctuations. Such variability is inherent to tropical cyclone analysis given the limited annual sample sizes, the diversity of landfall tracks and environments, data quality inhomogeneities, and the influence of natural variability. The trend of decay timescale documented in relevant literature also shows strong inner-year variability after smoothed (Li and Chakraborty, 2020; Lai et al., 2024). Importantly, we demonstrate that this interannual variability is highly related to ENSO modulation. Decay timescales are much larger during La Niña years compared to El Niño and neutral years, especially for weak storms (Extended Data Fig. 3b-h) across most basins. This ENSO signal explains many of the prominent peaks and troughs visible in the annual time series, and does not undermine the long-term divergence between strong and weak tropical cyclones, which is our central finding.

"Such variability is expected given the limited annual sample sizes and the complex interaction between environmental factors and post-landfall decay, consistent with previous studies[9,10]. Natural variability contributes substantially to these fluctuations, many peak values coincide with La Niña years (Extended Data Fig. 3a), and weak tropical cyclone decay timescales are significantly larger during La Niña years compared to El Niño and neutral years (Extended Data Fig. 3b and c), with a similar but weaker pattern for strong tropical

cyclones. Nevertheless, the intensity-dependent bifurcation is robust, especially in the post-2000 period."

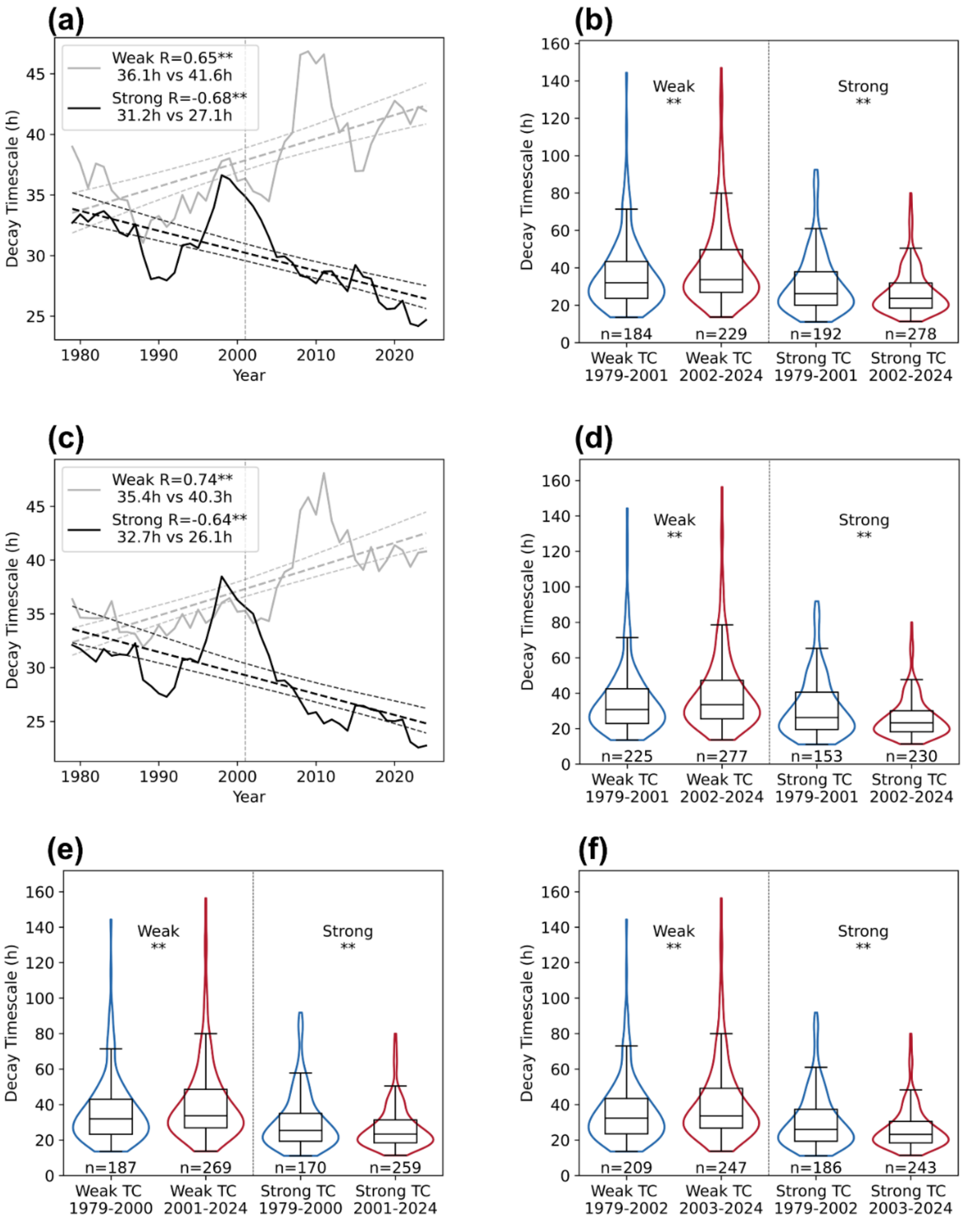

Extended Data Fig. 5. ***(a)*** *Temporal trends in mean decay timescale, for strong and weak tropical cyclones (threshold=28 m s⁻¹) using five-year running means. Trend coefficients, 95% CI, and significance levels are shown.* ***(b)*** *Distribution of decay timescales across intensity categories and periods (threshold=28 m s⁻¹). Violin plots combine probability density with box plots; sample sizes indicated as "n". Asterisks denote significant differences between periods (**P < 0.01).* ***(c), (d)*** *same as* ***(a), (b)*** *except for threshold=30 m s⁻¹.* ***(e), (f)*** *same as* ***Fig. 2b*** *except for alternative temporal splits: 1979 – 2000 versus 2001 – 2024, and 1979 –*

2002 versus 2003 －2024.

Line 115-116: I may not agree with the claim of continuous decreasing trend of strong TC decay timescale, since there is an obvious peak value around 1998. Also, a valley value occurred in 2003 for decay timescale of weak TCs.

We thank you for this careful reading. Although natural variability influences performance in certain years, the overall trends are statistically significant. We further investigated the 1998 and 2003 events and found that these two abnormal pattern could be explained with ENSO: The elevated strong tropical cyclone decay timescale around 1998 coincides with consecutive La Niña years during this period, which are associated with higher soil moisture, lower translation speeds, weaker VWS and higher vorticity at landfall locations (Extended Data Fig. 8a, b, c). Following this peak, strong tropical cyclone decay timescale entered a pronounced and sustained decline as these favorable conditions gave way to the soil drying, faster speed, and atmospheric stabilization trends documented in Fig. 4 and Extended Data Fig. 8–9.

Conversely, the valley in weak tropical cyclone decay timescale around 2003 coincides with four continuous El Niño and Neutral years, during which low-level atmospheric moisture was suppressed (Extended Data Fig. 8f). These physically coherent explanations reinforce our central finding that the long-term divergence between strong and weak tropical cyclone decay timescales is a robust signal that emerges clearly from the underlying natural variability, particularly in recent decades, which is physically associated with their different environmental sensitivities.

To avoid potential misunderstanding, we have revised the manuscript as below to more accurately characterize the temporal evolution of both trends.

"Temporal trend analysis reveals opposing long-term changes in strong and weak tropical cyclone decay (Fig. 2c). Weak tropical cyclones show an overall increasing trend in decay timescale (0.21 h/yr, 95% CI: 0.15–0.27, $R = 0.72$, $P < 0.01$), while strong tropical cyclones exhibit a decreasing trend (−0.20 h/yr, 95% CI: −0.26 to −0.14, $R = 0.70$, $P < 0.01$). However, these trends are accompanied by substantial interannual to interdecadal variability: weak tropical cyclone decay timescale remained relatively stable from 1979 to approximately 2000 before rising sharply, then declined gradually after 2012; strong tropical cyclone decay timescale fluctuated considerably through the mid-1990s before entering a pronounced and sustained decline."

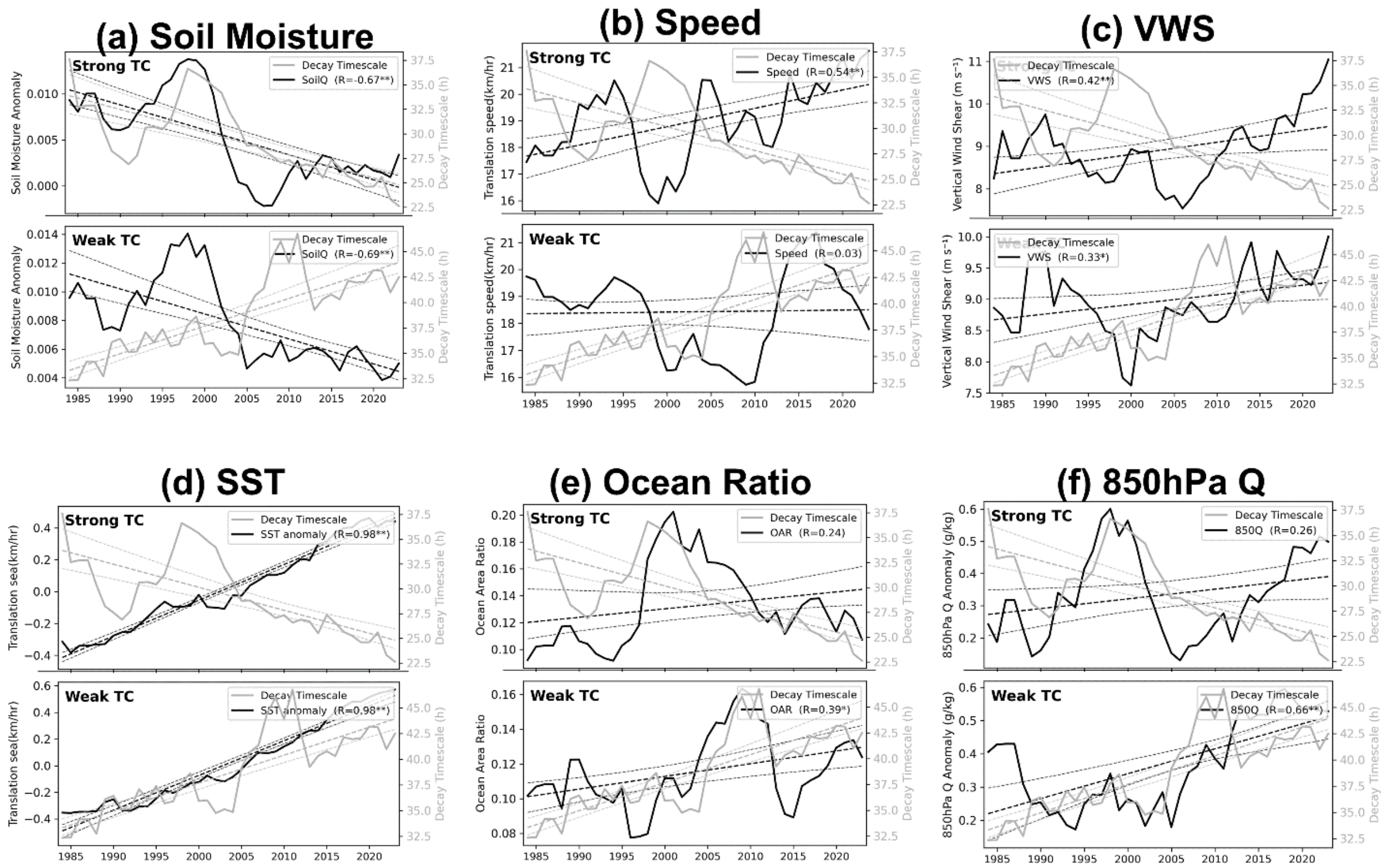

Extended Data Fig. 8. *Time series in key environmental variables combined with tropical cyclone decay timescale for strong and weak tropical cyclones. Each column shows the 5-year running mean time series of environmental variable (black line) alongside the decay timescale (gray line) for strong (upper sub-panel) and weak tropical cyclones (lower sub-panel):* ***(a)*** *soil moisture anomaly,* ***(b)*** *translation speed,* ***(c)*** *vertical wind shear,* ***(d)*** *sea surface temperature anomaly,* ***(e)*** *ocean area ratio, and* ***(f)*** *850-hPa specific humidity anomaly. Trend coefficients, 95% CI, and significance levels are shown.*

Line 131-135: Still, the ocean area ratio shows large inter-decadal variation in a short period. The ocean area ratio surged from 1990 to ~2007, and then decreased sharply, which makes the trend less reliable. I would suggest to use a longer study period or employ the multi-model ensemble analysis to increase the reliable of this analysis.

We appreciate your suggestions. The ocean area ratio (OAR) is largely determined by individual storm track geometry and landfall angle (Chan et al., 2022), which introduces substantial stochastic variability and makes a statistically robust long-term trend difficult to establish. In the revised analysis using the expanded dataset (885 events, 1979–2024), the OAR time series shows distinct behavior between intensity categories. For strong tropical cyclones, OAR exhibits a sharp increase followed by a decline after approximately 2000, a temporal evolution that is coherent with the accelerating decay timescale trend of strong storms during the same period. For weak tropical cyclones, OAR shows a more sustained

increasing tendency over the full study period, consistent with the prolonged persistence observed for weak storms (Extended Data Fig. 8e).

While we do not claim that OAR exhibits a statistically significant monotonic long-term trend, given the inherent stochasticity of individual track geometries, its co-variation with decay timescale across both intensity categories suggests a meaningful physical contribution. This is corroborated by the significant year-scale correlation between OAR and weak tropical cyclone decay timescale ($R = 0.66$, $P < 0.01$; Fig. 4e).

Line 137-140, and Line 253-254: The 855 ERA5-driven synthetic TCs in a one of six basins are not enough for validating the observed trends of 35 years, given the large inter-decadal variations.

Thank you for pointing out this. We agree that the RAFT synthetic dataset covers only the North Atlantic basin and therefore cannot serve as a global validation. Nevertheless, the RAFT results retain meaningful supporting value for several reasons. First, the NA is one of the two basins (alongside WP) where the intensity-dependent bifurcation is most pronounced in the observations, making it a particularly relevant test case. Second, the RAFT framework uses ERA5 reanalysis as its climate background, meaning that the reproduced bifurcation with a threshold of 29.4 m s^{-1} closely matching our observationally derived 29.1 m s^{-1} can support the robustness of intensity-dependent divergence. Third, our primary robustness of conclusions rests on the multi-agency observational analysis, with the RAFT results serving as one additional corroborating evidence.

We have revised the manuscript to describe the RAFT analysis as an independent support: "In addition, analysis of 1,014 synthetic North Atlantic tropical cyclones from the RAFT framework[45] also reveals a significant increase in weak tropical cyclone decay timescales and a significant decrease for strong tropical cyclones (threshold = 29.4 m s^{-1}), lending independent support to our observational findings (Extended Data Fig. 6)."

Line 139-140, and Line 253-254: The conclusion was drawn without any rigorous climate change attribution analysis but only a comparison of observation and ERA5 data of 35 years.

We thank you for pointing out this, and we have revised the overstated statement. To address the underlying concern that the conclusion requires stronger mechanistic support, we have substantially expanded both the observational basis and the mechanistic analysis to enhance robustness: (1) we conducted a comprehensive mechanistic analysis combining sensitivity analysis, environmental diagnostics, and trend decomposition to identify the contribution of key environmental factors, and (2) we performed idealized numerical model experiments to test the sensitivity of strong and weak tropical cyclones to dynamical disruption and

thermodynamic enhancement. Together, these analysis show that the intensity-dependent divergence in decay trends of strong and weak tropical cyclones are not only statistically robust but also physically consistent with distinct environmental sensitivity and rapid changing key environmental factors. We provide full details of each component below.

#2 The physical mechanism analysis should be strengthened by providing some necessary evidence aside from the results of machine learning. For example, the observed structural differences between strong and weak TCs, and the evidence on different responses of strong and weak TCs to dynamic and thermodynamic factors.

We thank you for this constructive comment and we have substantially strengthened the mechanistic analysis in the revised manuscript. We now provide direct observational and modeling evidence that strong and weak tropical cyclones respond divergently to environmental forcing: strong tropical cyclones are more sensitive to dynamic disruption while weak tropical cyclones respond primarily to thermodynamic factors. This intensity-dependent sensitivity, combined with observed environmental trends and numerical model experiments, provides a physically coherent explanation for the bifurcation in post-landfall decay. The revised manuscript substantiates this through three complementary lines of evidence:

First, we added a correlation analysis at both event-scale and year-scale for the expanded dataset (Table 1). Sensitivity analysis demonstrates distinct intensity-dependent sensitivity that strong tropical cyclones are significantly correlated to translation speed, soil moisture, and all dynamic factors (vertical wind shear, vorticity, vertical wind speed), while weak tropical cyclones respond primarily to thermodynamic factors (SST, ocean area ratio, 850 hPa humidity), with insignificant relationship to all dynamic variables.

Second, we combine these distinct sensitivities with observed trends in key environmental variables to explain the divergent decay trends, results demonstrate that the long-term trends in key environmental variables are physically coherent with the divergent decay timescale trends (Fig. 4). For strong tropical cyclones, drying soil moisture due to warming-enhanced evaporative demand (Qing et al., 2023; Seo et al., 2025), accelerating translation speeds associated with the enhanced steering flow under global warming (Hassanzadeh et al. 2020; Yamaguchi et al. 2020), and increasing atmospheric stability at lower latitudes linked to amplified upper-tropospheric warming and tropical expansion (Vecchi & Soden 2007; Kossin, 2014; Chen et al., 2022) all track the accelerating decay trend closely, particularly after 1999 (Fig. 4; Extended Data Fig. 8; Extended Data Fig. 9). For weak tropical cyclones, SST warming, increasing ocean area ratio, and enhanced atmospheric moisture co-vary closely with the prolonged persistence trend. A two-step trend decomposition further quantifies that these key environmental changes collectively contribute to 45.9%–49.9% of the observed

strong tropical cyclone decay trend and 63.7%–65.3% of the weak tropical cyclone trend, providing quantitative support for the proposed mechanisms (Fig 4. g, h).

Third, we conducted idealized landfall model simulations across four landfall intensity groups under varying VWS (0–15 m s^{-1}) and SST (300–303 K) environment (Fig. 5). The results confirm that VWS accelerates post-landfall decay across all intensity groups, but stronger tropical cyclones are more sensitive to the dynamical disruption. This differential sensitivity is mechanistically explained by structural diagnostics (Extended Data Fig. 11 and 12). SST warming and enhanced moisture can indeed increase the decay timescale for all storms, but weaker storms response more strongly to SST warming. Strong storms' large frictional energy demands render SST-induced thermodynamic benefits less important, while weak storms' modest dissipation rates allow them to effectively exploit the additional energy supply (Extended Data Fig. 13).

Together, these three lines of evidence provide a physically coherent and comprehensive mechanistic explanation for the observed intensity-dependent bifurcation.

Furthermore, we note that the key environmental factors identified in our analysis are projected to persist and potentially intensify under continued warming. We added a paragraph in the Discussion: "CMIP projections indicate that several key environmental drivers may persist: soil moisture faces continued decline (Sheffield and Wood, 2008; Yao et al. 2025); faster-moving landfalling tropical cyclone due to enhanced steering flow (Hassanzadeh, 2020); tropical higher atmospheric stability linked to amplified upper-tropospheric warming and Walker circulation weakening and tropical expansion (Vecchi & Soden 2007, Zhao et al 2026). The environment of strong landfalling storms potentially become increasingly unfavorable, while enhanced temperature and moisture will sustain weak storms persistence, suggesting the intensity-dependent bifurcation may keep or even amplify in the future."

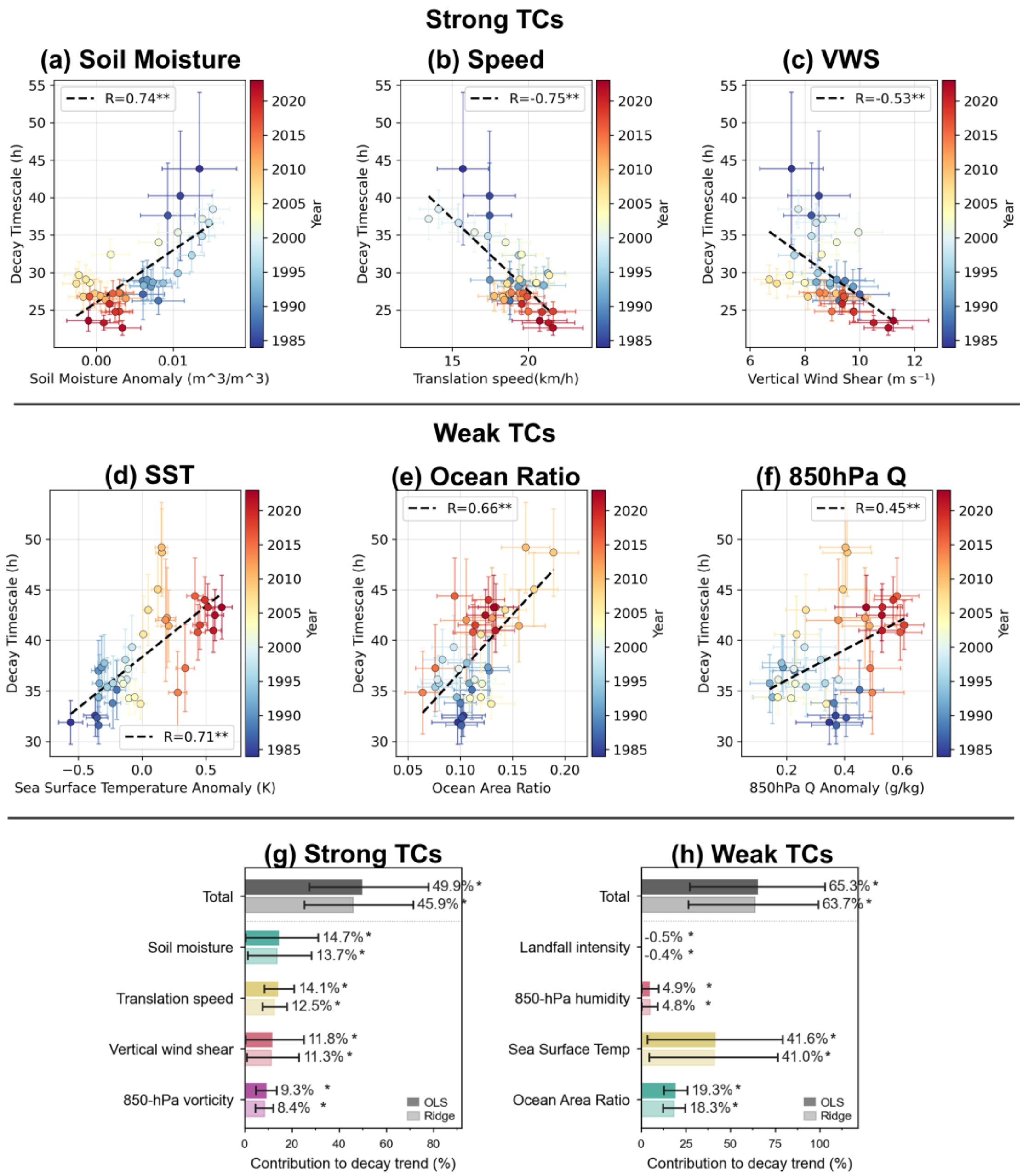

Figure 4. Relationships between key environmental factors and tropical cyclone decay timescale, and their contributions to the observed trends. *Scatter plots between annual-mean decay timescale and three most influential environmental factors for* ***(a–c)*** *strong and* ***(d–f)*** *weak tropical cyclones, with dot color indicating year and error bars denoting ±1 standard error in 5-year window. The dashed line shows the ordinary least-squares regression fit, with the Pearson correlation coefficient (R) and significance level indicated (* $P < 0.05$, ** $P < 0.01$). Estimated contributions of each environmental variable to the observed long-term trend in decay timescale for* ***(g)*** *strong and* ***(h)*** *weak tropical cyclones, respectively. Dark and light bars represent OLS and Ridge regression estimates, respectively, with error bars denoting 95% bootstrap confidence intervals. Asterisks denote significant contribution.*

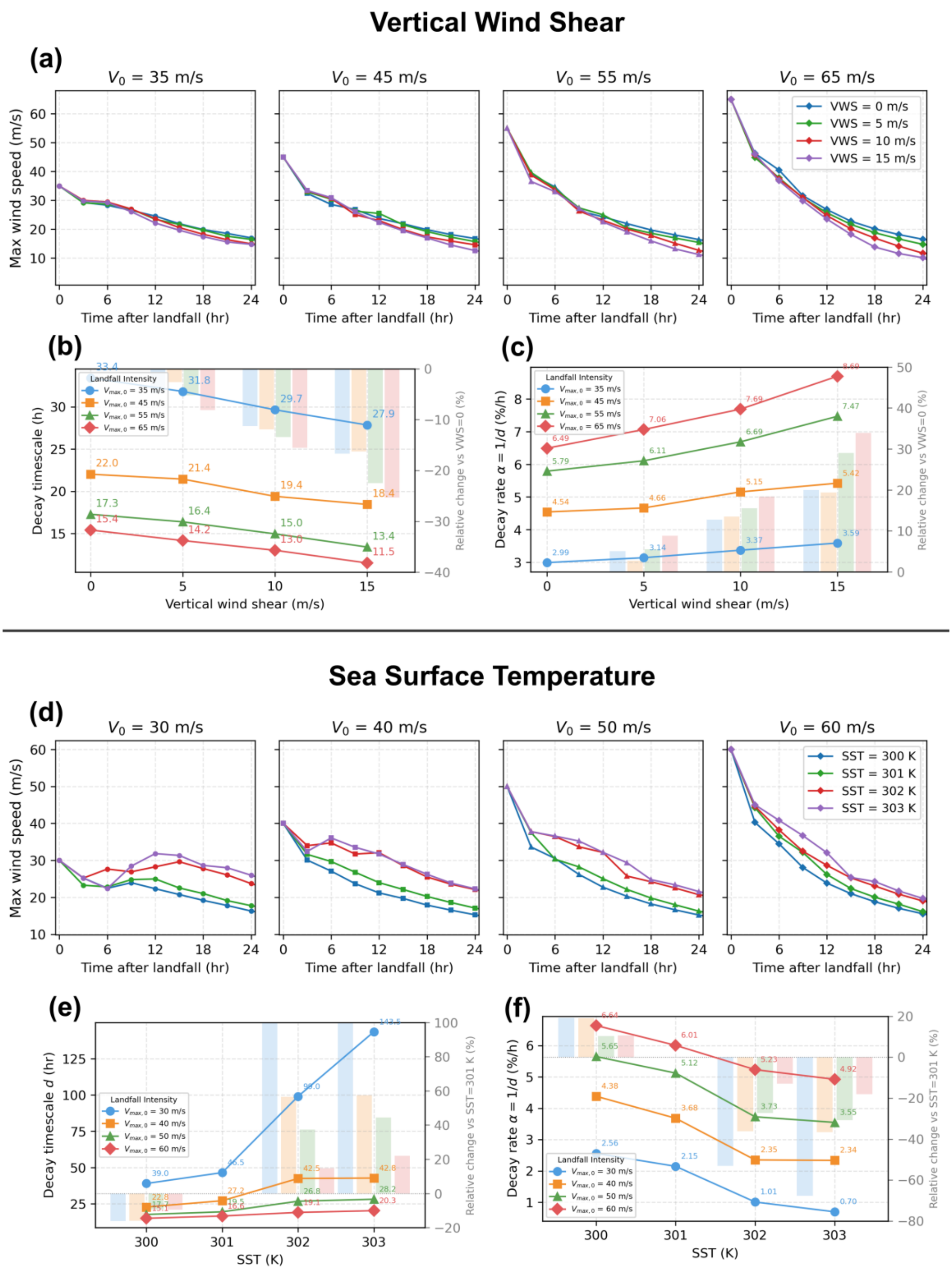

Figure 5. Post-landfall decay sensitivity to vertical wind shear and sea surface temperature in idealized CM1 simulations. (*a*) *Time series of maximum 10-m wind speed for tropical cyclones with landfall intensities of 35, 45, 55, and 65 m/s under four VWS environments (0, 5, 10, and 15 m/s), with SST fixed at 301 K.* ***(b)*** *Decay timescale d (left axis) and its relative change with respect to the VWS = 0 experiment (right axis, bars) as a function of VWS, for each landfall intensity group.* ***(c)*** *As in* ***(b),*** *but for the decay rate α = 1/d.* ***(d)*** *As in* ***(a)****, but for tropical cyclones with landfall intensities of 30, 40, 50, and 60 m/s under four*

SST conditions (300, 301, 302, and 303 K), with VWS set to zero. ***(e)*** *As in* ***(b)*** *but as a function of SST, with relative changes computed with respect to the SST = 301 K experiment.* ***(f)*** *As in* ***(e)****, but for the decay rate.*

Line 217-219: Why detrend the temperature? I am wondering the reasonability of drawing the limited predictive importance of temperature to TCs from the weak correlation after detrend. Since it is well known that the temperature, especially SST, has significant impact on TC activity, and is one of the most important predictors of TC activity (i.e., Elsner et al., 2008; Knutson et al., 2010; Li & Chakraborty, 2020).

We appreciate your questions. We are not disputing the well-established importance of SST for tropical cyclone activity. The issue we identified is specifically about the spurious temporal correlation between SST and strong tropical cyclone decay timescale.

Strong tropical cyclone decay timescale exhibits a significant negative correlation with SST (Event-scale $R = -0.13$, Year-scale $R = -0.61$, $P < 0.01$). This sign reversal is not a physical signal but rather a statistical artifact arising from the concurrent long-term trends in both variables: SST has warmed monotonically while strong tropical cyclone decay timescale has decreased, producing a spurious negative temporal correlation that does not reflect physical causation (Cummins et al., 2022).

To directly address whether SST physically affects strong tropical cyclone decay, we conducted idealized numerical model experiments as mentioned previously (Figure 5d, e, f). The results demonstrate that SST warming and enhanced humidity does slow post-landfall decay for all tropical cyclones, consistent with established physical understanding. However, the model outputs also reveal that weaker tropical cyclones are more sensitive to SST warming, as strong storms' larger frictional energy demands make the SST-induced thermodynamic benefit less important (Extended Data Fig. 13). In the real atmosphere, this SST-warming benefit for strong tropical cyclones is overwhelmed by other unfavorable factors.

We have revised the relevant section to make this interpretation explicit and have removed the detrending analysis that caused the confusion.

Line 227: Both strong and weak tropical cyclones have experienced decreased soil moisture.
We thank you for this important clarification. Both strong and weak tropical cyclones have experienced declining soil moisture at their landfall locations, especially an abrupt decline since 2000 and remains lower values in recent decades (Extended Data Fig. 8a; Extended Data Fig. 9a). The key distinction lies in the different sensitivity.

Strong tropical cyclone decay timescale shows significant correlations with soil moisture at both event-scale ($R = 0.19$, $P < 0.01$) and year-scale ($R = 0.74$, $P < 0.01$), while weak tropical cyclone decay timescale shows negligible correlation ($R = 0.01$) with soil moisture. This

might be because strong tropical cyclones possess shallower inflow boundary layers and more intense warm-core structures, enabling them to more efficiently convert surface enthalpy fluxes into intensity maintenance after landfall (Ren et al., 2020; Zhang et al., 2023), though this is also an avenue warrant future investigation. We have clarified this distinction in the revised manuscript.

Line 232: The statement of "regions experiencing moistening trends" needs support.

We have added statement with more evidence: "Additionally, 850 hPa specific humidity has increased significantly in the Northern Hemisphere basins (WP, EP, NA, NI; Extended Data Fig. 9d), its correlation with decay timescale confirms the role of enhanced atmospheric moisture in weak storm maintenance ($R = 0.45$, $P < 0.01$; Fig. 4f, Extended Data Fig. 8f)."

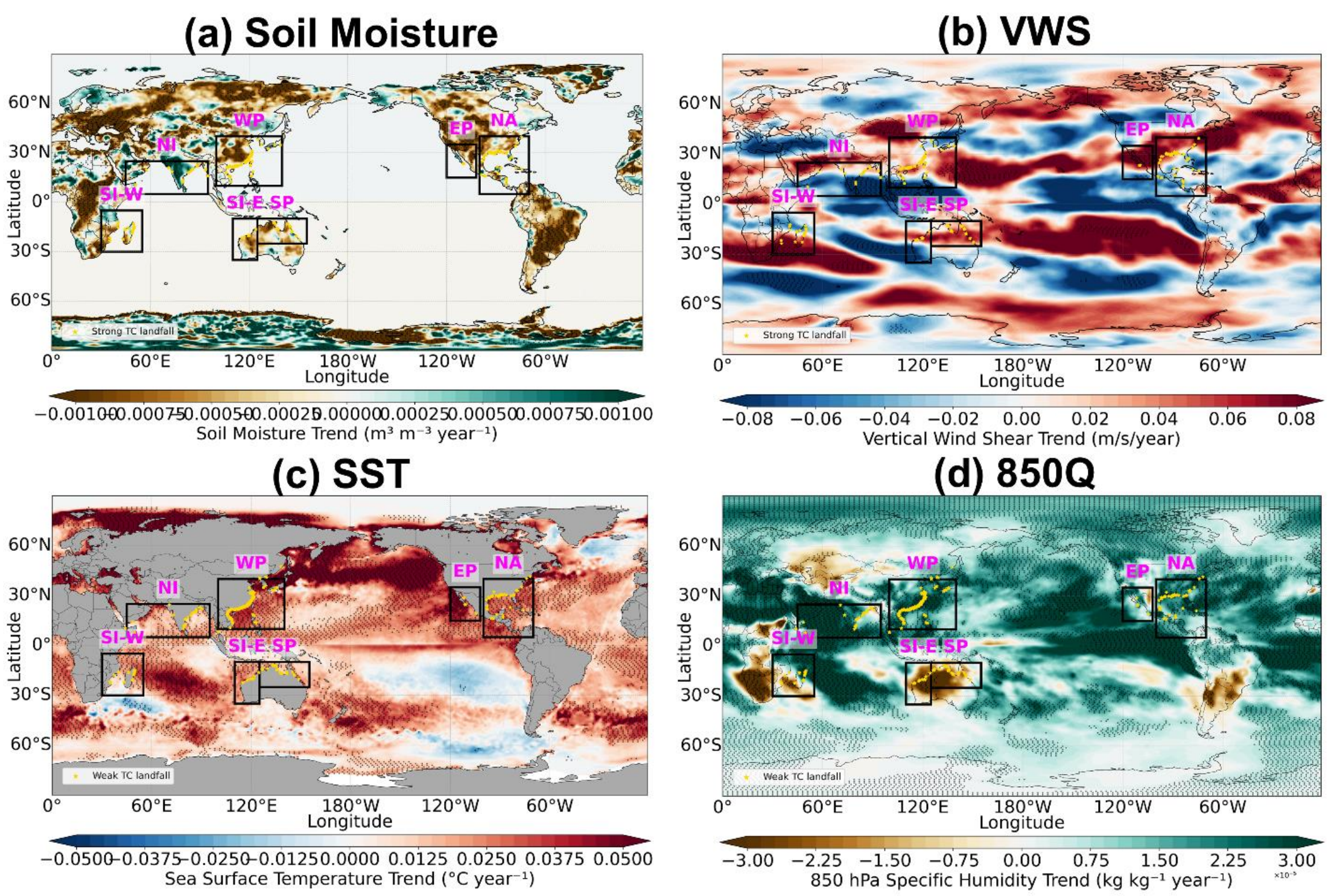

Extended Data Fig. 9. *Global spatial distribution of annual trends during 1999 to 2024 in key environmental variables* ***(a)*** *soil moisture,* ***(b)*** *vertical wind shear,* ***(c)*** *sea surface temperature, and* ***(d)*** *850-hPa specific humidity, computed over the Northern Hemisphere tropical cyclone season (May–October) and Southern Hemisphere tropical cyclone season (October–April). Stippling indicates grid points where the trend passed 95% statistically significant level ($P < 0.05$). Black rectangles delineate the six storm basin regions. Gold stars denote tropical cyclone landfall locations during 1999–2024 with strong tropical cyclone landfalls shown in* ***(a)*** *and* ***(b)****, and weak landfalls shown in* ***(c)*** *and* ***(d)****.*

Line 233-234: I don't understand why dryer soil would responsible to the more frequent

strong tropical cyclones in SP? If the "drying" means less precipitation, then why more frequent strong tropical cyclones cause less precipitation. Even though tropical cyclones are one important trigger of precipitation, it is not the only system that produces precipitation.
Thank you for raising this question. We clarify that "drying" in this context refers to a reduction in low-level atmospheric moisture. As shown in Extended Data Fig. 9d, the Southern Pacific (SP) and southern South Indian Ocean (SI) basins have experienced a declining trend in 850 hPa specific humidity, in marked contrast to the moistening trends observed in Northern Hemisphere. Such a drying trend results in faster decay for both strong and weak tropical cyclones in SP and SI (Extended data Fig. 2).

We have added a clearer statement: "Importantly, the Southern Hemisphere (SP, SI) shows drying trends (Extended Data Fig. 9d), consistent with the observed reduction in precipitation and atmospheric moisture (Timbal et al., 2006; Simpson et al., 2024). This regional heterogeneity is associated with faster decay of all storms in SP and SI (Extended Data Fig. 2)."

Figure 4: I would suggest replacing Figs 4b and 4c using Extended Data Fig 8, which provide more detailed information, or maybe consider to combine these figures.

We appreciate your suggestion. Fig. 4 has been replaced by new analysis.

Extended Data Table 3: Even though the authors claimed that the thermodynamic factors, particular the low-level moisture, are important drivers of slower decay of weak TCs, the correlation between these factors and weak TC decay timescale are not very high, and did not show obvious differences compared to that between strong TCs.

We thank you for your insightful comment. After expanding the dataset, for 850 hPa specific humidity, the year-scale correlation with decay timescale is 0.45 ($P < 0.01$) for weak tropical cyclones compared to 0.29 (insignificant) for strong tropical cyclones.

Crucially, our numerical model experiments provide independent process-level physical evidence. The SST sensitivity experiments demonstrate that weak tropical cyclones post-landfall decay respond far more strongly to SST warming and enhanced humidity than strong ones (Fig. 5d, e, f). This process-level physical evidence, combined with the sensitivity contrasts, provides robust support for the distinct thermodynamic sensitivity between strong and weak tropical cyclones.

References:

Elsner, J. B., Kossin, J. P., & Jagger, T. H. (2008). The increasing intensity of the strongest tropical cyclones. Nature, 455(7209), 92–95. https://doi.org/10.1038/nature07234

Knutson, T. R., McBride, J. L., Chan, J., Emanuel, K., Holland, G., Landsea, C., Held, I., Kossin, J. P., Srivastava, A. K., & Sugi, M. (2010). Tropical cyclones and climate change. Nature Geoscience, 3(3), 157–163. https://doi.org/10.1038/ngeo779
Li, L., & Chakraborty, P. (2020). Slower decay of landfalling hurricanes in a warming world. Nature, 587(7833), 230–234. https://doi.org/10.1038/s41586-020-2867-7

Others:

Line 45: ref #17 did not support the conclusion about decreasing cyclone durations from poleward shifts, please check.

Thank you for pointing out this, we have reorganized the paragraph: "However, emerging evidence reveals complexities: regional and temporal heterogeneity in post-landfall decay trends suggest that the thermodynamic framework alone cannot fully explain observed changes[17], while others report mixed intensity changes pre-landfall[18,19]. Beyond thermodynamic forcing, tropical cyclone intensity is highly sensitive to dynamic environmental conditions. Vertical wind shear (VWS) is widely recognized as an important dynamic inhibitor of tropical cyclone intensification, acting by tilting the vortex and ventilating the warm core[20-22]. Low-level vorticity and atmosphere stability further modulate TC convective organization and intensity evolution[23,24]."

Line 83: This conclusion should be made with cautious. In Lai et al. (2024) (also ref #10 in this study), the relationship between TC decay timescale and landfall intensity was also examined, which shows a significant ($p = 0.06$) positive correlation between them, i.e., stronger landfall intensity yields larger decay timescale (slower decay), which is the opposite of this study. One of the possible reasons causing the opposite results is that when the landfall intensity <25m/s, the decay timescale decreases sharply as landfall intensity increasing, while such a relationship does not exist for TCs with landfall intensity >25 m/s (Noted that analysis of Lai et al. (2024) was based on TCs with landfall intensity >24.5 m/s).

We have carefully compared our study with Lai et al. (2024). The difference arises from different analytical approaches rather than opposite results.

Lai et al. (2024) examined the year-scale correlation between annual mean decay timescale and annual mean landfall intensity for landfalling tropical cyclone in China only. In their Fig. 2f, the landfall intensity range is approximately 25–32 m s^{-1}, reflecting year-to-year variations in the mean intensity of landfalling storms. The positive correlation they report reflects a temporal co-trending signal that both landfall intensity and decay timescale experienced increasing trends, so the year-scale correlation is positive. Here we replicate their approach using only CMA data in Western Pacific for storms exceeding 24.5 m s^{-1} (2-min maximum

wind speed) from 1979 to 2024, we get similar results, showing positive relationship between decay timescale and landfall intensity (see Fig. A1 below).

In contrast, we mentioned that strong tropical cyclones decay faster than weak storms, which reflects the physical nature of tropical cyclone dynamic for individual storm at global scale. As shown in Fig. 2a where strong tropical cyclones lose intensity much more rapidly than weak ones after landfall. This is consistent with the foundational Kaplan–DeMaria exponential decay model (Kaplan and DeMaria, 1995), which has long established that major hurricane decay much faster than tropical storm. The two analyses therefore address different questions with physical meaning, rather than opposite results.

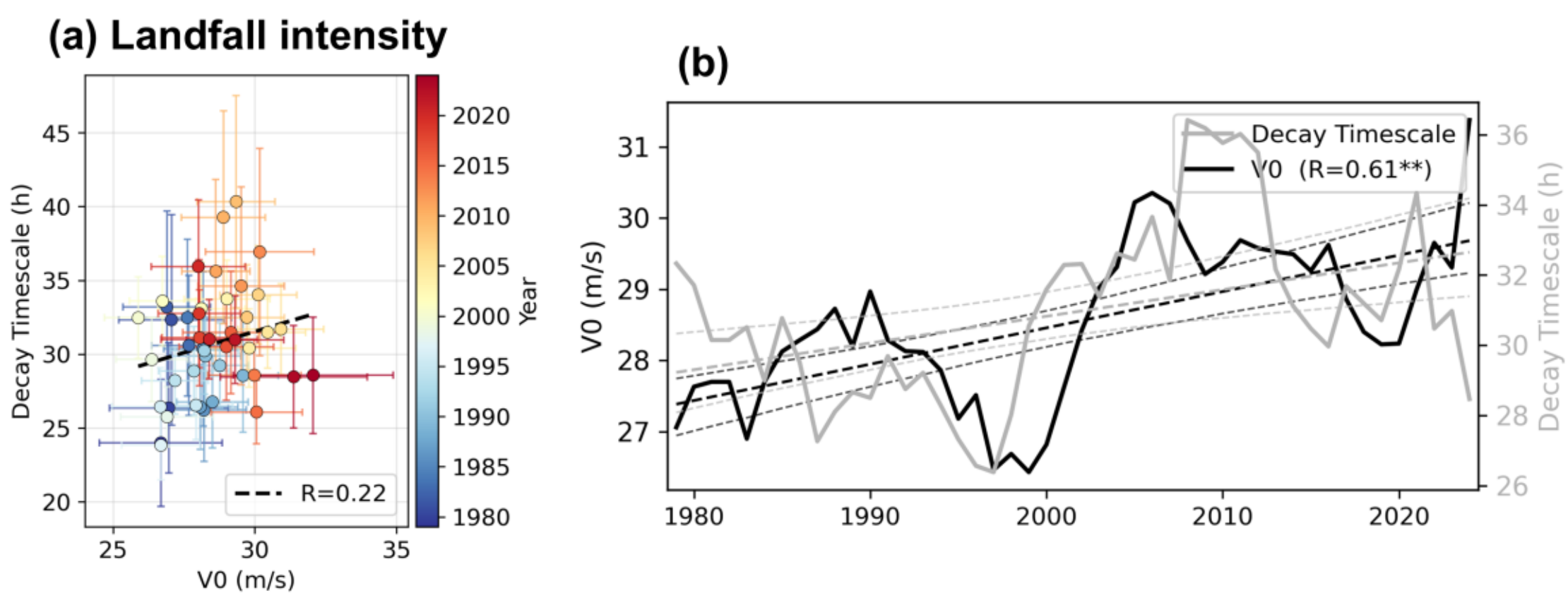

Fig. A1. ***(a)*** *Scatter plot between annual-mean decay timescale and landfall intensity for tropical cyclone with landfall intensity > 24.5 m/s, with point color indicating year and error bars denoting ±1 standard error of all events in 5-year window. The dashed line shows the OLS regression fit with the Pearson correlation coefficient indicated (** P < 0.01).* ***(b)*** *Five-year running mean time series of landfall intensity (black line) and decay timescale (gray line).*

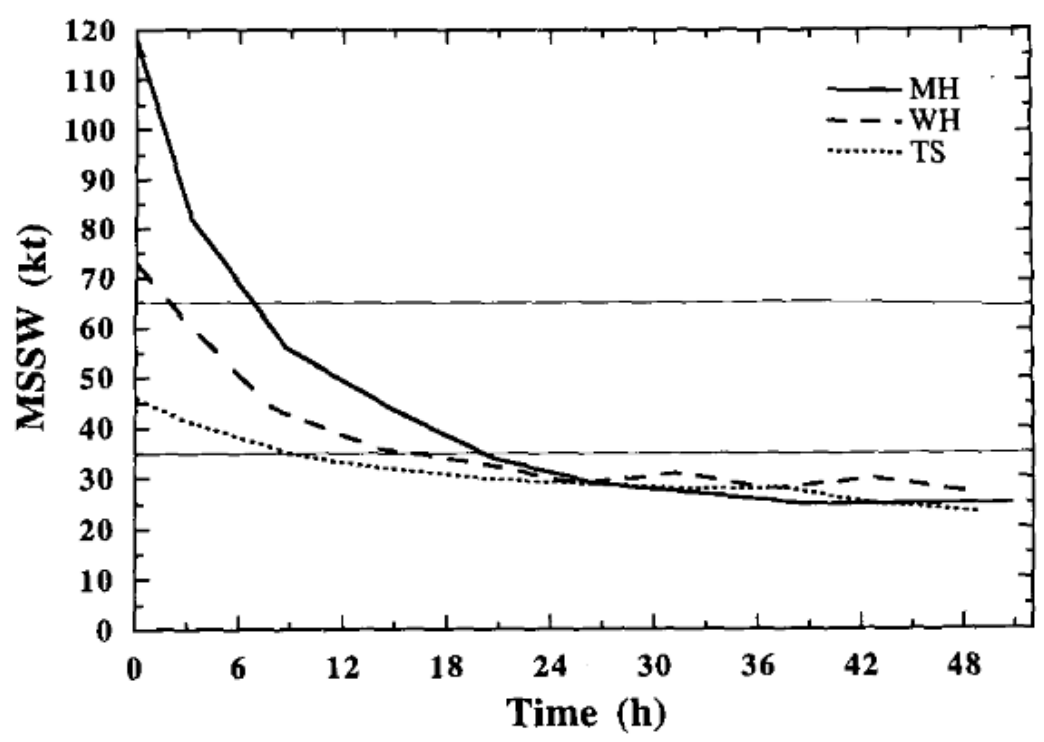

FIG. 2. The mean change in MSSW as a function of the elapsed time after landfall for the mean tropical storm (TS), weak hurricane (WH), and major hurricane (MH). The solid horizontal lines depict the threshold of hurricane- (65 kt) and tropical-storm- (35 kt) force MSSWs.

Fig. A2. *Derived from (Kaplan and DeMaria, 1995).*

Line 106-109: I have doubt on this statement, since the upper bound and lower bound are envelope bounds of all TCs, rather than the decay curve of any TC. For an extreme example (which may be not true), one strong storm (upper bound) can decay very fast and reach lower bound. May consider to show more detailed decay curves.

We thank you for pointing out this. We have separated strong and weak tropical cyclones and plotted their post-landfall wind speed time series independently in the revised Fig. 2a, showing the early and late period means with uncertainty bounds for each intensity category. Results show robust signals that strong tropical cyclones decay faster while weaker storms maintain higher wind speed for longer duration.

Here is the revised manuscript for your reference: "Post-landfall wind speed time series reveal the physical basis for intensity-dependent decay changes (Fig. 2a). Maximum sustained wind speeds follow exponential decay after landfall, with broadly similar mean patterns between periods. However, a divergence emerges: despite higher landfall intensities in the late period (2002–2024), strong tropical cyclones decay more rapidly, falling below early-period (1979–2001) wind speeds within approximately six hours after landfall. In contrast, weak tropical cyclones maintain higher wind speeds for longer durations in the late period."

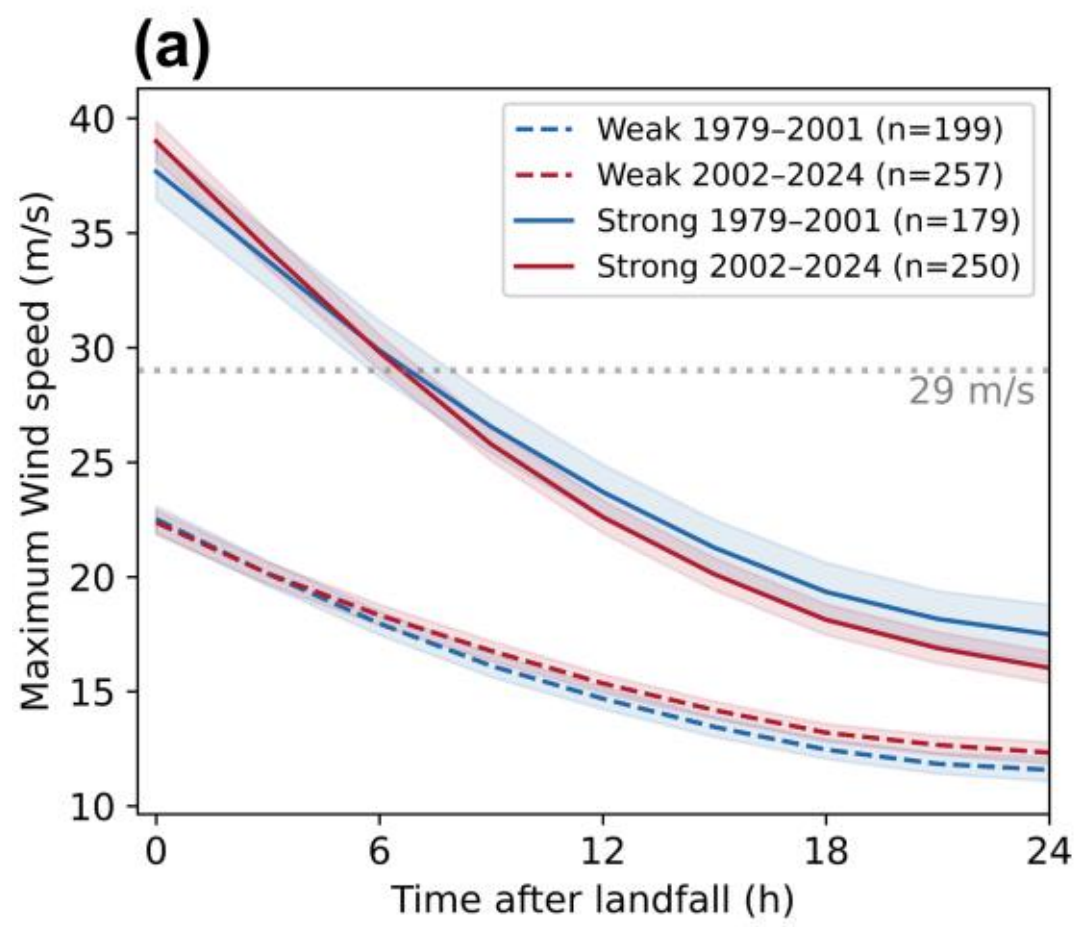

Fig. 2. ***(a)*** *Time series of post-landfall wind speed for strong (solid line) and weak (dashed line) tropical cyclones, showing early (blue, 1979–2001) versus late (red, 2002–2024) period means with variability (shaded).*

Fig 3 and Extended Data Fig. 7: The statistic significant level of differences between periods were provided, however, what are the differences refer to? differences of rainfall/PDI between periods, or the decay rate of rainfall/PDI between periods?

The asterisks in Fig. 3 and Extended Data Fig. 7 denote statistically significant differences in

the mean rainfall and PDI values, respectively, between the early (1979–2001) and late (2002–2024) periods within each coastal distance bin. We have clarified this in the revised figure captions.

Fig 3: I would suggest to revise these maps: The 3D rotation is not necessary, which will not provide any more information, but makes it harder to identify the geographical location. Further, it is difficult to distinguish the colors (and the indicated values) in rotated views. It is hard to tell, at what distance, how much the difference in precipitation rate is.

We appreciate your suggestion. Revised as suggested.

Reference

Harper, B. A., Kepert, J. D. & Ginger, J. D. Guidelines for Converting Between Various Wind Averaging Periods in Tropical Cyclone Conditions. WMO/TD-No. 1555 (World Meteorological Organization, 2010)

Li, L., Chakraborty, P. Slower decay of landfalling hurricanes in a warming world. Nature 587, 230–234 (2020).

Lai, Y., Gu, X., Wei, L. et al. Slower-decaying tropical cyclones produce heavier precipitation over China. npj Clim Atmos Sci 7, 99 (2024).

Chan. K., Chan. J., Zhang. K., Wu. Y. Uncertainties in tropical cyclone landfall decay. NPJ Clim. Atmos. Sci. 5(1), 1–8 (2022).

Vecchi, G. A., & Soden, B. J. Increased tropical Atlantic wind shear in model projections of global warming. *Geophysical Research Letters*, 34(8), L08702 (2007).

Seo, K.-W., et al. Abrupt sea level rise and Earth's gradual pole shift reveal permanent hydrological regime changes in the 21st century. *Science* **387**, 1408–1413 (2025).

Qing, Y., Wang, S., Yang, ZL. *et al.* Accelerated soil drying linked to increasing evaporative demand in wet regions. *npj Clim Atmos Sci* **6**, 205 (2023).

Hassanzadeh, P., Lee, CY., Nabizadeh, E. *et al.* Effects of climate change on the movement of future landfalling Texas tropical cyclones. *Nat Commun* **11**, 3319 (2020).

Yamaguchi. M. et al. Global warming changes tropical cyclone translation speed. Nat. Commun. 11, 47 (2020).

Kossin. J., Emanuel. K., Vecchi. G. The poleward migration of the location of tropical cyclone maximum intensity. Nature 509(7500), 349–352 (2014).

Chen, J. et al. Future thermodynamic impacts of global warming on landfalling typhoons and their induced storm surges to the Pearl River Delta region as inferred from high-resolution regional models. J. Clim. 35, 4905–4926 (2022).

Sheffield, J., Wood, E.F. Projected changes in drought occurrence under future global warming from multi-model, multi-scenario, IPCC AR4 simulations. Clim Dyn 31, 79–105 (2008).

Yao, L., Leng, G., Yu, L. *et al.* Emergent constraints on global soil moisture projections under climate change. *Commun Earth Environ* **6**, 39 (2025).

Zhao, K., Zhao, H., Klotzbach, P.J. *et al.* Anthropogenic warming projected to drive a decline in global tropical cyclone frequency in CMIP6 simulations. *npj Clim Atmos Sci* **9**, 58 (2026).
Cummins, D. P., Stephenson, D. B., & Stott, P. A. Could detection and attribution of climate change trends be spurious regression? *Climate Dynamics*, 58, 2333–2354 (2022).
Ren, Y., Zhang, J. A., Vigh, J. L., Zhu, P., Liu, H., Wang, X., & Wadler, J. B. An Observational Study of the Symmetric Boundary Layer Structure and Tropical Cyclone Intensity. *Atmosphere*, *11*(2), 158 (2020).
Timbal. B., Arblaster. J., Power. S. Attribution of the Late-Twentieth-Century Rainfall Decline in Southwest Australia. *J. Climate* 19(10), 2046–2062 (2006).
Simpson, I. R., et al. Observed humidity trends in dry regions contradict climate models. *Proc. Natl Acad. Sci. U.S.A.* **121**, e2302480120 (2024).
Kaplan. J., DeMaria. M. A Simple Empirical Model for Predicting the Decay of Tropical Cyclone Winds after Landfall. J. Appl. Meteorol. 34(11), 2499–2512 (1995).

Response to Reviewer 2

Reviewer #2 (Remarks to the Author):

This study explores the post-landfall decay of strong and weak TCs. It is interesting to find that the strong storms now weaken 22% faster after landfall and weak storms persist 19% longer inland in recent 35 years. Then analyzed the environmental influencing factors using machine learning methods. The article has innovative findings, but lacks appropriate in-depth analysis. Overall, this study holds important practical significance. However, there are some major issues that need to be addressed.

1.In your study, you choose decay timescale as the indicator to analyze the post-landfall decay of strong and weak TCs. You mentioned that “larger decay timescale indicates slower decay”. Why don't you just use the intensity decay rate (intensity change after landing divided by the duration after landing) of landfalling TCs to analyze? Are the results of intensity decay rate similar to the decay timescale?

We appreciate your insightful comments. Decay timescale (τ) was chosen as the primary metric because it normalizes for the strong dependence of absolute intensity loss on landfall intensity, as a storm making landfall at 60 m s^{-1} will lose wind speed far more rapidly in absolute terms than one making landfall at 25 m s^{-1}, making direct comparison across intensity categories physically misleading. The decay timescale, defined as the time for intensity to reach 1/e of its landfall value, is widely used in the tropical cyclone decay literature.

Here we have conducted a robustness analysis in Extended Data Fig. 4. As expected, the direct decay rate (wind speed loss per hour within 24 hours of landfall) scales nearly linearly with landfall intensity, such that the absolute magnitude of changes is much smaller for weak tropical cyclones. However, the conclusions are entirely consistent with the decay timescale results: “Analysis of the direct decay rate (wind speed loss within 24 hours of landfall) yields consistent results (Extended Data Fig. 4). Weak tropical cyclones show a decreasing decay rate trend (−0.03 m/s/yr, 95% CI: −0.04 to −0.02, $R = 0.57$, $P < 0.01$), while strong tropical cyclones show an increasing trend (0.09 m/s/yr, 95% CI: 0.06–0.12, $R = 0.68$ $P < 0.01$), with corresponding period differences (weak: 10.7 vs. 10.0 m/s; strong: 20.9 vs. 23.4 m/s; threshold = 28.6 m s^{-1}) corroborating the decay timescale results.”

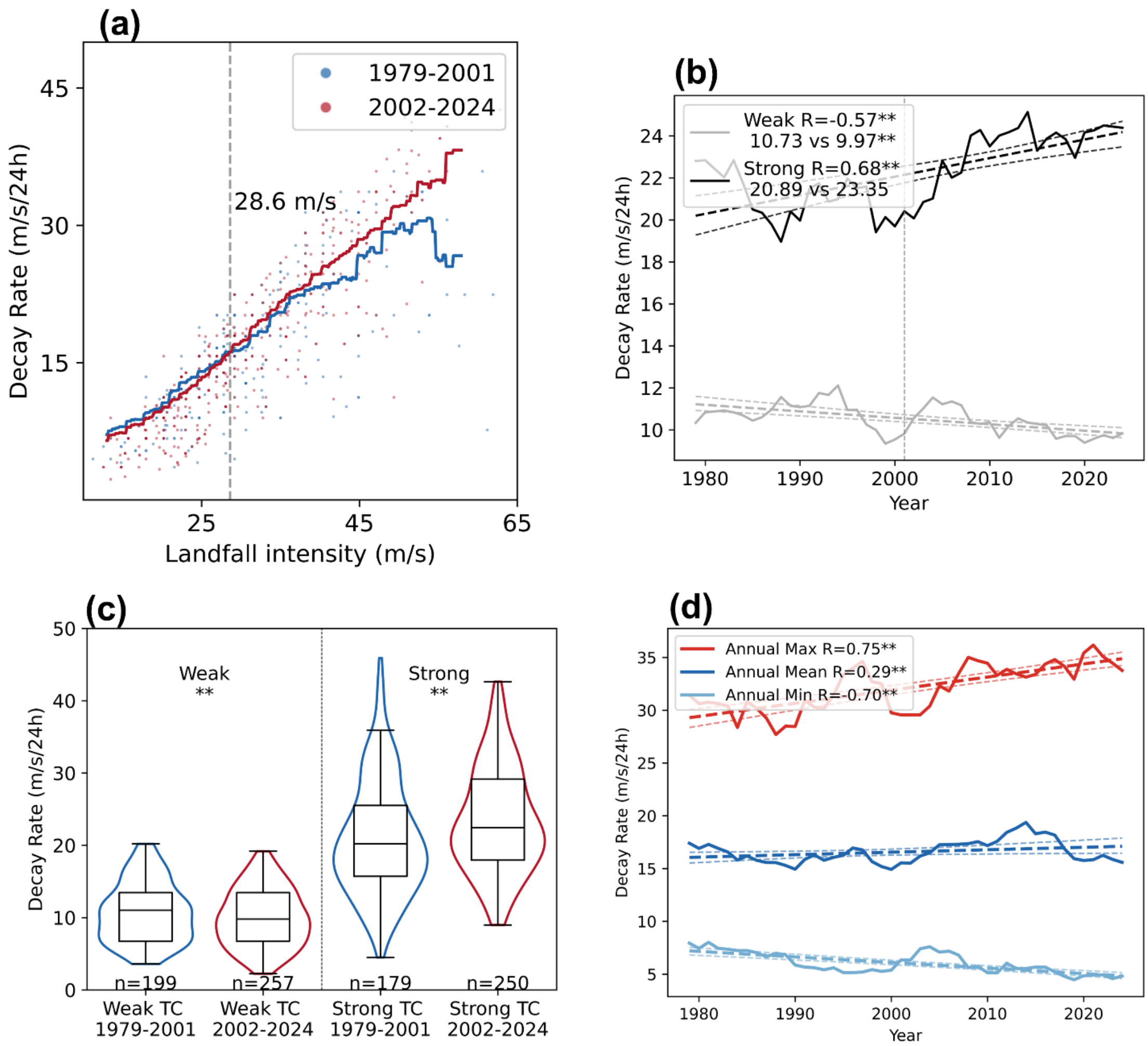

Extended Data Fig. 4. ***(a)*** *Relationship between landfall intensity and direct decay rate (wind speed loss within 24 hours of landfall) for global landfall tropical cyclone. Blue and red lines show vertical average of the scatter plot during early and late period; dashed vertical line marks the threshold of intensity where decay timescale changes conversely.* ***(b)*** *Temporal trends in decay rate for strong and weak tropical cyclones using five-year running means.* ***(c)*** *Distribution of decay rate across intensity categories and periods. Violin plots combine probability density with box plots; sample sizes indicated as "n". Asterisks denote significant differences between periods (**P < 0.01).* ***(d)*** *Annual trend of decay rate for global tropical cyclones, with red, blue, and light blue lines representing annual maximum, annual mean, and annual minimum. Trend coefficients, 95% CI, and significance levels are shown in* ***b*** *and* ***d****.*

2.Please clarify the locations of strong and weak TCs in Figure 2a

We thank you for pointing out this. We have separated strong and weak tropical cyclones and plotted their post-landfall wind speed time series independently in the revised Fig. 2a,

showing the early and late period means with uncertainty bounds for each intensity category. Results show robust signals that strong tropical cyclones decay faster while weaker storms maintain higher wind speed for longer duration.

Here is the revised manuscript for your reference: "Post-landfall wind speed time series reveal the physical basis for intensity-dependent decay changes (Fig. 2a). Maximum sustained wind speeds follow exponential decay after landfall, with broadly similar mean patterns between periods. However, a divergence emerges: despite higher landfall intensities in the late period (2002–2024), strong tropical cyclones decay more rapidly, falling below early-period (1979–2001) wind speeds within approximately six hours after landfall. In contrast, weak tropical cyclones maintain higher wind speeds for longer durations in the late period."

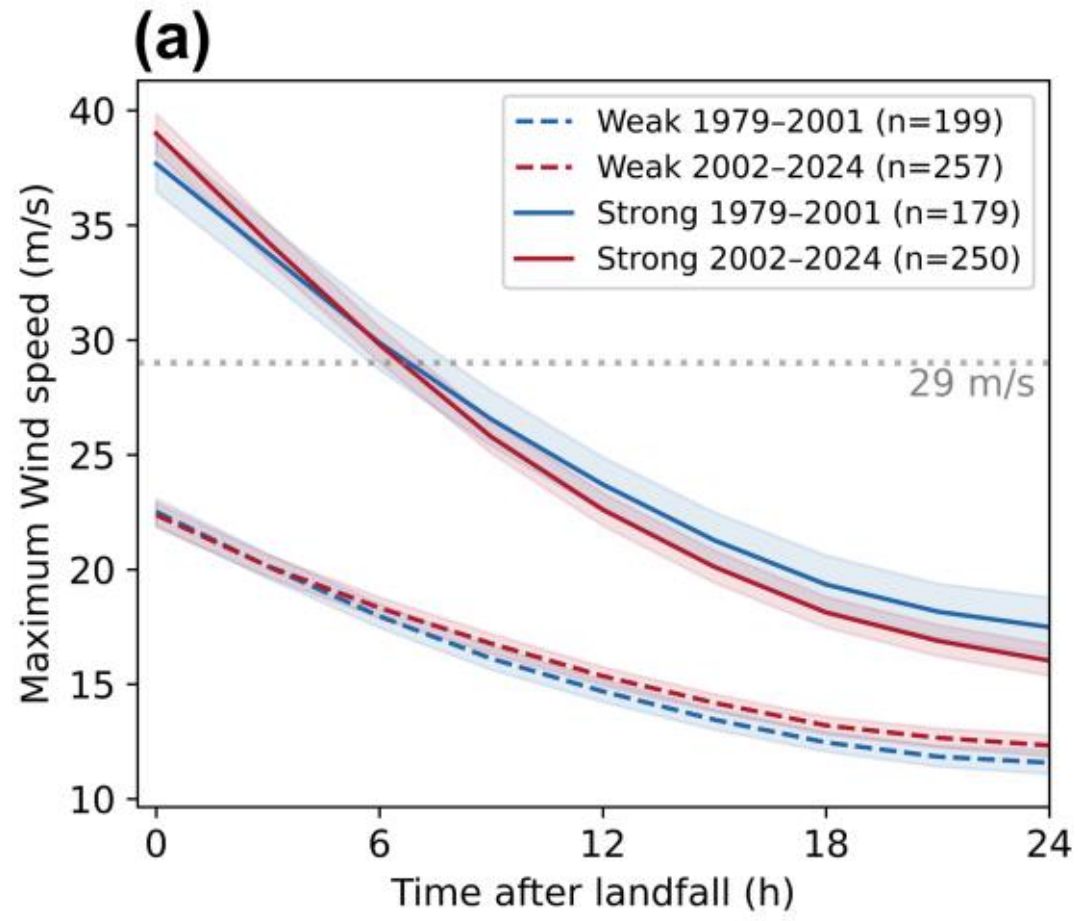

Fig. 2. ***(a)*** *Time series of post-landfall wind speed for strong (solid line) and weak (dashed line) tropical cyclones, showing early (blue, 1979–2001) versus late (red, 2002–2024) period means with variability (shaded).*

3.In line 118-120: You mentioned that "Independent validations using time-to-decay to tropical depression (11 m s⁻¹) confirms these opposing trends (Extended Data Fig. 2), indicating robust intensity-dependent changes in tropical cyclone decay rate." the meaning of "time-to-decay" that I understand is the duration of post-landfall TCs. However, time-to-decay cannot accurately represent the intensity decay rate of ladnfalling TCs. Because different landfall intensity can cause different decay rates after landfall although the time-to-decay is same. Therefore, I don't think Extended Data Fig. 2 can fully support your above conclusion. Why not use the intensity decay rate directly?

Thank you again for your suggestion. We agree that time-to-decay to tropical depression intensity may not fully isolate the decay rate from the effects of differing landfall intensities. We have replaced this analysis with the direct intensity decay rate (wind speed loss within 24 hours of landfall) as the robustness metric (Extended Data Fig. 4), please see our response

above.

4.Suggest adding some mechanism analysis of environmental factors to analyze the reasons for the different intensity decay of strong and weak typhoons. The entire article only has statistical analysis and lacks appropriate mechanism analysis.

We sincerely thank you for your suggestion and we have substantially strengthened the mechanistic analysis in the revised manuscript. We now provide direct observational and modeling evidence that strong and weak tropical cyclones respond divergently to environmental forcing: strong tropical cyclones are more sensitive to dynamic disruption while weak tropical cyclones respond primarily to thermodynamic factors. This intensity-dependent sensitivity, combined with observed environmental trends in recent decades and numerical model experiments, provides a physically coherent explanation for the bifurcation in post-landfall decay. The revised manuscript substantiates this through three complementary lines of evidence:

First, we added a correlation analysis at both event-scale and year-scale for the expanded dataset (Table 1). Sensitivity analysis demonstrates distinct intensity-dependent sensitivity that strong tropical cyclones are significantly correlated to translation speed, soil moisture, and all dynamic factors (vertical wind shear, vorticity, vertical wind speed), while weak tropical cyclones respond primarily to thermodynamic factors (SST, ocean area ratio, 850 hPa humidity), with insignificant relationship to all dynamic variables.

Second, we combine these distinct sensitivities with observed trends in key environmental variables to explain the divergent decay trends, results demonstrate that the long-term trends in key environmental variables are physically coherent with the divergent decay timescale trends (Fig. 4). For strong tropical cyclones, drying soil moisture due to warming-enhanced evaporative demand (Qing et al., 2023; Seo et al., 2025), accelerating translation speeds associated with the enhanced steering flow under global warming (Hassanzadeh et al. 2020; Yamaguchi et al. 2020), and increasing atmospheric stability at lower latitudes linked to amplified upper-tropospheric warming and tropical expansion (Vecchi & Soden 2007; Kossin, 2014; Chen et al., 2022) all track the accelerating decay trend closely, particularly after 1999 (Fig. 4; Extended Data Fig. 8; Extended Data Fig. 9). For weak tropical cyclones, SST warming, increasing ocean area ratio, and enhanced atmospheric moisture co-vary closely with the prolonged persistence trend. A two-step trend decomposition further quantifies that these key environmental changes collectively contribute to 45.9%–49.9% of the observed strong tropical cyclone decay trend and 63.7%–65.3% of the weak tropical cyclone trend, providing quantitative support for the proposed mechanisms (Fig 4. g, h).

Third, we conducted idealized landfall model simulations across four landfall intensity groups

under varying VWS (0–15 m s^{-1}) and SST (300–303 K) environment (Fig. 5). The results confirm that VWS accelerates post-landfall decay across all intensity groups, but stronger tropical cyclones are more sensitive to the dynamical disruption. This differential sensitivity is mechanistically explained by structural diagnostics (Extended Data Fig. 11 and 12). SST warming and enhanced moisture can indeed decrease the decay rate for all storms, but weaker storms response more strongly to SST warming. Strong storms' large frictional energy demands render SST-induced thermodynamic benefits less important, while weak storms' modest dissipation rates allow them to effectively exploit the additional energy supply (Extended Data Fig. 13).

Together, these three lines of evidence provide a physically coherent and comprehensive mechanistic explanation for the observed intensity-dependent bifurcation.

5.Many studies (e.g. Wu et al. 2005, Tu et al. 2009, Park et al. 2011, Liu et al. 2020; Zhang et al. 2023) have found that the landfall latitude of TCs in the Western Pacific (WP) tend to move northward as a whole, but your statistical results show that the landfall latitude tend to move southward (Extended Data Fig. 4f). Can you explain why your results are contrary to previous work?

Zhang, L., Cheng, T. F., Lu, M., Xiong, R., & Gan, J. (2023). Tropical cyclone stalling shifts northward and brings increasing flood risks to East Asian Coast. Geophysical Research Letters, 50(10), e2022GL102509.

Wu, L., B. Wang, and S. Geng, 2005: Growing typhoon influence on East Asia. Geophys. Res. Lett., 32, L18703, https://doi.org/10.1029/2005GL022937.

Tu, J. Y., C. Chou, and P.-S. Chu, 2009: The abrupt shift of typhoon activity in the vicinity of Taiwan and its association with western North Pacific–East Asian climate change. J. Climate, 22, 3617–3628, https://doi.org/10.1175/2009JCLI2411.1.

Park,D.-S. R., C.-H.Ho, J.-H. Kim, and H.-S. Kim, 2011: Strong landfall typhoons in Korea and Japan in a recent decade. J. Geophys. Res., 116, D07105, https://doi.org/10.1029/2010JD014801.

Liu, L., Wang, Y., Zhan, R., Xu, J., & Duan, Y. (2020). Increasing destructive potential of landfalling tropical cyclones over China. Journal of Climate, 33: 3731-3743.

We thank you for raising this important point. We have carefully compared relevant literature and considered more recent papers. We clarify that our results are not contrary to the previous studies, but reflect different time periods and intensity stratification.

Many references focus on an early period before 2010. Extending our analysis to 1979–2024 with a multi-agency global dataset reveals that weak tropical cyclones indeed exhibit a continuous and statistically significant poleward shift ($R = 0.62$, $P < 0.01$), consistent with prior literature. Strong tropical cyclones, however, show a rapid poleward shift through

approximately 2005, followed by an equatorward reversal in recent decades, yielding an overall increasing trend (R = 0.28, P = 0.06). This non-monotonic behavior is consistent with the documented recent reversal in the poleward shift of Western Pacific tropical cyclones (Sun et al. 2018). Furthermore, a recent study demonstrates that the observed poleward migration is largely driven by a tripolar Pacific SST variability pattern rather than a monotonic forced trend, and that equatorward migration is simulated when this pattern reverses (Zhou et al. 2025).

Notably, when all tropical cyclones in the WP are considered, the landfall latitude shows a robust poleward shift (R = 0.51, P < 0.01; Fig. A3), broadly consistent with the poleward migration documented in the cited literature. However, a clear equatorward reversal emerges after approximately 2005, consistent with the recently documented reversal in WNP tropical cyclone poleward migration driven by multi-decadal Pacific SST variability.

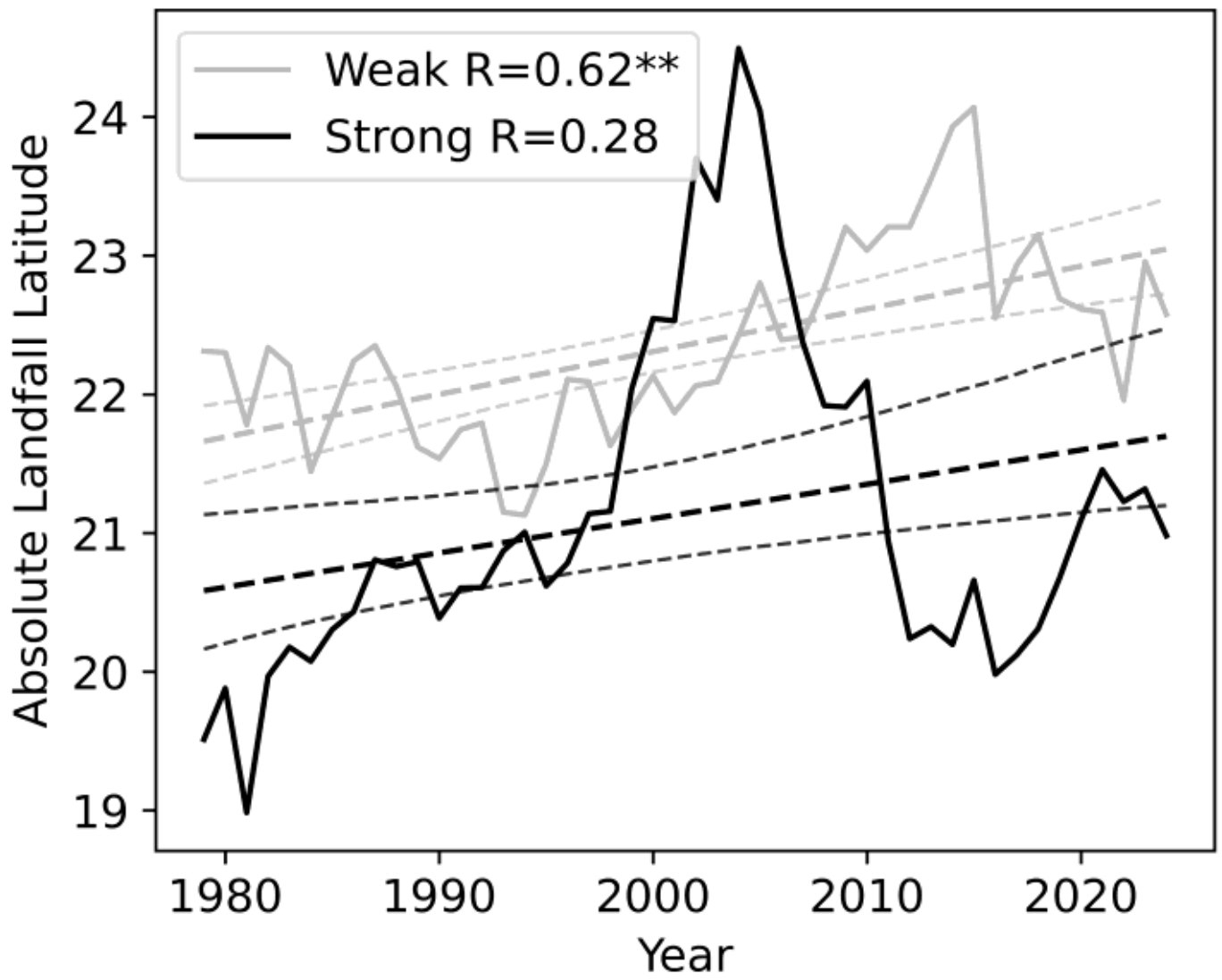

Fig. 2. ***(d)*** *Temporal trends in mean absolute landfall latitude, for global strong and weak tropical cyclones using five-year running means.*

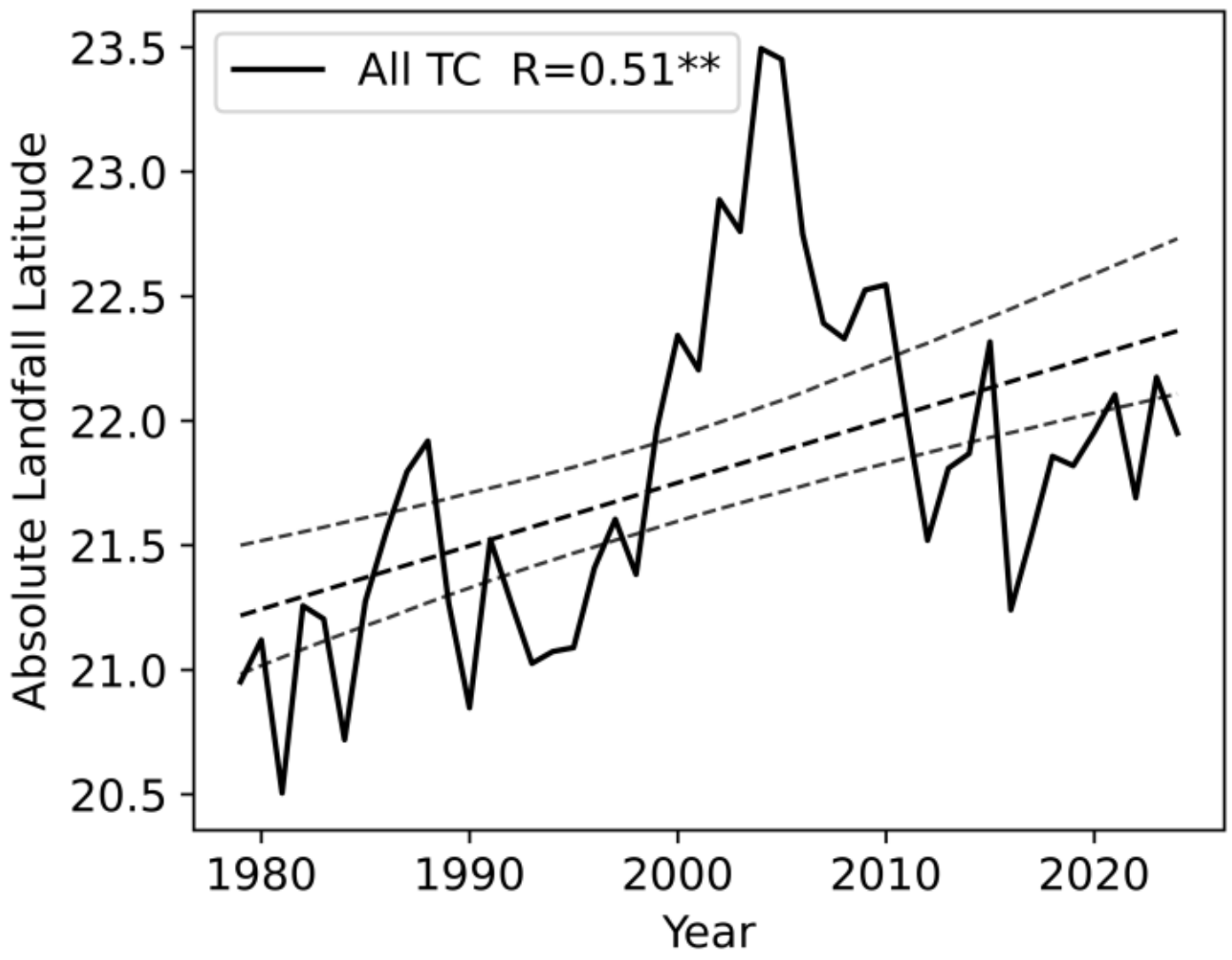

Fig. A3. *Temporal trends in mean absolute landfall latitude for all landfall tropical cyclones in WP.*

6.Please explain the meaning of "ocean area ratio" and how to calculated it. It is averaged from landfall to TD? It is related to TC size? When does a TC make landfall start counting? Is the calculation starting from the center of the typhoon crossing the coastline? It will affect the ocean area ratio.

Ocean area ratio (OAR) was introduced following Chan et al. (2022) and represents the proportion of ocean grid points within 150 km of the tropical cyclone center, averaged at 6, 12, and 18 hours post-landfall, to characterize the degree of ocean surface exposure during the critical early decay window (see Method section Environmental factors).

Specifically, landfall is defined as the moment the tropical cyclone center crosses the coastline from ocean to land, identified using 3-hourly IBTrACS position data and the MODIS land cover dataset at 500 m resolution. At each of the three post-landfall time steps (6, 12, and 18 hours), we identify all MODIS grid points within 150 km of the tropical cyclone center and compute the fraction classified as ocean. The three values are then averaged to yield a single OAR for each storm event. A higher OAR indicates that a greater proportion of the storm's inner-core region remains over ocean during the early post-landfall period, allowing continued energy replenishment.

We chose a 150 km radius rather than the 200 km used in Chan et al. (2022) because 150 km better represents the typical inner-core radius of tropical cyclones. Sensitivity tests using a 200 km radius yield consistent results.

7.In line 126-130: please explain the relationship between decay rate of weak and strong TCs

and the landfall latitude. Why strong TC equatorward shirt is favorable to intensity decay? The lines of 131-136 cannot explain the different decay of weak and strong TCs incent decades. Suggest analyzing the underlying surface, environmental field, and characteristics of the typhoon itself to identify the reasons for this phenomenon.

Thank you for your insightful comment. The accelerated decay of strong tropical cyclones in recent decades reflects a convergence of multiple unfavorable environmental changes at landfall locations, such as faster translation speed, dryer soil moisture, and higher atmosphere stability, as now documented in the revised manuscript through climate trend analysis (Fig. 4, Extended Data Fig. 8) and numerical model experiments (Fig. 5). Importantly, vertical wind shear has intensified across most basins in subtropical regions during recent decades (Extended Data Fig. 9b), consistent with anthropogenic increases in atmospheric stability at lower latitudes linked to amplified upper-tropospheric warming and tropical expansion (Vecchi & Soden 2007; Kossin 2014; Chen et al. 2022). The equatorward shift of strong tropical cyclone landfall locations in recent decades reinforces this dynamic unfavorability, especially in coastal southern China and the southern United States (see gold stars in Extended Data Fig. 9b below).

Our numerical model sensitivity experiments further demonstrate that strong tropical cyclones are more sensitive to VWS increases than weak storms (Fig. 5 a, b, c), and that enhanced atmospheric stability substantially accelerates post-landfall decay for strong storms.

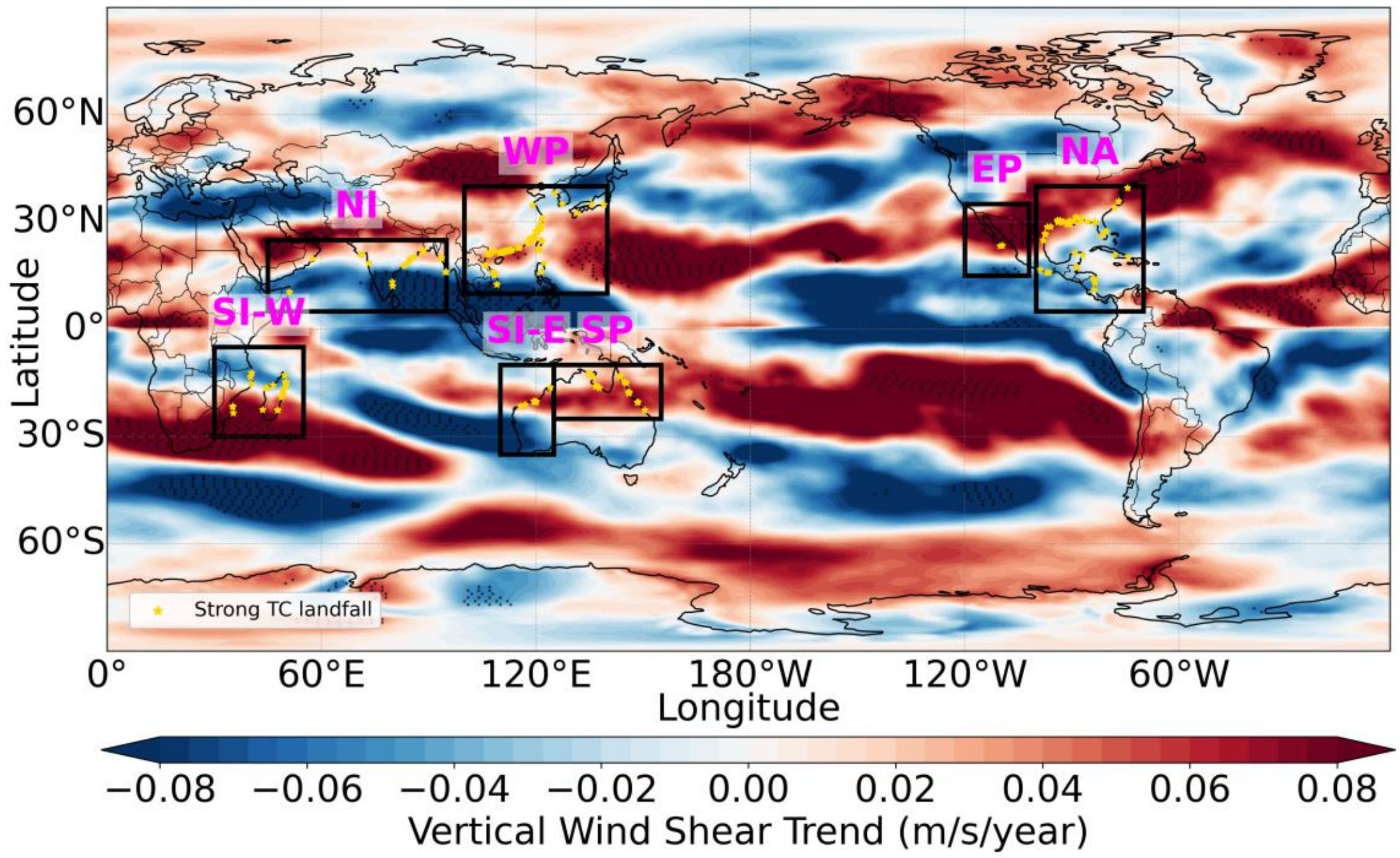

Extended Data Fig. 9 (b) *Global spatial distribution of linear trends during 1999 to 2024 in vertical wind shear, computed over the Northern Hemisphere tropical cyclone season (May–October) and Southern Hemisphere tropical cyclone season (October–April). Stippling*

indicates grid points where the trend passed 95% statistically significant level (P < 0.05). Black rectangles delineate the six storm basin regions. Gold stars denote strong tropical cyclone landfall locations during 1999–2024.

8.The distribution of strong and weak typhoons in Extended Data Fig. 7 does not correspond?

Thank you for this observation. The y-axes differ between panels (a) and (b) because the PDI and PDI decay rate of strong tropical cyclones are approximately five times magnitude larger than those of weak tropical cyclones; using a unified axis would compress the weak TC panel to the point where period differences become indiscernible. As the primary purpose of this figure is to compare period differences within each intensity category, separate y-axes were adopted to maximize clarity.

9.Minor

Figure 3 (in line 183-184): (a,b) change to (c,d)

Revised as suggested.

Reference

Chan. K., Chan. J., Zhang. K., Wu. Y. Uncertainties in tropical cyclone landfall decay. NPJ Clim. *Atmos. Sci*. 5(1), 1–8 (2022).

Sun, Y. *et al.* A recent reversal in the poleward shift of western North Pacific tropical cyclones. *Geophys. Res. Lett.* **45**, 9944–9952 (2018).

Zhou, W., Leung, L.R., Chang, CC. *et al.* Poleward migration of tropical cyclones over 1980–2024 is dominated by Pacific variability. *Nat. Geosci.* **19**, 42–51 (2026).

Lu, J., Vecchi, G. A., & Reichler, T. Expansion of the Hadley cell under global warming (vol 34, art no L06805, 2007). *Geophysical Research Letters*, 34(14), 14808 (2007).

Vecchi, G. A., & Soden, B. J. Increased tropical Atlantic wind shear in model projections of global warming. *Geophysical Research Letters*, 34(8), L08702 (2007).

Kossin. J., Emanuel. K., Vecchi. G. The poleward migration of the location of tropical cyclone maximum intensity. Nature 509(7500), 349–352 (2014).

Chen, J. et al. Future thermodynamic impacts of global warming on landfalling typhoons and their induced storm surges to the Pearl River Delta region as inferred from high-resolution regional models. J. Clim. 35, 4905–4926 (2022).

Response to Reviewer 3

Reviewer #3 (Remarks to the Author):

This manuscript investigates the opposing trends in post-landfall decay of strong and weak tropical cyclones. Analysing 506 events from 1990–2024, the authors show that strong storms exhibit a smaller decay timescale, while weak storms show a longer decay timescale. The study highlights the non-uniform response of post-landfall decay under climate change. The topic is relevant to tropical cyclone post-landfall risk, and the manuscript is generally well structured. I have few questions regarding the physical explanation behind the reported trends. Please see my comments below.

Minor comments:

Discussion (Line 260-269):

The opposing trends are related to structural differences between strong and weak storms. However, it is not clear why strong storms show a decreasing trend in decay timescale, while weak storms show an increasing trend (Fig. 2c). The authors suggest that contrasting climate regimes for weak and strong storms are responsible for these trends, yet TC characteristics still appear to be the dominant drivers (Fig. 4). Could this be related to strong storms becoming stronger while weak storms become weaker under climate change?

We thank you for this insightful question. We first note that Fig. 1b demonstrates that even at the same landfall intensity, decay timescales differ substantially between the early (1979–2001) and late periods (2002–2024) for both strong and weak tropical cyclones, indicating that environmental factors beyond landfall intensity play an important role.

Furthermore, we have substantially strengthened the mechanistic analysis in the revised manuscript through three complementary approaches.

First, we added a correlation analysis at both event-scale and year-scale for the expanded dataset (Table 1). Sensitivity analysis demonstrates distinct intensity-dependent sensitivity that strong tropical cyclones are significantly correlated to translation speed, soil moisture, and all dynamic factors (vertical wind shear, vorticity, vertical wind speed), while weak tropical cyclones respond primarily to thermodynamic factors (SST, ocean area ratio, 850 hPa humidity), with insignificant relationship to all dynamic variables.

Second, we combine these distinct sensitivities with observed trends in key environmental variables to explain the divergent decay trends, results demonstrate that the long-term trends in key environmental variables are physically coherent with the divergent decay timescale trends (Fig. 4). For strong tropical cyclones, drying soil moisture due to warming-enhanced evaporative demand (Qing et al., 2023; Seo et al., 2025), accelerating translation speeds

associated with the enhanced steering flow under global warming (Hassanzadeh et al. 2020; Yamaguchi et al. 2020), and increasing atmospheric stability at lower latitudes linked to amplified upper-tropospheric warming and tropical expansion (Vecchi & Soden 2007; Kossin, 2014; Chen et al., 2022) all track the accelerating decay trend closely, particularly after 1999 (Fig. 4; Extended Data Fig. 8; Extended Data Fig. 9). For weak tropical cyclones, SST warming, increasing ocean area ratio, and enhanced atmospheric moisture co-vary closely with the prolonged persistence trend. A two-step trend decomposition further quantifies that these key environmental changes collectively contribute to 45.9%–49.9% of the observed strong tropical cyclone decay trend and 63.7%–65.3% of the weak tropical cyclone trend, providing quantitative support for the proposed mechanisms (Fig 4. g, h).

Third, we conducted idealized landfall model simulations across four landfall intensity groups under varying VWS (0–15 m s^{-1}) and SST (300–303 K) environment (Fig. 5). The results confirm that VWS accelerates post-landfall decay across all intensity groups, but stronger tropical cyclones are more sensitive to the dynamical disruption. This differential sensitivity is mechanistically explained by structural diagnostics (Extended Data Fig. 11 and 12). SST warming and enhanced moisture can indeed decrease the decay rate for all storms, but weaker storms response more strongly to SST warming. Strong storms' large frictional energy demands render SST-induced thermodynamic benefits less important, while weak storms' modest dissipation rates allow them to effectively exploit the additional energy supply (Extended Data Fig. 13).

Together, these three lines of evidence provide a physically coherent and comprehensive mechanistic explanation for the observed intensity-dependent bifurcation.

Introduction:

While the authors review studies emphasizing the role of thermodynamics (e.g., SST and moisture) in tropical cyclone behaviour, it may be helpful to also include some context of the dynamical controls on tropical cyclones, especially since the manuscript compares TC sensitivities to thermodynamic and dynamical factors.

We sincerely thank you for your suggestions. We have included more context about the dynamical controls in the introduction:

"Beyond thermodynamic forcing, tropical cyclone intensity is highly sensitive to dynamic environmental conditions. Vertical wind shear (VWS) is widely recognized as an important dynamic inhibitor of tropical cyclone intensification, acting by tilting the vortex and ventilating the warm core[20-22]. Low-level vorticity and atmosphere stability further modulate TC convective organization and intensity evolution[23,24].

Tropical cyclone post-landfall decay is also highly associated with a suite of dynamic and

surface factors including translation speed, soil moisture, atmospheric vorticity, and atmospheric stability[25-29]. Critically, many of these factors have undergone changes in recent decades: soil moisture has declined rapidly after 2000 driven by warming-enhanced evaporative demand[30,31]; observation and model reveal an intensified VWS at lower latitudes linked to tropical expansion and Walker circulation weakening[32,33] and is projected to increase in the future[34]. These findings suggest that simple thermodynamics explanations overlook physical interactions with key environmental factors."

Line 79-80: Should "decay rate" refer to "decay timescale"? It may be helpful to use consistent terminology throughout the manuscript.

Thank you for pointing out this, we have revised accordingly.

Line 141: The purpose of Fig. 2a is not entirely clear to me.

We thank you for pointing out this. The revised Fig. 2a presents the post-landfall maximum wind speed time series separately for strong and weak tropical cyclones, comparing the early and late period. This figure provides a direct wind speed-level visualization of the intensity-dependent bifurcation: "Post-landfall wind speed time series reveal the physical basis for intensity-dependent decay changes (Fig. 2a). Maximum sustained wind speeds follow exponential decay after landfall, with broadly similar mean patterns between periods. However, a divergence emerges: despite higher landfall intensities in the late period (2002–2024), strong tropical cyclones decay more rapidly, falling below early-period (1979–2001) wind speeds within approximately six hours after landfall. In contrast, weak tropical cyclones maintain higher wind speeds for longer durations in the late period."

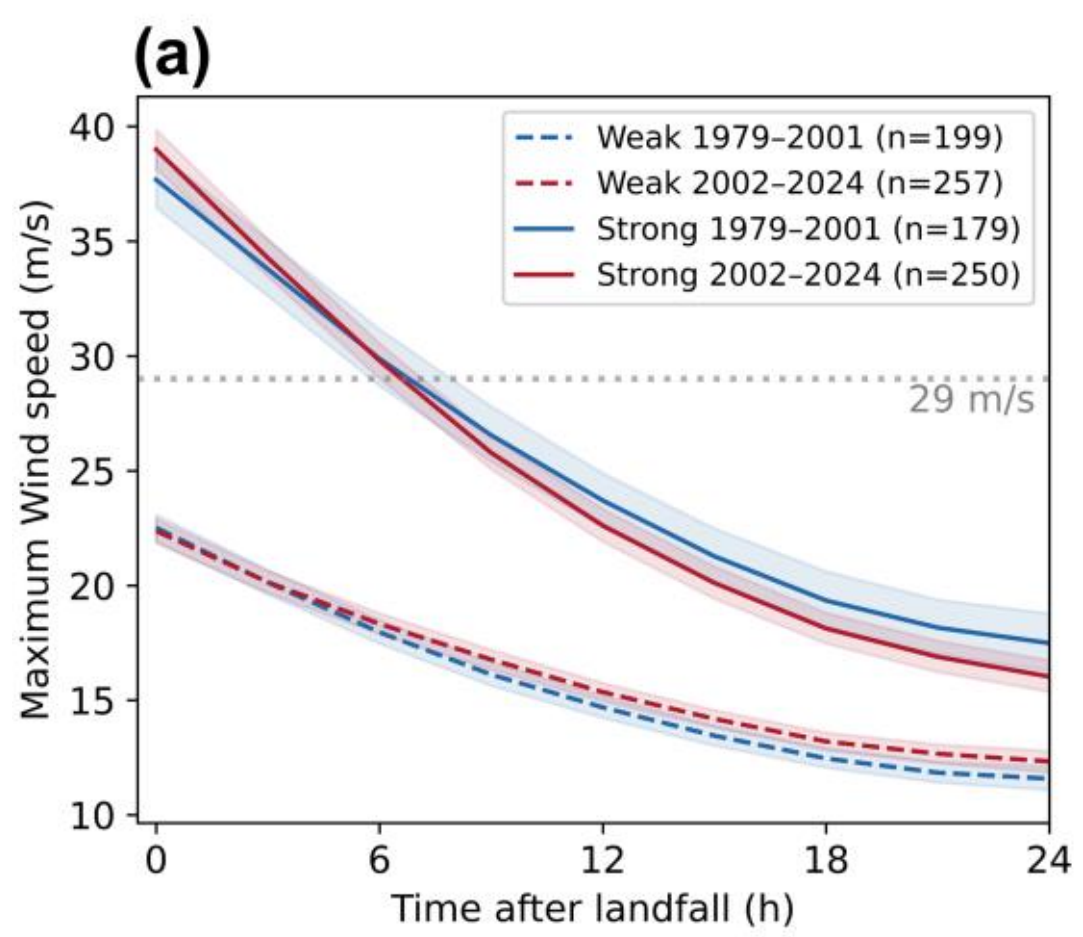

Fig. 2. ***(a)*** *Time series of post-landfall wind speed for strong (solid line) and weak (dashed line) tropical cyclones, showing early (blue, 1979–2001) versus late (red, 2002–2024) period*

means with variability (shaded).

Line 183: (a,b)->(c,d)

Revised as suggested.

We thank you for your constructive feedback, which has substantially strengthened our work. We have carefully addressed your suggestions and concerns through additional analyses and discussions. Below, we respond point-by-point to your comments (shown in blue), with our responses in black and additions to the manuscript highlighted in red.

Response to Reviewer 1

I have carefully read the authors' point-by-point responses and examined the revised manuscript. I am pleased with the thorough and thoughtful revisions. The authors have addressed all my concerns: they have substantially expanded the observational dataset (from 506 to 885 landfalling events), extended the analysis period to 1979-2024, added multi-institution data, and performed sensitivity tests on thresholds and time splits. And most importantly, it provided robust mechanistic support through correlation analyses, environmental trend decomposition, and idealized numerical model experiments (CM1). The revised manuscript has been greatly improved in statistically robustness and physically coherence. I would like to support the publication of this study in Nature Communications after revising the following suggestions:

Minor comments:

1. In the revised Fig. 2a, the time series for strong and weak TCs are now shown separately. Please ensure that the caption clearly defines what the solid/dashed lines and blue/red shadings represent (e.g., standard error or standard deviation).

Thank you for this suggestion. We have revised the figure caption of Fig. 2 to explicitly define all visual elements. The updated caption now specifies that solid and dashed lines represent post-landfall wind speed for strong and weak tropical cyclones, respectively. The shading represents the 95% bootstrap confidence interval (n = 10,000 resamples). We have applied the same clarification to Extended Data Fig. 6. The revised caption reads:

Fig. 2. Intensity-dependent changes in tropical cyclone post-landfall decay from 1979 – 2024. ***(a)*** *Time series of post-landfall wind speed for strong (solid line) and weak (dashed line) tropical cyclones, showing early (blue, 1979–2001) versus late (red, 2002–2024) period means; shading denotes the 95% bootstrap confidence interval (CI, n=10,000 resamples). Gray dashed line indicate the 29 m s⁻¹ threshold between strong and weak storms.* ***(b)*** *Distribution of decay timescales across intensity categories and periods. Violin plots combine probability density with box plots; sample sizes indicated as "n". Asterisks denote significant differences between periods (**P < 0.01). Temporal trends in* ***(c)*** *mean decay timescale and* ***(d)*** *mean absolute landfall latitude indicating poleward migration patterns, for strong and weak tropical cyclones using seven-year running means. Trend coefficients, 95% bootstrap CI, and significance levels are shown in* ***c*** *and* ***d****.*

2. In the manuscript, both "Ocean Area Ratio" and "ocean area ratio" were used. Please maintain consistency in terminology and check other similar places. Besides, please incorporate "OAR" and the citation (i.e., Chan et al., 2022) into the Section Materials and Methods – Environmental factors, making it easy for readers to find the OAR.

Thank you for pointing this out. We have standardized the terminology throughout the manuscript, adopting "Ocean Area Ratio (OAR)" at first mention and using the abbreviation

“OAR” consistently thereafter. We have also revised the Materials and Methods section (Environmental factors) to explicitly incorporate the abbreviation and the citation:

OAR represents the proportion of ocean grid points within 150 km of storm centers based on MODIS land cover data, averaged at 6-, 12-, and 18-hours post-landfall[17] to characterize surface exposure during the decay window.

We have carefully checked the entire manuscript to ensure consistent use of other terminology.

3. I appreciate that you have removed the potentially misleading detrending analysis and now clearly state that SST warming slows the decay for all storms, but its benefit is overwhelmed by other unfavorable factors for strong TCs. Please ensure that no ambiguous wording about the role of SST remains in the final text.

We appreciate your careful check. We have reviewed the entire manuscript and added a sentence to the Discussion to ensure the SST role is clear:

Accelerating translation speeds and increasing atmospheric stability that are directly linked to dynamic disruption, together with a trend toward drier soils, physically associated with the accelerated decay of global strong tropical cyclones in recent decades. Although SST warming also acts to slow the decay of strong tropical cyclones, this thermodynamic benefit is overwhelmed by the unfavorable dynamic and land-surface forcings. In contrast, SST warming and enhanced atmospheric moisture are strongly associated with weak cyclones’ prolonged inland persistence.

The existing statement in the main text also clearly notes that “SST warming does in fact slow decay for strong tropical cyclones,” and this is now consistently reinforced throughout the manuscript.

4. For CM1 experiments, they use fixed SST and no background flow, which simplifies reality. I would appreciate a sentence acknowledging that these experiments isolate specific mechanisms, but that real-world interactions (e.g., with soil moisture gradients or coastal geometry) may further modulate the response.

Thank you for your insightful suggestion. We have added a new sentence to the limitations paragraph in the Discussion section to explicitly acknowledge the idealized nature of the CM1 experiments:

The idealized CM1 simulations, while valuable for isolating the sensitivity of post-landfall decay to VWS and SST, employ fixed SST, no background environmental flow, and simplified surface conditions; real-world interactions such as soil moisture gradients, coastal geometry, and land surface heterogeneity may further modulate the intensity-dependent responses.

5. It is suggested to carefully reviewing all maps shown in the figures, particularly national borders.

Thank you very for this reminder. We have carefully reviewed all maps presented in the figures to ensure that national borders are displayed in accordance with standard cartographic conventions and journal guidelines. National boundaries in related figures are rendered using the Natural Earth dataset (50 m resolution) as implemented in the Cartopy mapping library.

Response to Reviewer 2

The revised paper is an improvement and can be accepted with some revisions.

1. You divide TCs into strong TCs and weak TCs. And you show that strong storms weaken faster after landfall, whereas weak storms persist 14.5% longer inland. Is this related to the annual changes in TC intensity? Has the strong TC been strengthening year by year and the weak TC been weakening year by year?

Thank you for raising this important point. We have conducted additional analysis to test whether trends in storm intensity could explain the observed divergent decay trends, including co-trend analysis, and partial correlation analysis. Results show that changes in landfall intensity are not the primary driver of the observed decay trends.

The late period (2002–2024) does contain a higher proportion of strong tropical cyclones compared to early period (1979–2001, Fig. A1), consistent with global warming-driven intensification of storm intensity. However, the event-scale correlation between landfall intensity and decay timescale is only $R = 0.06$ ($P > 0.05$) for strong tropical cyclones. While weak tropical cyclones show a higher event-scale correlation ($R = 0.24$, $P < 0.01$), the shift in their landfall intensity distribution is minimal.

At the year scale (new Extended Data Fig. 11), correlations between landfall intensity and decay timescale are insignificant for both strong ($R = -0.12$) and weak storms ($R = -0.13$, both $P > 0.05$), and their temporal evolutions are decoupled: strong-storm intensity rose most during 1984–2000 while decay timescale showed no consistent trend, then declined sharply post-2000 while landfall intensity change modestly; weak-storm intensity showed a negligible long-term trend even as decay timescale rose sharply during 2000–2012, then enhanced slightly after 2015 while mean intensity fell rapidly.

Partial correlation controlling for landfall intensity confirms this: the decay timescale trend remains highly significant and nearly unchanged for both strong (partial $R = -0.68$ vs. raw $R = -0.70$) and weak storms (partial $R = +0.77$ vs. raw $R = +0.72$, all $P < 0.01$), indicating intensity accounts for little of the trend.

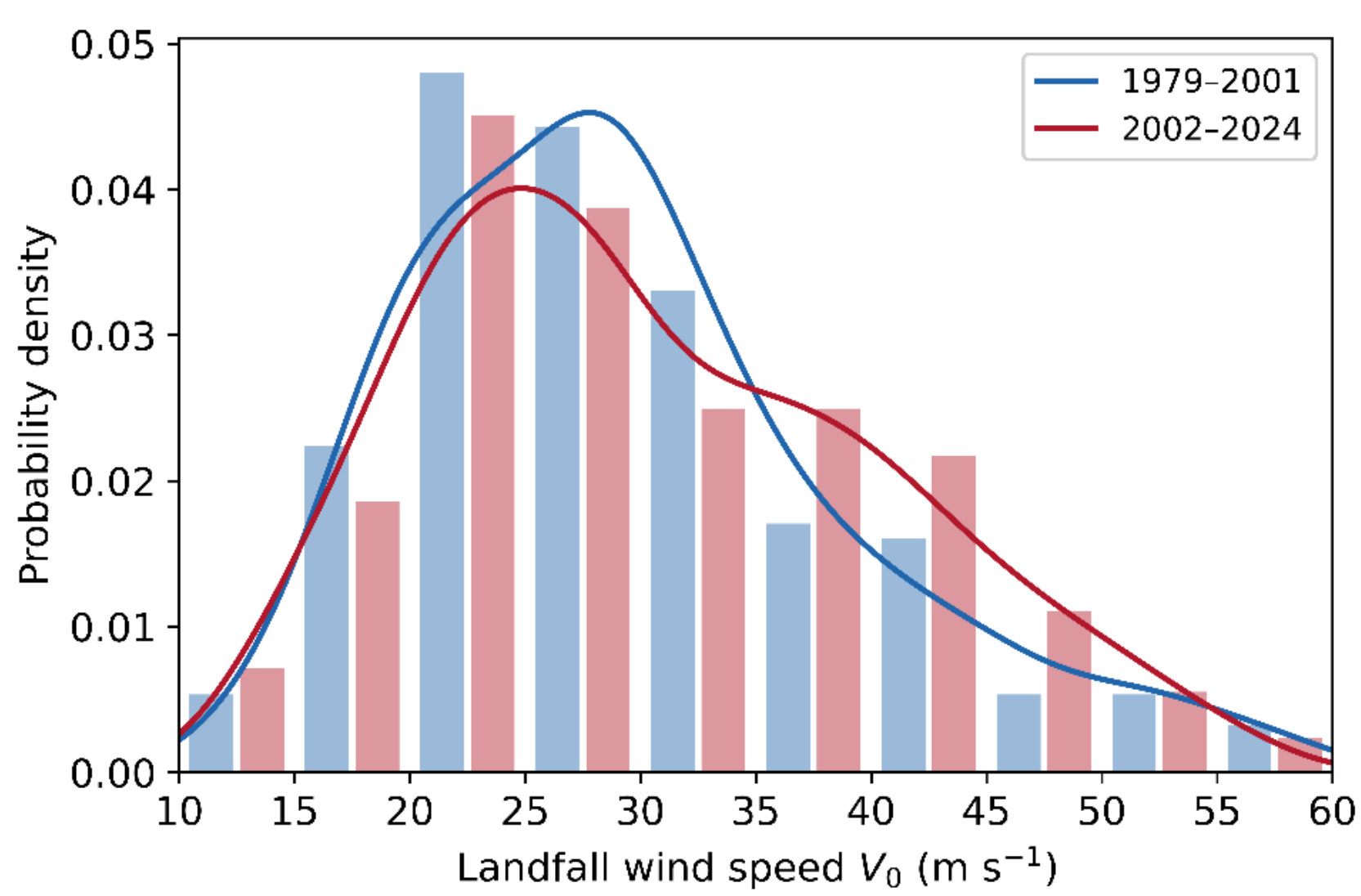

Fig. A1. *Probability density distributions (PDF) of landfall intensity for the early (blue, 1979–2001) and late (red, 2002–2024) periods.*

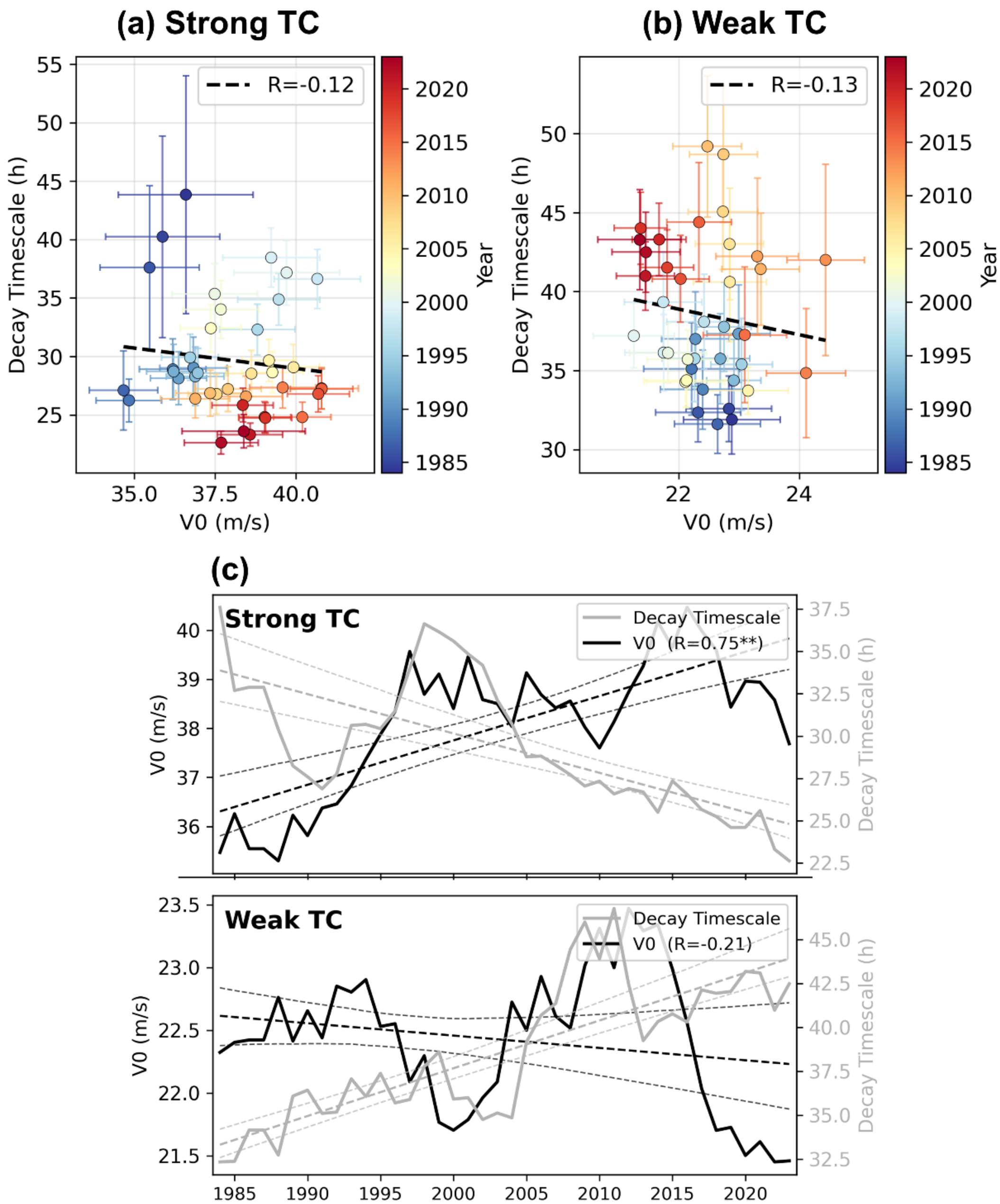

Extended Data Fig. 11. *Scatter plots between annual-mean decay timescale and landfall intensity for **(a)** strong and **(b)** weak tropical cyclones, with dot color indicating year and error bars denoting ±1 standard error in five-year window. The dashed line shows the ordinary least-squares regression fit, with the Pearson correlation coefficient (R) and significance level indicated (* P < 0.05, ** P < 0.01). **(c)** Time series of landfall intensity (black line) and decay timescale (gray line) for strong tropical cyclones (upper sub-panel) and weak tropical cyclones (lower sub-panel).*

2. Your intensity changes are considered within 24 hours after landfall, but the TC precipitation is considered within 72 hours after landfall? Suggest that these two times should be consistent.

We appreciate your constructive suggestion. We agree that using 72-hour accumulated precipitation could introduce influences from outer rainbands and remnant circulation that are less directly linked to the post-landfall intensity decay analyzed in this study. We have therefore revised the precipitation analysis to use 24-hour accumulated precipitation after landfall, consistent with the 24-hour window used for the decay timescale calculation. The results are very similar, confirming that our conclusions regarding the coast-to-inland precipitation redistribution are robust. The manuscript text and figures have been updated accordingly.

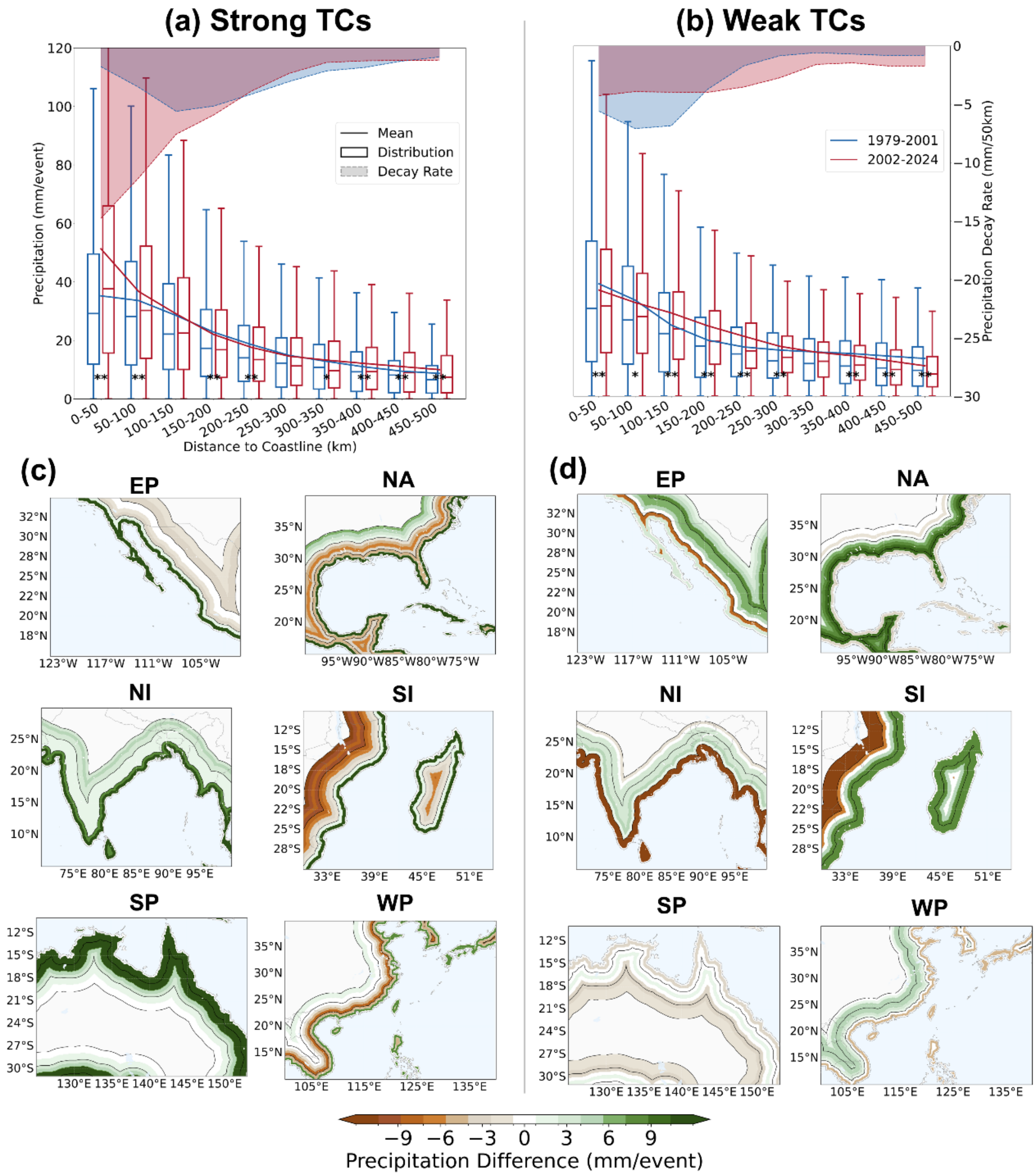

Fig. 3. Landfall tropical cyclone precipitation patterns show distinct coast-to-inland changes. ***(a, b)*** *24hours accumulated precipitation after landfall along the distance to coastline*

for strong and weak tropical cyclones. Lines and box plots show the mean and distribution of precipitation generated by tropical cyclone events along distance to coastline. Asterisks denote statistically significant differences of precipitation between periods (P < 0.05, ** P < 0.01). Shaded areas represent patterns of precipitation decay rate with increasing distance from coastline.* ***(c, d)*** *Precipitation invasion changes (2002–2024 minus 1979–2001) along the 500km coastal band width across six basins. Contours represent area-averaged changes within distance zones. Positive value (in green) indicates increased precipitation, negative values (in brown) indicate decreased precipitation.*

3. In your response, you mentioned that decay timescale (τ) was chosen as the primary metric because it normalizes for the strong dependence of absolute intensity loss on landfall intensity. In table 1, why is it still significant between weak TCs and landfall intensity after removing the impact of landfall intensity?

Thank you for this question. We wish to clarify that the decay timescale normalizes the strong first-order dependence of absolute wind speed loss on landfall intensity. For example, a storm losing 20 m/s during the first 24 hours is not necessarily decaying faster than one losing 10 m/s if the former started at 60 m/s and the latter at 15 m/s. This normalization makes storms of different landfall intensities directly comparable in terms of their relative decay behavior, which is why decay timescale was chosen as the primary metric in this study and many research focus on tropical cyclone post-landfall decay.

However, normalization of absolute intensity loss does not imply that decay timescale is entirely independent of landfall intensity across all intensity ranges. The event-scale correlation between decay timescale and landfall intensity for weak tropical cyclones ($R = 0.24$) reflects a physically meaningful secondary relationship: as demonstrated by CM1 model simulations, weaker storms possess shallower and less organized vortex structures with lower frictional energy dissipation rates that decrease nonlinearly with intensity, enabling them to sustain unorganized circulation longer after landfall. This is physically consistent with the broad exponential relationship between landfall intensity and decay timescale shown in Extended Data Fig. 1a.

Critically, this correlation does not compromise our main findings. The year-scale correlation between annual mean decay timescale and landfall intensity is insignificant for both strong and weak tropical cyclones (new Extended Data Fig. 11), indicating that the divergent long-term trend in decay timescale is not primary driven by changes in landfall intensity. This is further confirmed by the inconsistency between the temporal trends in intensity and decay timescale, pointing instead to environmental changes as key drivers.

4. In figure 2d, you analyzed the characteristics of the landfall latitude, but I didn't see the necessity of analyzing landfall latitude in the article. Suggest adding some description and discussion.

Thank you for raising this point. The analysis of landfall latitude in Fig. 2d serves two important purposes in our study. First, it demonstrates that the intensity-dependent bifurcation extends beyond decay timescale to other storm characteristics, reinforcing the broader paradigm of intensity-dependent heterogeneity in tropical cyclone behavior and hazard. Second, the difference in latitudinal trends between strong and weak tropical cyclones, particularly the

divergent evolution after 2000, suggests that the two groups are increasingly experiencing distinct environmental regimes at landfall. For instance, the equatorward retreat of strong tropical cyclone landfall locations in recent 20 years places them in regions of amplified VWS increases linked to tropical expansion and upper-tropospheric warming.

These latitudinal changes thus provide important physical context for the divergent environmental sensitivities documented in Table 1 and Fig. 4, and help explain why the two groups respond differently to a changing climate. We have added the following sentence to the Results section (Line 170-171): These contrasting latitudinal evolutions, especially after 2000, suggest potential distinct environmental landfall regimes.

Response to Reviewer 3

Regarding my major concern about the driving causes behind the diverging trends between strong and weak storms, I am still not fully convinced.

First, I understand that the analysis is based on grouped strong and weak storms. However, I do not think this necessarily rules out the influence of trends in storm intensity itself. Potential changes in TC intensity and structure could be important factors contributing to the observed decay trends.

Thank you for raising this important point. We admit that the intensity, structure, and post-landfall decay are physically interconnected. Therefore, we design additional analysis including co-trend analysis, and partial correlation analysis to directly test whether trends in storm intensity itself could explain the observed divergent decay trends. We find that changes in landfall intensity are not the primary driver of the observed decay trends.

The late period (2002–2024) does contain a higher proportion of strong tropical cyclones compared to early period (1979–2001, Fig. A1), consistent with global warming-driven intensification of storm intensity. However, the event-scale correlation between landfall intensity and decay timescale is only $R = 0.06$ ($P > 0.05$) for strong tropical cyclones. While weak tropical cyclones show a higher event-scale correlation ($R = 0.24$, $P < 0.01$), the shift in their landfall intensity distribution is minimal. At the year scale (new Extended Data Fig. 11), correlations between landfall intensity and decay timescale are insignificant for both strong ($R = -0.12$, $P > 0.05$) and weak storms ($R = -0.13$, $P > 0.05$), and their temporal evolutions are decoupled: strong-storm intensity rose most during 1984–2000 while decay timescale showed no consistent trend, then declined sharply post-2000 while landfall intensity change modestly; weak-storm intensity showed a negligible long-term trend even as decay timescale rose sharply during 2000–2012, then enhanced slightly after 2015 while mean intensity fell rapidly.

Partial correlation controlling for landfall intensity confirms this: the decay timescale trend remains highly significant and nearly unchanged for both strong (partial $R = -0.68$ vs. raw $R = -0.70$) and weak storms (partial $R = +0.77$ vs. raw $R = +0.72$, all $P < 0.01$), indicating intensity accounts for little of the trend.

Overall, we do not dismiss modest influences of storm intensity changes, but the additional analysis confirms that they cannot be the primary driver of the bifurcation in decay trends.

Rather, it is better explained by the distinct environmental sensitivities and evolution between strong and weak storms.

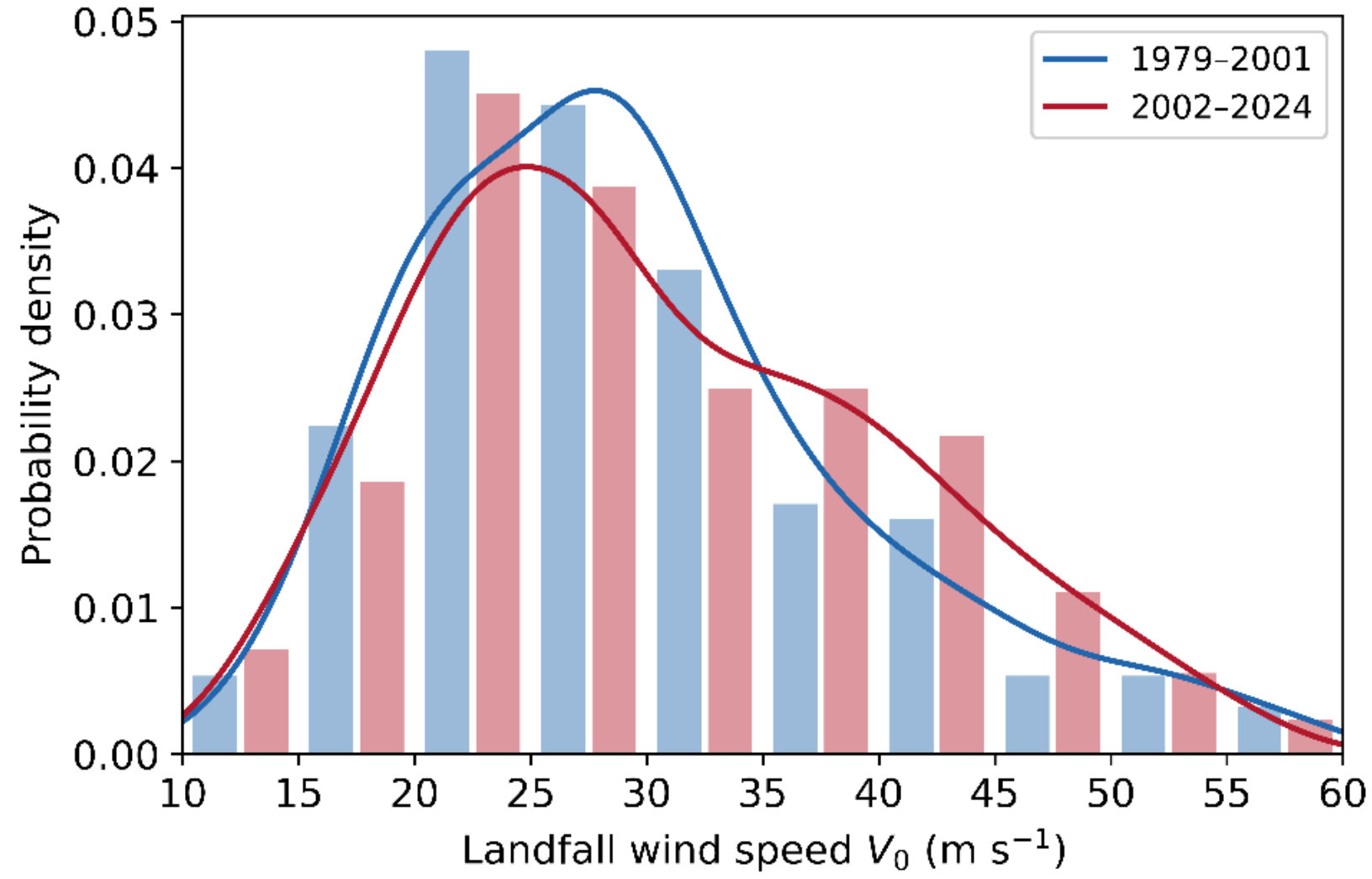

Fig. A1. *Probability density distributions (PDF) of landfall intensity for the early (blue, 1979–2001) and late (red, 2002–2024) periods.*

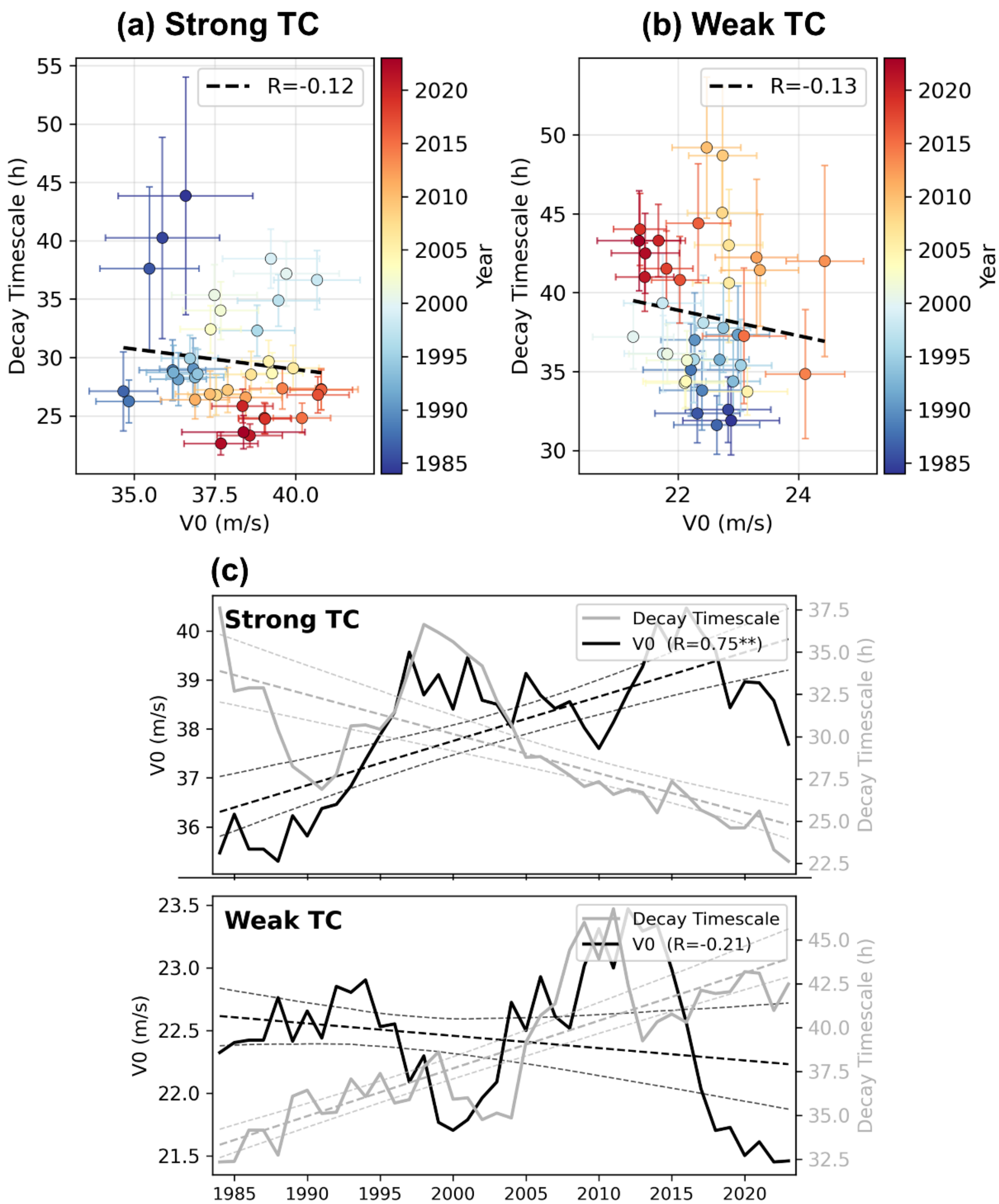

Extended Data Fig. 11. *Scatter plots between annual-mean decay timescale and landfall intensity for* ***(a)*** *strong and* ***(b)*** *weak tropical cyclones, with dot color indicating year and error bars denoting ±1 standard error in five-year window. The dashed line shows the ordinary least-squares regression fit, with the Pearson correlation coefficient (R) and significance level indicated (* $P < 0.05$, ** $P < 0.01$).* ***(c)*** *Time series of landfall intensity (black line) and decay timescale (gray line) for strong tropical cyclones (upper sub-panel) and weak tropical cyclones (lower sub-panel).*

Second, while the sensitivity analysis is informative, I do not think it necessarily establishes a cause-and-effect relationship.

We thank you for this comment. Except for sensitivity analysis, this study provides mechanistic support through three different investigations, including co-tend analysis, trend decomposition, and CM1 numerical model simulations.

First, we examined the temporal co-evolution of decay timescale and key environmental variables through detailed trend analysis (Extended Data Fig. 8), demonstrating that long-term trends in VWS, translation speed, soil moisture, SST, and atmospheric moisture closely track the divergent decay timescale trends, with significant year-scale correlations ranging from R = 0.53 to 0.75 for strong and R = 0.45 to 0.71 for weak storms (all $P < 0.01$).

Second, we employed a two-step trend decomposition framework (Fig. 4g, h) to quantify the fractional contribution of key environmental factors to the observed long-term decay trends, demonstrating that key environmental variables collectively explain 46%–65% of the decay trends. This approach provides a quantitative, multi-variable account of the observed divergent trends.

Third, we conducted idealized CM1 numerical experiments that provide direct process-level evidence for the intensity-dependent environmental sensitivities (Fig. 5). In a controlled setting where all other factors are held constant, model outputs confirm that stronger storms are substantially more sensitive to VWS-induced structural disruption while weaker storms respond more strongly to SST warming. This difference in sensitivity, combined with the distinct observed environmental trends, is physically consistent with the observed divergent decay trends.

The physical consistency is further supported by prior region-specific studies that have established links between individual environmental factors and intensity decay, including the roles of SST warming [9, 10], translation speed [25], soil moisture [29, 55], and atmospheric stability [20–22, 25], which our global, intensity-stratified analysis corroborates and extends. We believe the combination of observational sensitivity analysis, co-trend analysis, multivariate trend decomposition, and idealized numerical experiments represents a rigorous and appropriate level of mechanistic support for our primary contribution: the identification of the global intensity-dependent bifurcation in post-landfall decay trends, and its physical consistency with differences in storm structure and environmental sensitivity.

Third, I was wondering whether you have examined any trends in the observed VWS.

Thank you for this comment. We did investigate the trends in observed VWS. As Extended Data Fig. 8c shown, the temporal co-evolution of annual-mean VWS and strong tropical cyclone decay timescale shows a high degree of consistency, with significant year-scale correlation (R = −0.53, $P < 0.01$; Fig. 4c). Extended Data Fig. 9b further shows the global spatial distribution of VWS trends, demonstrating that VWS has intensified across most subtropical basins in recent decades, particularly over the South China and the southern United States. The corresponding description is provided in the main text (lines 313–318). This part of VWS trend analysis forms a core component of our mechanistic framework.

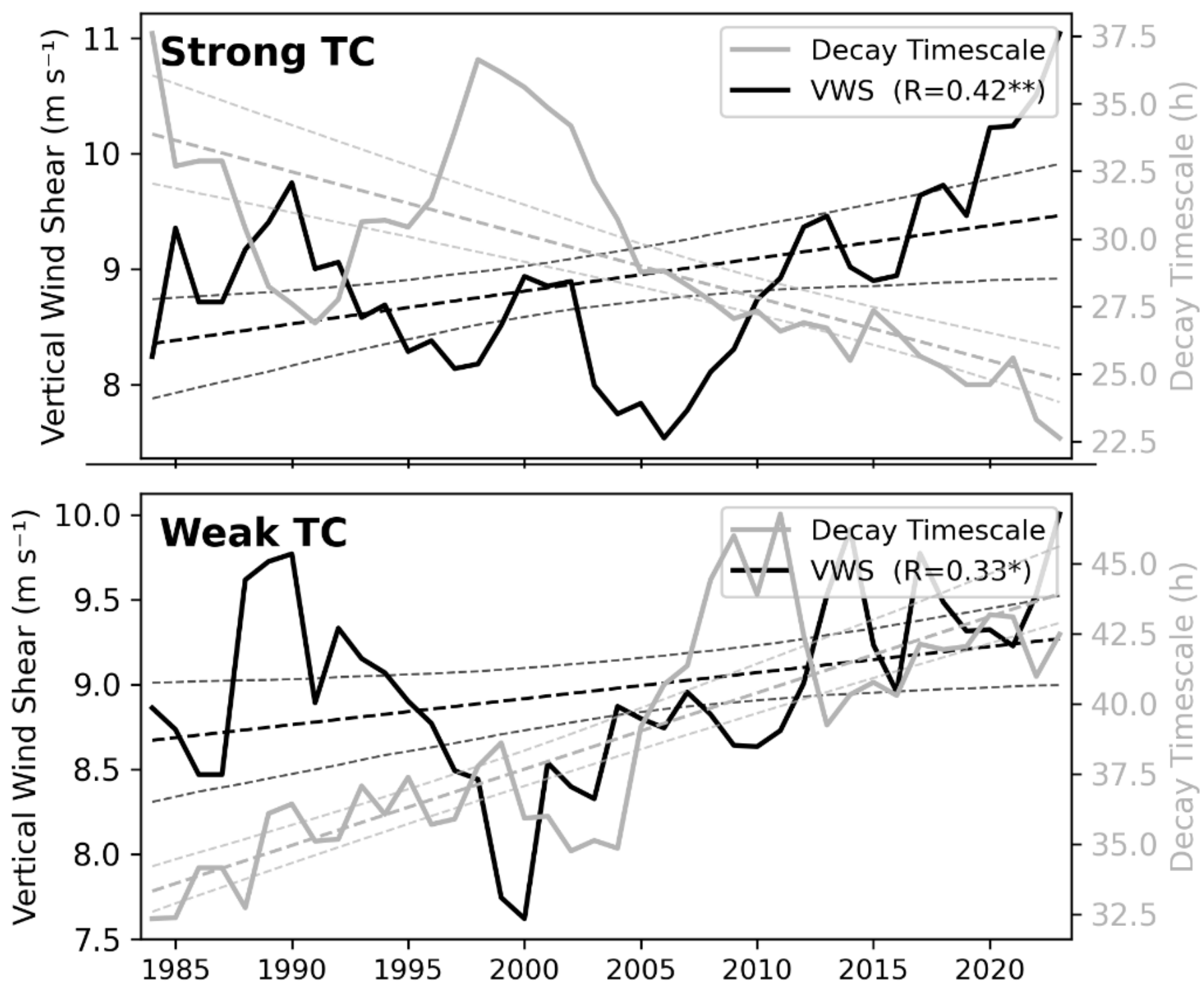

Extended Data Fig. 8c. *Time series of vertical wind shear combined with tropical cyclone decay timescale for strong and weak tropical cyclones.*

Overall, I remain concerned that the mechanism underlying the observed trends has not yet been fully explained.

We understand your concern about fully investigating the mechanism behind the observed divergent decay trends. A more comprehensive mechanistic attribution would require large-ensemble climate model experiments. However, it lies far beyond the scope of this observation-based study. The primary objective of this study is to provide observational evidence for an overlooked intensity-dependent bifurcation in global tropical cyclone post-landfall decay trends, and to demonstrate its physical consistency with structural differences between strong and weak storms and the observed evolution of key environmental factors.

To verify the mechanism analysis, we have included CM1 model simulations into our analysis to provide process-level mechanistic evidence, building upon observation-based sensitivity and trend analyses. We believe the combination of observations and numerical models represents a rigorous and appropriate level of mechanistic support. We hope you could agree on the novelty of this study which guide future efforts towards a deeper understanding of the intensity-dependent bifurcation effect. We have suggested future work for attribution through large-ensemble climate model experiments in the Discussion: Future work extending this framework to large-ensemble CMIP6 experiments will be essential for formal attribution and for assessing whether the intensity-dependent bifurcation amplifies under different emission scenarios.
